# Process Dependent Complexity in Multicomponent Gels

**DOI:** 10.1002/marc.202200709

**Published:** 2022-10-18

**Authors:** Rebecca I. Randle, Rebecca E. Ginesi, Olga Matsarskaia, Ralf Schweins, Emily R. Draper

**Affiliations:** ^1^ School of Chemistry, Joseph Black Building University of Glasgow Glasgow G12 8QQ UK; ^2^ Institut Laue‐Langevin Large Scale Structures Group 71 Avenue des Martyrs, CS 20156 Grenoble CEDEX 9 F‐38042 France

**Keywords:** co‐assembly, gels, low molecular weight gelators, self‐assembly, self‐sorting

## Abstract

Mixing low molecular weight gelators (LMWGs) can be used to combine favorable properties of the individual components within a multifunctional gel. Such multicomponent systems are complex enough in themselves but the method of combining components is not commonly considered something to influence self‐assembly. Herein, two multicomponent systems comprising of a naphthalene‐based dipeptide hydrogelator and one of two modified naphthalene diimides (NDIs), one of which forms gels, and the other does not, are investigated. These systems are probed, examining the structures formed and their gel properties (when preparing a solution from either a mixed powder of both components or by mixing pre‐formed solutions of each component) using rheology, small angle neutron scattering (SANS), and absorbance spectroscopy. It is found that by altering the method of preparation, it is can either induce self‐sorting or co‐assembly within the fibers formed that underpin the gel network.

## Graphical abstract

Self‐sorting and co‐assembled gels can be controlled by the pre‐mixing of components and using the same gelation conditions. The co‐assembled vs. self‐sorted gels have different properties despite using the same molecules and the same gelation method.

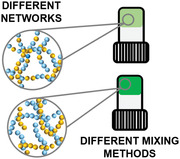

## 1 Introduction

Low molecular weight gelators (LMWGs) self‐assemble and form gels through the entanglement or crosslinking of fibers.^[^ ^1^, ^2^, ^3^ ^]^ The aggregates and gel networks themselves are held together by non‐covalent forces such as van der Waals forces and hydrogen bonding^[^ ^2^, ^3^ ^]^ and so are greatly influenced by factors such as pH^[^ ^4^, ^5^, ^6^ ^]^ and solvent.^[^ ^7^, ^8^ ^]^ Mixing two LMWGs together has many advantages such as combining the desirable properties of each component,^[^ ^9^, ^10^, ^11^ ^]^ for example combining a stiffer gelator with a softer but electronically‐active gelator to improve rigidity, or combining p‐type gelators with n‐type gelators to create a bulk heterojunction.^[^ ^12^, ^13^ ^]^ Combining gelators has reported applications in optoelectronics,^[^ ^14^, ^15^, ^16^ ^]^ photo‐patterning gels,^[^ ^17^, ^18^ ^]^ and in biological fields.^[^ ^19^, ^20^, ^21^, ^22^ ^]^

**Figure 1:**
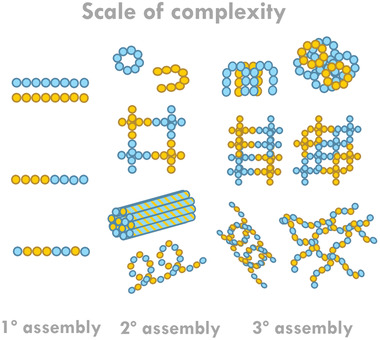
Cartoon illustrating hypothetical examples of the different levels of complexity in a two‐component system from the primary assembly up to the tertiary assembly level.

If both components can form a gel, there are many possible types of fibrous networks that may be created.^[^ ^20^, ^23^, ^24^ ^]^ At the first level of assembly (**Figure** **1**), the two components can form self‐sorted, socially self‐sorted, or randomly co‐assembled fibers. At the next level, we need to consider whether these fibers will assemble into homo‐ or hetero‐aggregates. Then, on a larger scale, various microstructures and networks are possible. If one system forms gel networks before another, this will have an impact upon further self‐assembly (Figure 1).^[^ ^25^ ^]^ Even when the gelators do not co‐assemble, there is also the possibility that one component will influence how the other system assembles.^[^ ^18^, ^26^, ^27^, ^28^ ^]^ There are even examples of non‐gelating components incorporating into gelling fibers^[^ ^12^, ^23^ ^]^ and examples of gel networks being significantly altered when forming in the presence of a non‐gelling component.^[^ ^29^, ^30^ ^]^ All this shows the high level of complexity which is often difficult to predict as well as difficult to characterize fully. Therefore, understanding both the primary and larger scale structures is important for the overall properties, and even more important when trying to design systems for specific purposes.

Naphthalene diimides (NDIs) are photo‐ and electrochromic molecules,^[^ ^11^, ^14^, ^31^, ^32^, ^33^, ^34^, ^35^, ^36^ ^]^ with some examples being able to form hydrogels.^[^ ^17^, ^37^, ^38^, ^39^, ^40^, ^41^, ^42^, ^43^, ^44^, ^45^ ^]^ We have previously reported a dipeptide functionalized NDI hydrogelator that forms soft photo‐ and electrochromic gels.^[^ ^17^ ^]^ For an application where a material would be manually handled, a soft gel would not be suitable. This problem could be remedied with the addition of another component to provide an increased degree of stiffness. A range of stiff transparent gelators with similarly functionalized dipeptides have been reported.^[^ ^43^, ^45^, ^46^, ^47^, ^48^, ^49^, ^50^, ^51^, ^52^, ^53^ ^]^ Using these gelators in conjunction with NDIs would not compromise the water solubility or color of the system. In this approach, a “best of both worlds” could potentially be achieved, but as highlighted in Figure 1, there is a lot to consider in terms of assembly, rather than just mixing the properties. We recently described in our previous work the importance of the aggregated state upon coloration of the NDIs,^[^ ^54^ ^]^ and so any effect of a second component on the NDI self‐assembly could significantly impact the performance of the material.

There are numerous reports of multicomponent gel systems.^[^ ^22^, ^23^, ^24^, ^55^, ^56^, ^57^, ^58^, ^59^, ^60^, ^61^, ^62^ ^]^ Techniques such as imaging and nuclear magnetic resonance (NMR) spectroscopy have been used to assess assembly.^[^ ^12^, ^63^, ^64^, ^65^, ^66^ ^]^ However, the way the components are mixed is not often highlighted. For example, combining two water‐soluble components can be carried out by simply mixing an equal weight of each solid together in water, or by combining two preprepared solutions (of higher concentration) together to allow the dilution to the desired final concentration. One could imagine the solution mixed materials could have larger scale structures “locked‐in” before gelation is triggered, whereas starting from powders of each could result in co‐assembly or different structures formed.

**Figure 2:**
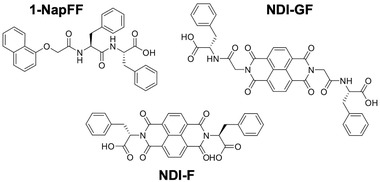
Chemical structure of the molecules used in this study.

Here, we report how the method of combination of the gelator, a naphthalene moddified with a dipeptide (**1‐NapFF)**,^[^ ^43^ ^]^ with an NDI modified with an amino acid (**NDI‐F**, which does not form gels^[^ ^54^ ^]^) or a dipeptide (**NDI‐GF**, which does form gels^[^ ^17^ ^]^) can impact the self‐assembled structures formed at both high pH and in the gel state. The chemical structures of **1‐Nap‐FF**, **NDI‐F,** and **NDI‐GF** are shown in **Figure** **2**.

## 2 Results and Discussion

**Figure 3:**
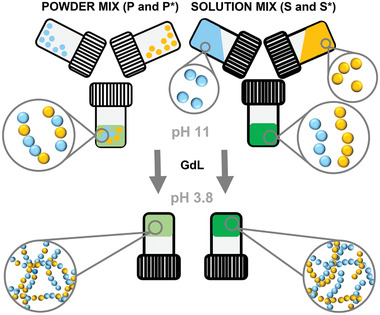
Cartoon showing the two different methods of preparation investigated in this study. The different colors represent different molecules used.

Multicomponent gels were prepared using **1‐NapFF** with either **NDI‐F** or **NDI‐GF**, in each case at a ratio of 5:5 mg mL^−1^. Solutions were initially prepared at pH 11 and then gels were formed using glucono‐*δ*‐lactone (GdL) to enable a slow homogeneous pH switch.^[^ ^67^ ^]^ Two different methods of preparation of the solutions at high pH were used (**Figure** **3**). The first method involved preparing separate solutions of each of the components at 10 mg mL^−1^ in water each using two equivalents of sodium hydroxide (NaOH). These solutions were then mixed in a 1:1 ratio to afford a solution with a final concentration of 5 mg mL^−1^ of each component at pH 11. These solutions are referred to here as solution mixed or **S** solutions. The second method involved mixing the components as powders in the same vial and then adding two equivalents of NaOH and water to give a solution at a final concentration of 5 mg mL^−1^ of each component at pH 11. These are referred to as powder mixed or **P** solutions. In these solutions, one would imagine there would be more opportunity for co‐assembly to occur as the molecules are not dispersed heterogeneously in solution. The different multicomponent solutions prepared from **1‐NapFF** with **NDI‐GF** are referred to as **S** or **P** whereas mixes of **1‐NapFF** with **NDI‐F** will be referred to as S* or P*. Full experimental protocols are described in the Supporting Information and Table S1.

First, we examined the structures formed in both solutions at high pH. All individual components formed worm‐like micelles, which is evidenced by shear thinning behavior^[^ ^53^ ^]^ (Figures S1, Supporting Information). At high pH, both **S** and S* mixes have a much higher viscosity than the individual components. This change in viscosity suggests an interaction between components.^[^ ^1^, ^54^ ^]^ This observation is also seen for **P** and P* but the effect is not as pronounced, Figures S2–S3, Supporting Information, suggesting that bulk viscosity is influenced by the preparation method.

The structures in these solutions were investigated using small angle neutron scattering (SANS). The data for the solutions of **NDI‐GF** alone at pD 11 (pD due to deuteration of solvent), were fit to a flexible elliptical cylinder model with a large axis ratio implying that the self‐assembled structures present are tape‐like flat cylinders (Figure S4 and Table S2, Supporting Information).^[^ ^68^ ^]^ The data for **1‐NapFF** alone at pD 11 fit to a hollow cylinder model and the data is comparable with that previously reported (Figure S5 and Table S3, Supporting Information).^[^ ^8^, ^46^, ^69^ ^]^ **S** at high pD is best fit by combining a hollow cylinder and a flexible elliptical cylinder model, implying the data represent a simple addition of the two single components with dimension remaining similar to that of the individual components and hence self‐sorting (Figure S6 and Table S4, Supporting Information). In comparison, **P** fits best with a flexible elliptical cylinder model alone, showing that a new structure is formed (Figure S7 and Table S5, Supporting Information). These data show that at high pD there is a difference in aggregation where the structures of **1‐NapFF** in **P** have been disrupted by **NDI‐GF**, or the two components are co‐assembling.

**NDI‐F** alone at high pD fits to a flexible elliptical cylinder with a power law (with a high axis ratio much like **NDI‐GF**), Figure S8 and Table S6, Supporting Information). Both S* and P* at pD 11 fit to a combination of hollow and flexible elliptical cylinders implying self‐sorting is occurring (Figures S9–S10 and Tables S7–S8, Supporting Information.

**Figure 4:**
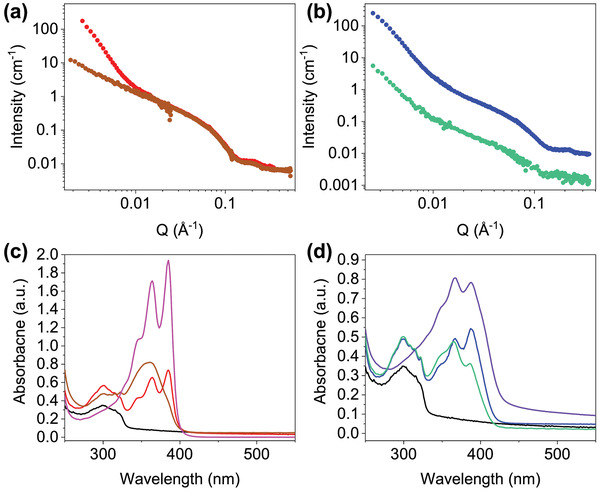
SANS from a) P* (brown) and S* (red) solutions and b) **P** (turquoise) and **S** (blue) solution. UV‐Vis absorption spectra of **1‐NapFF** (black) (c) P* (brown) and S* (red) gels and (d) **P** (turquoise) and **S** (blue) gels. c) **NDI‐F** solution at pH 6 (pink) and d) **NDI‐GF** gel (purple) spectra were also shown.

The scattering intensities of **S** and S* are comparable to a simple addition of scattering profiles of **1‐NapFF** and the respective NDI (Figures S11a–S12a, Supporting Information). P* is also comparable to this in the mid to high Q region (Figure S12b, Supporting Information). The scattering in **P** is much weaker than **S** and **1‐NapFF** across all regions of Q and is more comparable to **NDI‐GF** (Figure S11b, Supporting Information). This difference in scattering intensity between preparation methods is more pronounced in the two gelator systems, **Figure** **4**a,b, which suggests that even at high pD, there may be co‐assembly leading to smaller aggregates and weaker scattering.

Next, we formed gels from each of the solutions by the addition of GdL to produce gels at a final pH of 3.8 to determine whether the differences in structure persisted in the gel phase First, we examined the apparent p*K*_a_ values of the mixed systems. These values correspond to the p*K*_a_ of the aggregates and are typically linked to the p*K*_a_ at which self‐assembly starts to occur.^[^ ^45^, ^70^ ^]^ If self‐sorting is occurring, then it would be expected that there would be no change in the apparent p*K*_a_ of either component in the mixed system. If different structures were formed or co‐assembly were occurring, a shift in the apparent p*K*_a_ values would be expected. For the NDI systems, the p*K*_a_ values correspond to the protonation/deprotonation of the two carboxylic acid groups of the amino acid moieties. For **1‐NapFF**, the presence of a second p*K*_a_ has been linked to a change in structure as pH is lowered for a closely‐related molecule.^[^ ^53^ ^]^ When combined, the apparent p*K*_a_ values of the NDIs are found at the approximate value of the single components. The first apparent p*K*_a_ of **NDI‐F** is more significantly shifted to a higher value in the mixtures (Figures S13–S19 and Table S9, Supporting Information). The first p*K*_a_ value associated with **1‐NapFF** is shifted to a higher value in **S**, **P** and S* (Table S9, Supporting Information), showing that the pH at which changes in structure occur for the **1‐NapFF** and **NDI‐F** is affected by the addition of a second component and by preparation method.

On decreasing the pH, each multicomponent system (and the single component **1‐NapFF** and **NDI‐GF** systems) forms gels that are frequency independent and break at low strain (Figures S20–S31, Supporting Information) suggesting that the components do not disrupt each other sufficiently to stop the formation of large‐scale networks, even with a non‐gelling component present.^[^ ^71^ ^]^ The break point or yield point is defined as the point at which the gels are no longer showing a linear viscoelastic behavior, and so the value is taken as the point at which G′ deviates from linearity upon increasing strain.

Using absorbance spectroscopy, we can observe that the spectra of **S**/S* and **P**/P* gels are comparable to the positions to **NDI‐GF**/**NDI‐F** and **1‐NapFF** alone (Figure 4c,d). There is a difference in the peak ratios in the NDI component (at 365 and 385 nm) in **NDI‐GF**, **S** and **P** gels, suggesting a difference in molecular packing of the NDI component in each system (Figure 4d). The ratio of peaks is 1:0.96, 0.91:1 and 1:0.55 for 365:385 nm in systems of **NDI‐GF**, **S** and **P** respectively (Figure S32, Supporting Information). **NDI‐GF** and **S** are more comparable than **P**. These data suggest that the aggregates are self‐sorted in **S** but may be co‐assembling in **P**. Gels of **1‐NapFF**, **S** and **NDI‐GF** (but not **P**) undergo a small bathochromic shift during gelation (Figures S33–S36, Supporting Information) and is most significant in **NDI‐GF** (Figure S34, Supporting Information).^[^ ^72^, ^73^, ^74^, ^75^ ^]^ The molecular packing of **NDI‐GF** also changes during gelation (Figures S37–S39, Supporting Information).

For S* and P*, the influence of preparation method is more significant than in **S** and **P**. The spectrum of S* appears as an overlay of components whereas that of P* lacks definition between 365 and 385 nm, which suggests that S* is more likely to be self‐sorted than P* (Figure 4c, consistent with previous discussion). A small shoulder at 385 nm is seen in spectra at high pH of P* which suggests subtle changes to packing as gelation occurs (Figure S39, Supporting Information). No change is observed in **S** and shifting in peaks is seen with gelation unlike what is observed for the **NDI‐GF** systems (Figure S40, Supporting Information).

**Figure 5:**
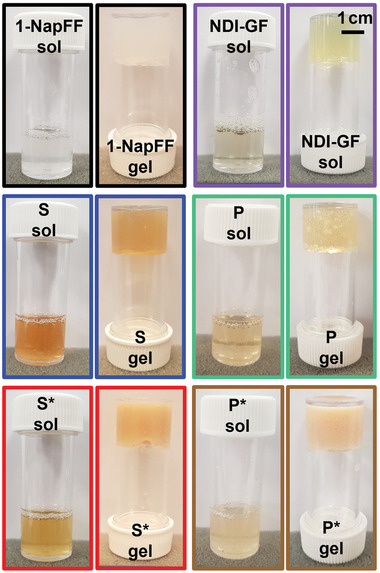
Photographs of solutions at pH 11 and their corresponding gels.

As single component systems, **1‐NapFF** forms opaque gels and **NDI‐GF** forms yellowish gels under these conditions (**Figure** **5**).

**Figure 6:**
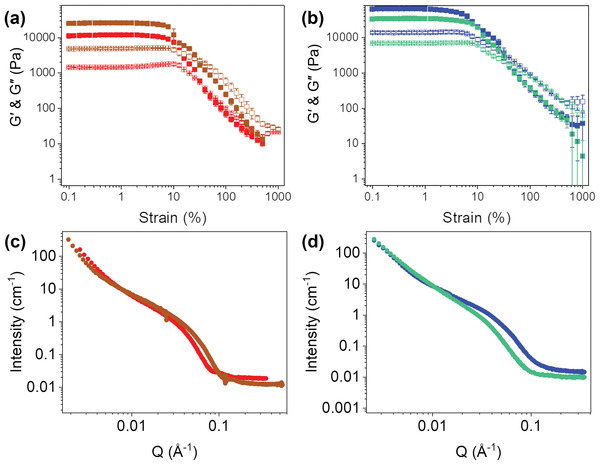
Rheological strain sweeps at frequency of 10 s^−1^ a,b) SANS data c,d) for **S** (blue), **P** (turquoise), S* (red) and P* (brown) gels. *G*′ are the solid shapes and *G*″ are the empty shapes. Rheological experiments were measured at 10 rads^−1^ in triplicate and error was calculated from their standard deviation.

When analyzing the bulk rheological data, the two gelator systems formed stiffer gels than gels formed by the single component systems (Figures S42a and Table S10, Supporting Information). We would expect increased stiffness as the overall concentration of the system has increased. Therefore, not much can be taken from this observation, but the change in the yield and flow point indicates a change in network or fibers (Table S10, Supporting Information).^[^ ^18^, ^23^, ^60^ ^]^ Gel strength and stiffness are influenced by the preparation method (**Figure** **6**a,b and Table S10, Supporting Information). In all mixed gels, the stiffness is dominated by the **1‐NapFF** component (which alone has a G′ of 18 kPa), but two gelator systems are stiffer (G′ of 66 and 35 kPa for **S** and **P** respectively).

The yield point for **P** is the same as 1‐NapFF alone at 6% strain, whereas the yield point of **S** is weaker at 4% strain. Neither gel shows an increase in G″ before breaking (Figure 6b and Figure S42a, Supporting Information) as seen in the single components, which suggests that the gel networks formed are different to either of the single components. P* gels are slightly stiffer than 1‐NapFF alone (G″ of 26 kPa) and S* gels are less stiff (12 kPa), Table S10, Supporting Information. Both have comparable yield points to **1‐NapFF** of 6% but S* has a higher flow point of 40% strain, than **1‐NapFF** and P* which are both at 32% strain, Table S10, Supporting Information. Only **1‐NapFF** and S* show a slight rise in G″ as the gel begins to break, which suggests that **NDI‐F** disrupts the network of **1‐NapFF** more when prepared in a non‐homogeneous environment (P*), Figure 6a and Figure S42b, Supporting Information.

Scattering data from a **1‐NapFF** gel fit to hollow cylinder model with a total radius of 15 Å combined with a power law, Figure S43 and Table S11, Supporting Information. The data for the **NDI‐GF** gel fit to a flexible elliptical cylinder with a radius of 13 Å, Figure S44 and Table S12, Supporting Information. Both **S** and **P** gels fit to a flexible elliptical cylinder with a power law, differing in radius with 24 Å and 34 Å respectively, Figures S45–S50 and Tables S13–S14, Supporting Information. **S** gels have more similar features to that of the single components, which could suggest that the gel structures have aligned, templated each other or are intimately mixed, as shown by the doubling in radius. **P** gel fits are less similar to the individual components and are more than double in radius, suggesting a co‐assembled new fiber has been formed. However, this data alone does not prove self‐sorting or co‐assembly in the fibers but does show differences in structures at high pD leads to differences in bulk gel properties and other structure parameters such as radius and length in the gels (Tables S13–S14, Supporting Information).

The data for **NDI‐F** at low pD fit to a hollow cylinder with a power law with a radius of 42 Å, Figure S54 and Table S15, Supporting Information. For S* and P* both gels have a similar radius of 35 Å (Figures S49–S52 and Tables S16–S17, Supporting Information). S* gels fit to a combination of hollow cylinder and flexible cylinder, whereas P* fits to a flexible cylinder with a power law. The presence of the two models in S* gels could suggest self‐sorting is occurring, whereas one model in P* suggests co‐assembly has occurred. The observation from all systems demonstrates that the presence of an NDI species, even a non‐gelating one, has influence over the networks of **1‐NapFF**.

**Figure 7:**
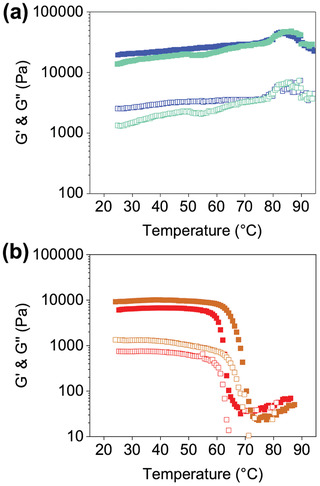
Hydrogel temperature sweeps for a) **P** (turquoise data) and **S** (green data) b) P* (red data) and S* (brown data). G′ are the solid shapes and G″ are the empty shapes. Tests performed at 0.5% strain and 10 rads^−1^.

To test further whether the **S** gels had a higher propensity to form self‐sorted fibers and **P** gels co‐assembled fibers, we used the melting points of the gels. True melting point is defined as when the gel is no longer acting as a viscoelastic material, and so would flow. This could be indicative of fibers untangling, dehydrating, disassociating, aggregates falling apart due to increased solubility and then eventually becoming liquid like again or even precipitating. However, what happens during this process completely depends on the solubility of the molecules, how they are assembled and then how the network has been formed. Due to the variety in what could be happening during heating this results in changes in G′ or G″, and so increases and decreases in values can be seen. The melting point here is defined as when there is a change in G′ or G″. From this we can also determine if the fibers were self‐sorted and not effected by the presence of another network, one would expect to see separate melting points for each of the components. If co‐assembly had taken place, we would expect to see one single melting point or completely different melting profile.^[^ ^76^ ^]^ For the case of **1‐NapFF** alone there were two transitions at 55 and 83 °C and for **NDI‐GF** one transition at 72 °C (Figure S55, Supporting Information). **NDI‐F** does not have a gel melting point as it does not gel. The **S** gel shows three transitions when the temperature of the gel is increased there are three transitions at 55, 70, and 84 °C, which supports the other data collected that this system is self‐sorted. Conversely the **P** gel only has one transition at 80 °C, again support the theory that is co‐assembled (**Figure** **7**a and S56a,b, Supporting Information).

Both S* and P* gels have one melting point each at 58 and 65 °C respectively (Figures 7b and S56c and S56d, Supporting Information). The character of the **1‐NapFF** has been lost, meaning that the network has been disrupted by the presence of the non‐gelling component, this was seen in the SANS data. It is more difficult from this data to determine whether they are self‐sorted or co‐assembled. We hypothesized that the structures formed in solution were kinetically trapped and therefore given time, the aggregates in the **P**/P* solutions could change to become comparable to the **S**/S* solution. A **P** and P* solution were therefore left to age, and an aliquot used to prepare gels every two weeks for (Figures S57–S72 and Table S18–S21, Supporting Information). Single components were also aged for comparison. **P** gels do change with aged solutions, but not comparable to **S** gels. The results were found to be similar to the ageing of **1‐NapFF** alone (Figures S55–S62 and Table S18–S19, Supporting Information). After ten weeks, gels of P* were comparable to the gels formed when solutions were freshly prepared (with some subtle changes likely a result of aging solution as rheological trends were comparable to **1‐NapFF** ageing), Figures S62–S64 and S68–S72 and Tables S19 and S21, Supporting Information. Therefore, we concluded that this was not simply a case of kinetically trapped structures in the **P** samples converting to **S** structures overtime. Instead, the **P** samples are using a completely different assembly pathway to form structures with different mechanical, optical and morphological properties to that of the **S** samples. More discussion about this aging study is found in Supporting Information.

## 3 Conclusion

Multicomponent systems of **NDI‐F** or **NDI‐GF** and **1‐NapFF** and their gelation processes have been characterized by a range of techniques. We observed significant variation in properties when components are combined in different ways. Sample preparation was shown to change visual color, molecular packing and aggregation of both solution and gels. Consequently, networks and bulk properties are affected.

When components can assemble before combination, self‐sorted structures are formed. Whereas when components are mixed together as solids, we hypothesize the likelihood of co‐assembled or random fibers increases. Data from one and two gelator multicomponent systems support this hypothesis. This was even more apparent in the P* and S* when only one component was a gelator. The preparation method of multicomponent systems is a factor that is often overlooked in multicomponent studies and has a significant impact upon self‐assembling systems at different length scales, even when only one component can form gels. It also opens up new possibilities for structures and properties using the same systems.

## 4 Experimental Section

Full protocol and experimental procedures can be found in the Supporting Information.

### Preparation of Solutions

Single component solutions were prepared at concentrations 5 mg mL^−1^. Solids were dissolved in one or two molar equivalents of aqueous NaOH (0.1 M) for 1‐NapFF and thNDIs respectively. The remaining volume of solutions were made up with deionized water. Solutions were stirred overnight until all solids had dissolved. Multicomponent solutions were prepared either by powder or solution combination. Powder mixing refers to adding the two components together as solids and adding the appropriate total volume of aqueous NaOH (0.1 M). The remaining volume of solutions were made up with deionized water. Solutions were stirred overnight until all solids had dissolved. Solution mixing refers to each component being prepared as described for single component systems at a concentration of 10 mg mL^−1^. After stirring overnight, an equal volume of each of these solutions was added together and gently shaken to mix.

### Hydrogel Preparation

All hydrogels were prepared from solutions described as above using a pH trigger. 2 mL of the solution was pipetted into a 7 mL Sterilin vial that contained a pre‐weighed amount of glucono‐*δ*‐lactone (GdL) and shaken gently to dissolve the GdL. These then formed gels with a final pH of 3.8.

### UV‐Vis Absorption Measurements

Absorption spectra were collected using a Cary 60 UV‐visible spectrophotometer from Agilent Technologies. Solutions were measured in a 0.1 mm pathlength quartz cuvette (Hellma Analytics).

### Small Angle Neutron Scattering

SANS measurements were performed using the D11 instrument (Institut Laue Langevin, Grenoble, France). Solutions were prepared as previously described above using deuterated solvent and base. Gels were prepared as described above and transferred to 2 mm quartz cuvette immediately after the addition of GdL. The solutions were left to gel overnight before measurement. These were placed in a temperature‐controlled sample rack during the measurements. Data were reduced using Mantid software and data fitted using SASview.

### Rheology

Rheological tests were performed using an Anton Paar Physica 101 rheometer. Strain, frequency, and temperature data were collected using a vane (ST10‐4V‐8.8/97.5) geometry and a cup measuring system. Viscosity measurements were performed using an Anton Paar Physica 301 rheometer using a cone plate geometry (75 mm diameter, 1.0° angle, 50 µm).

## Conflict of Interest

The authors declare no conflict of interest.

## Supporting information

Supporting Information

## Data Availability

The data that support the findings of this study are available in the supplementary material of this article.
