# Supplementary material for: Process Dependent Complexity in Multicomponent Gels
Source: Macromol Rapid Commun. 2022 Oct 18;44(4):2200709. doi: 10.1002/marc.202200709 (PMC11475255; doi:10.1002/marc.202200709)
Supplement: Supplementary file 1 — Supporting Information [file MARC-44-2200709-s001.pdf]

Process dependent complexity in multicomponent gels

Rebecca I. Randle,[a] Rebecca E. Ginesi,[a] Olga Matsarskaia,[b] Ralf Schweins,[b] and Emily R. Draper*[a]

[a] School of Chemistry, Joseph Black Building, University of Glasgow, Glasgow, G12 8QQ, UK.

[b] Institut Laue-Langevin, Large Scale Structures Group, 71 Avenue des Martyrs, CS 20156, F-38042 Grenoble CEDEX 9, France

*Corresponding author email: Emily.Draper@glasgow.ac.uk

SUPPORTING INFORMATION

1. Synthetic Procedures ……………………………………………………….S2
2. Experimental Protocols and Procedures………………………………..S2
3. Supplementary Figures…………………………………………………...S7
4. References…………………………………………………………….…..S69

1. Synthetic Procedures

All chemicals and solvent were purchased from Merch Life Sciences, or Alfa Aesar and used as received with the exception of naphthalenetetracarboxylic dianhydride (NTCDA). NTCDA was purchased from Flurochem, Alfa Aesar and Merch Life Sciences but impurities were found by NMR in all supplier batches. Following this, all NTCDA was purchased from Fluorchem and purified. Deionised water was used throughout.

NDI-GF[1,2] and **NDI-F**[3–8] have been by our group[9] and others.[10,11] The synthesis of **1-NapFF** has been reported elsewhere.[13–15]

2. Experimental protocols and equipment

2.1. Freeze-dryer

Synthetic products were dried by lyophilisation. Solids were neutralised by stirring for 30 minutes in water and filtering. This was repeated until the filtrate was no longer acidic (determined using universal indicator paper (Merch Life Sciences)). Solids were then frozen using a freezer to approximately -18° C. Water was removed using a LSCbasic Freeze-dryer (Christ) at -85° C and between 0.890 and 1.25 mBar.

2.2. Nuclear Magnetic Resonance Spectroscopy (NMR)

For the characterisation after the synthesis of NDIs, NMR measurements were carried out on a Bruker 400 MHz spectrometer. Solids were dissolved in approximately DMSO-d_6. Approximately 2 µL trifluoroacetic acid was added in the case of **NDI-F** to move the position of the residual water signal in the ^{1}H NMR spectrum which covered signals from our products. Spectrometer operated at 400 MHz for ^{1}H NMR and 101 MHz for ^{13}C NMR spectroscopy.

2.3. Preparation of solutions

Single component solutions were prepared at concentrations 5 mg/mL. Solids were dissolved in one or two molar equivalents of aqueous NaOH (0.1 M) for **1-NapFF** and **NDI-R** respectively. The remaining volume of solutions were made up with deionised water. Solutions were stirred overnight until all solids had dissolved.

Multicomponent solutions were prepared either by powder or solution combination. Powder mixing refers to adding the two components together as solids and adding the appropriate total volume of aqueous NaOH (0.1 M). The remaining volume of

solutions were made up with deionised water. Solutions were stirred overnight until all solids had dissolved, more NaOH is added if required. Solution mixing refers to each component being prepared as described for single component systems at a concentration of 10 mg/mL. After stirring overnight, an equal volume of each of these solutions was added together and gently shaken to mix.

2.4. Preparation of Hydrogels

All hydrogels were prepared from solutions described as above using a pH trigger. 2 mL of the solution was adjusted to pH 11 and transferred to a 7 mL Sterilin vial that contained a pre-weighed amount of glucono-δ-lactone (GdL) and shaken gently to dissolve the GdL. Due to the differences in components and overall solution concentration, the exact mass of GdL are tabulated in Table S1.

Table S1. Concentration of glucono-δ-lactone required to form hydrogels from each single and multicomponent system from pH 11 to a gel with final pH of approximately 3.8

Mix	GdL concentration (mg/mL)	Average pH
**1-NapFF** 5 mg/mL	4.5	3.9
**NDI-GF** 5 mg/mL	5	3.9
**S*** 5:5 mg/mL	10	3.9
**S** 5:5 mg/mL	9	3.8
**P** 5:5 mg/mL	9	3.8
**S*** 5:5 mg/mL	10	3.8
**P*** 5:5 mg/mL	10.5	3.8

Each gel was left stand undisturbed overnight after the addition of GdL. All preparation of samples was done at room temperature. The next morning, if samples

were stable to inversion, rheological measurements were taken. After measurement, pH of the destroyed gel was taken. Measurements were performed in triplicate.

2.5. pH measurements

pH was measured using a FC200 pH probe (HANNA instruments) with a 6 mm × 10 mm conical tip calibrated with buffers of pH 4, 7 and 10 (HANNA instruments). The stated accuracy of the pH measurements is ±0.1.

2.6. Apparent pK_a Titrations

All pK_a titrations were performed using solutions prepared as described above adjusted to pH 12. 0.1 M HCl was added in 5-10 µL portions. A portion was added, and the solution gently stirred before being allowed to sit for 5 minutes before a pH measurement was taken and another portion added. The temperature was maintained at 25°C during the titration by using a circulating water bath. pK_a values were determined to be the pH at which a plateau is observed upon addition of HCl before the pH dropping again more steadily.

2.7. Absorption Spectroscopy

Absorption spectra were collected using a Cary 60 UV-Visible spectrophotometer from Agilent Technologies. Solutions were measured in a 0.1 mm pathlength quartz cuvette (Hellma Analytics). Spectra were collected from 250-1100 nm at a scan rate of 2 nm/s unless stated otherwise. Spectra are background/zero calibrated using distilled water as a blank background.

2.7.1.1. Spectra of Gels

Solutions were measured in a 0.1 mm pathlength quartz cuvette (Hellma Analytics). 1 mL of solution was added to GdL as previously described. The solution was gently shaken and then poured into the cuvette so as not to disturb any aggregates forming using shear of a pipette. The cuvette was sealed using parafilm to prevent the gel drying out and placed in a dark box covered in foil overnight (to prevent photo-reduction of NDI components within the gels). The following day, the parafilm was removed and the gel was measured. Spectra were collected from 250-1100 nm at a scan rate of 2 nm/s unless stated otherwise. Spectra are background/zero calibrated using distilled water as a blank background.

2.8. Rheological Measurements

2.8.1. Strain Measurements

Dynamic rheological experiments were performed using an Anton Paar Physica 101 rheometer. Strain and frequency data were collected using a vane (ST10-4V-8.8/97.5) geometry and a cup measuring system. 7 mL Sterlin vials fit into this measuring system plate and were secured with blue tack if needed. Measurements were recorded in triplicate. Strain sweeps were recorded from 0.1-1000% strain at set frequency of 10 rad/s with a gap height of 1.8 mm.

2.8.2. Frequency Measurements

Strain and frequency data were collected using a vane (ST10-4V-8.8/97.5) geometry and a cup measuring system. Frequency scans were recorded from 1 rad/s to 100 rad/s under a constant strain of 0.5% with a gap height of 1.8 mm. This strain was chosen because it is in the linear viscoelastic region for the hydrogels. Samples were prepared as described previously in a 7 mL Sterlin vial. Measurements were performed in triplicate.

2.8.3. Viscosity Measurements

All viscosity measurements were performed using an Anton Paar Physica 301 rheometer using a cone plate geometry (75 mm diameter, 1.0° angle, 50 µm) and a parallel plate measuring system for all measurements. A gap of 0.05 mm was used. Measurements were recorded in triplicate. 3 mL of solution (prepared and pH adjusted as previously described) was deposited onto the plate and trimmed to the geometry. All experiments were performed at 25°C maintained using a water bath. Measurements were recorded at a shear rate from 0.1-1000%

2.8.4. Temperature Measurements

Temperature sweep measurements were performed using an Anton Paar Physica 301 rheometer. Data was collected using a vane (ST10-4V-8.8/97.5) geometry and a cup measuring system. 2 mL gels were prepared as described above in aluminium rheology cups. Temperature sweep measurements were performed under a constant strain of 0.5% and frequency of 10 rad/s with a gap height of 1 mm. Measurements were run between 25°C and 90°C at a heating rate of 0.5°C/min.

2.9. Small Angle Neutron Scattering (SANS)

Solutions were prepared as previously described above using deuterated solvent and base. pD was adjusted using 0.1M NaOD and DCl. Solutions were adjusted to pD 11 and gels were prepared as described above and transferred to cuvette immediately after addition of GdL. The solution was left to gel overnight before measurement. Multicomponent solutions were prepared as previously described above.

The measurements were performed using solution deposited into quartz cuvettes (Hellma) with a 2 mm path length. These were placed in a temperature-controlled sample rack during the measurements. SANS measurements were performed using the D11 instrument (Institut Laue Langevin, Grenoble, France). A neutron beam, with a fixed wavelength of 6 Å and a divergence of $\Delta\lambda/\lambda$ = 9%, allowed measurements over a large range in Q [$Q = 4\pi \sin(\theta/2)/\lambda$] range of 0.001 to 0.3 $Å^{-1}$, by using three sample-detector distances of 2 m (Coll 4 m), 8 m (Coll 8 m) and 28 m (Coll 28 m). Measurements took ~60 minutes. The data were reduced to 1D scattering curves of intensity vs. Q using Mantid. The electronic background was subtracted, the full detector images for all data were normalized and scattering from the empty cell was subtracted. The scattering from D_2O was also measured and subtracted from the data. Most of the data were radially averaged to produce the 1D curves for each detector position. The instrument-independent data were then fitted to the models discussed in the text using Sasview, Version 4.2.2.[18] Fits were chosen based on how well the model fitted the data and which model gave the lowest value of Chi^2. Fitting parameters are given in Tables corresponding to figures as made clear in captions within the Supporting Information, Section 3. Data can be found at doi.10.5291/ILL-DATA.9-10-1670. SLD solvent was calculated to be $6.39 \times 10^{-6}/Å^2$. SLD of **NDI-F, NDI-GF** and **1-NapFF** were calculated to be 3.246, 3.174 and 2.678 $\times 10^{-6}/Å^2$ respectively. 5:5 mixtures of **1-NapFF:NDI-GF** and **1-NapFF:NDI-F** were calculated to have an SLD of 2.962 and 2.926 $\times 10^{-6}/Å^2$ respectively. These values were an average of the SLD of each component added together.

3. Supplementary Figures

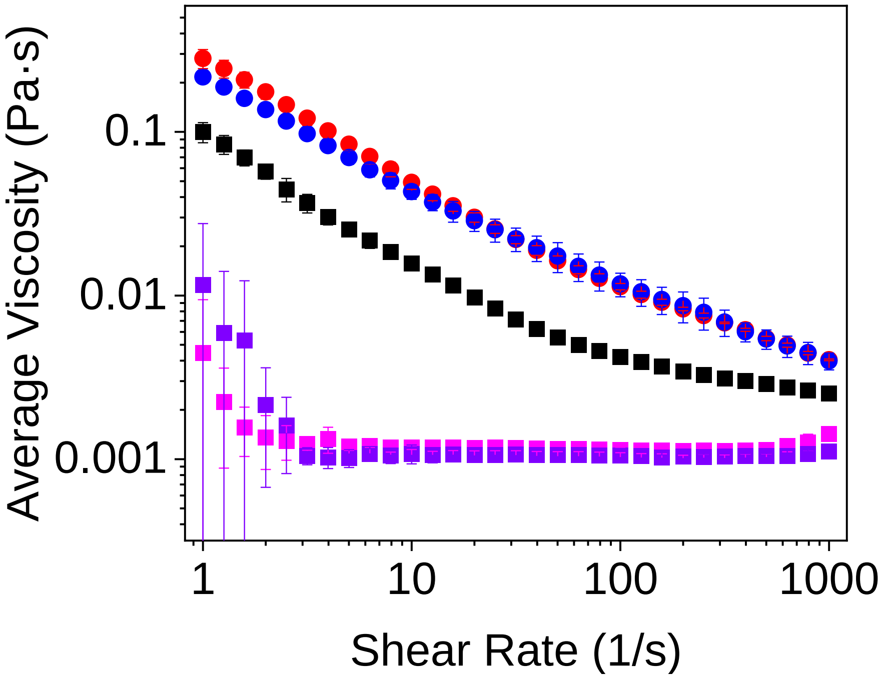

Figure S1. Viscosity measurements of **1-NapFF** (■), **NDI-F** (■)**, NDI-GF** (■) solutions at 5 mg/mL and **S*** (•) and **S** (•) solutions at 5:5 mg/mL. Solutions adjusted to pH 11. Error bars calculated from the standard deviation of three measurements.

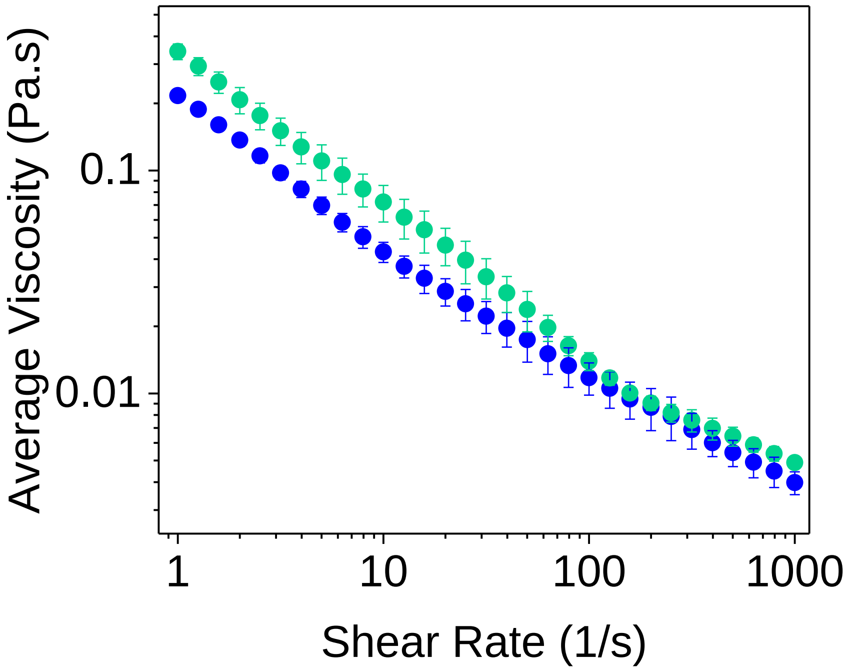

Figure S2. Viscosity measurements of **S** (•) and **P** (•) at 5:5 mg/mL. Solutions adjusted to pH 11. Error bars calculated from the standard deviation of three measurements.

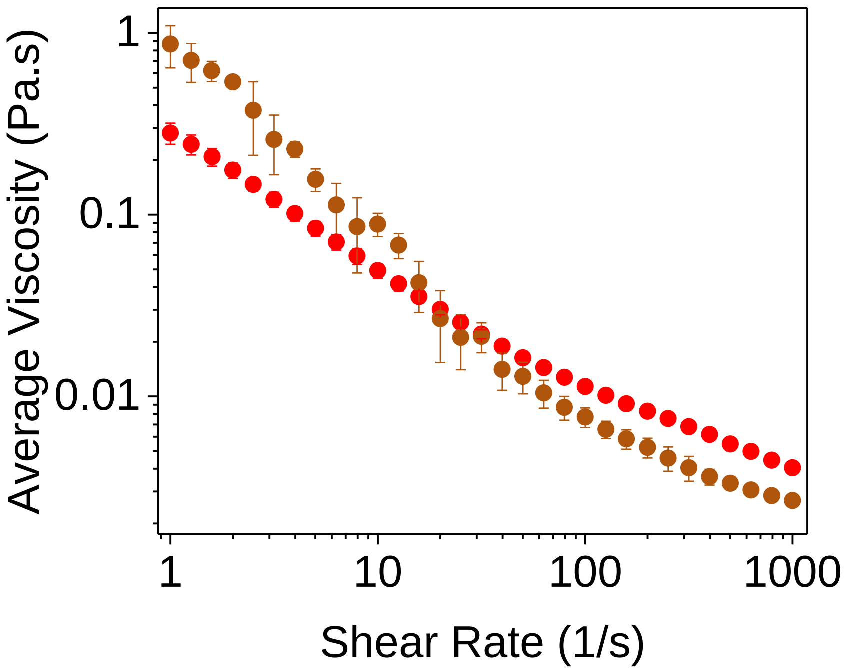

5

Figure S3. Viscosity measurements of **S*** (•) and **P*** (•) at 5:5 mg/mL. Solutions adjusted to pH 11. Error bars calculated from the standard deviation of three measurements.

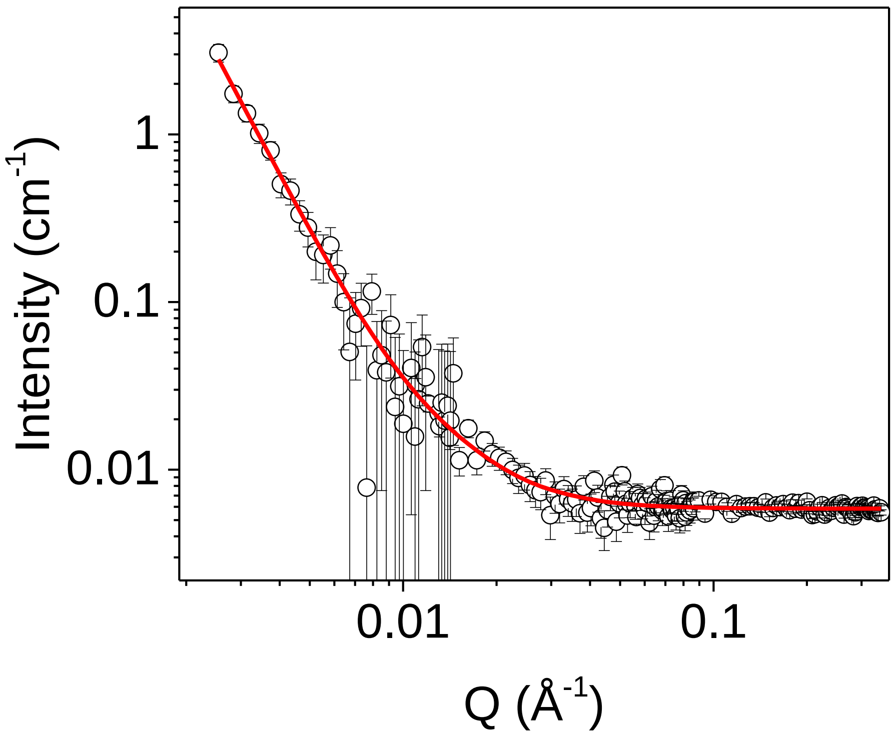

Figure S4. Small angle neutron scattering data of **NDI-GF** at pD 11 (O) fitted to a flexible elliptical cylinder model combined with power law (–).

Table S2. Parameters of SANS model fit above.

**NDI-GF** pD 11	Flexible elliptical cylinder and power law	
	**Value**	**Error**
Background (cm^{-1})	0.005854	3.04E-05
Scale A	3.55E-07	2.08E-07
Axis Ratio	30.136	3.820
Radius (Å)	4.9891	0.637
Kuhn Length (Å)	41.742	197
Length (Å)	399.76	263.6
Scale B	1.99E-09	8.38E-10
Power	3.53	7.73E-02
Range	0.00254305-0.346809	
Chi^2	0.8631	

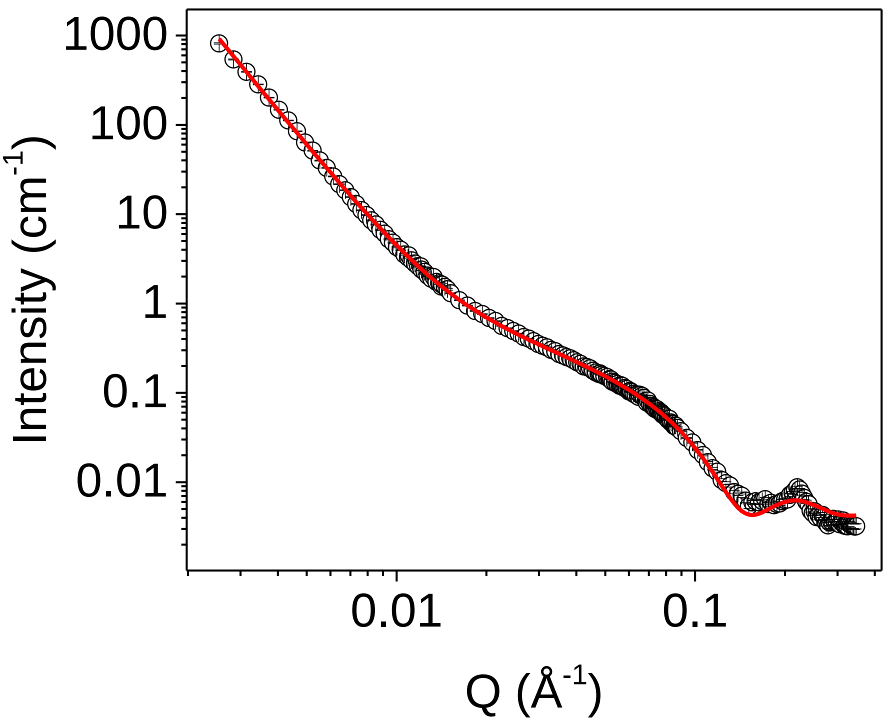

Figure S5. Small angle neutron scattering data of **1-NapFF** at pD 11 (O) fitted to a hollow cylinder model combined with power law (–).

Table S3. Parameters of SANS model fit above.

**1-NapFF** pD 11	Hollow cylinder and power law	
	**Value**	**Error**
Background (cm^{-1})	0.004224	3.24E-05
Scale A	0.001572	2.02E-06
Thickness (Å)	16.675	0.019508
Radius (Å)	6.2412	0.025605
Length (Å)	3943.9	52.503
Scale B	2.51E-08	4.89E-11
Power	4.0704	0.00038379
Range	0.00254305-0.346809	
Chi^2	10.535	

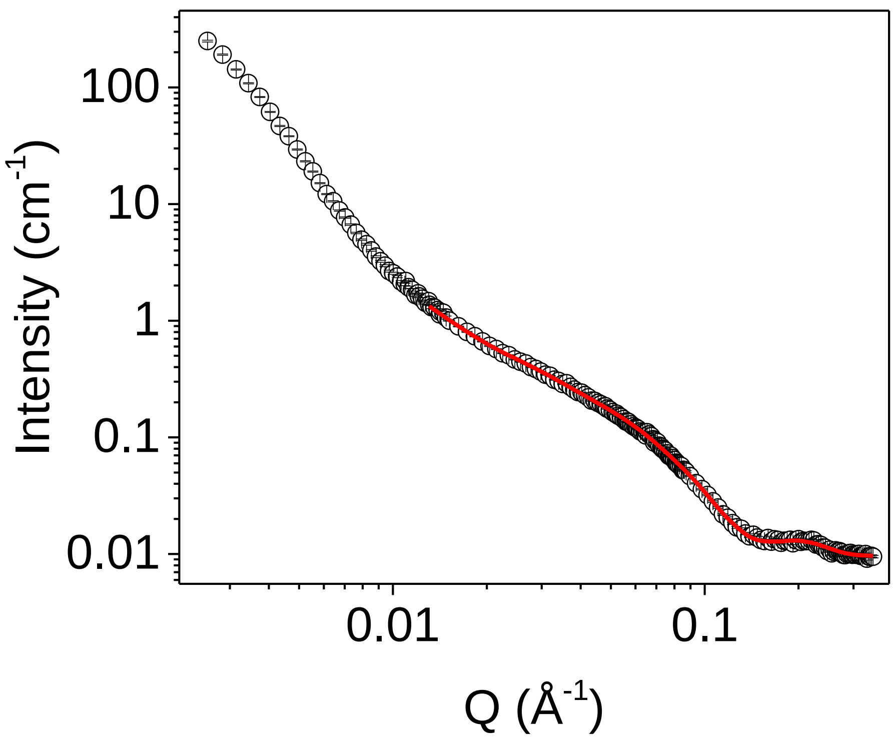

Figure S6. Small angle neutron scattering data of **S** at pD 11 (O) fitted to a hollow cylinder model combined a flexible elliptical cylinder model (–).

Table S4. Parameters of SANS model fit above.

**S** pD 11	Hollow cylinder (HC) and flexible elliptical cylinder (FEC)	
	**Value**	**Error**
Background (cm^{-1})	0.00928	3.35E-05
Scale A (HC)	0.001544	2.42E-06
Thickness HC (Å)	17.318	0.024931
Radius HC (Å)	6.6699	0.032326
Length HC (Å)	183.52	1.7578
Scale B (FEC)	3.91E-05	1.49E-07
Axis Ratio (FEC)	36.892	0.15328
Radius FEC (Å)	4.9992	0.021707
Kuhn Length FEC (Å)	156.59	1.403
Length FEC (Å)	2.46E+11	2.66E+11
Range	0.01305-0.346809	
Chi^2	2.3803	

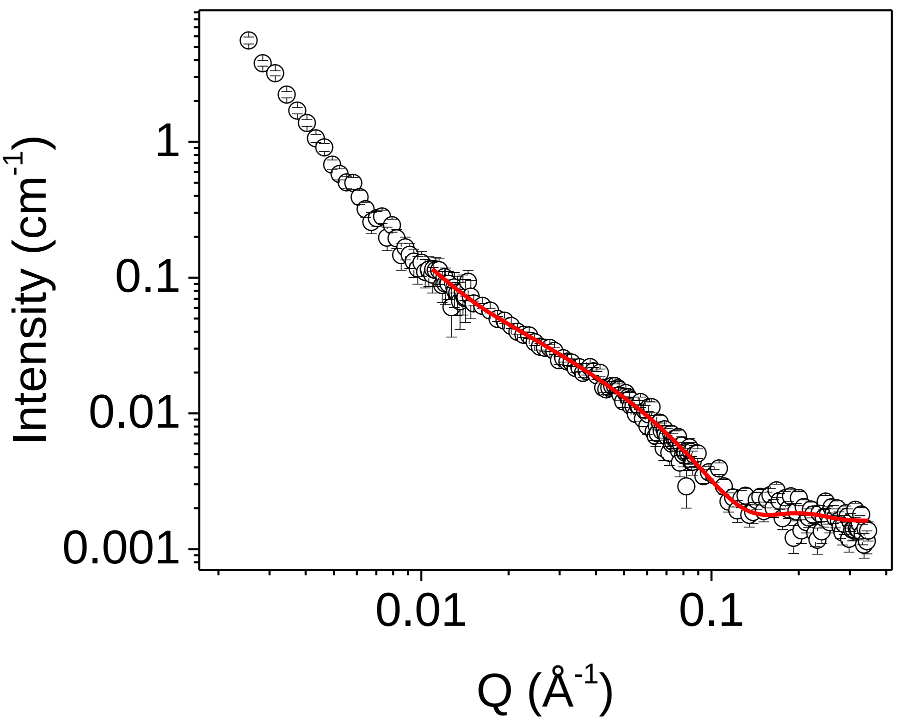

Figure S7. Small angle neutron scattering data of **P** at pD 11 (O) fitted to a flexible elliptical cylinder model combined with a power law (–).

Table S5. Parameters of SANS model fit above.

**P** pD 11	Flexible elliptical cylinder (FEC) and power law (PL)	
	**Value**	**Error**
Background (cm^{-1})	0.001701	3.17E-05
Scale A (FEC)	7.99E-05	6.69E-07
Axis Ratio	1.5125	7.21E-03
Radius (Å)	20.022	0.10121
Kuhn Length (Å)	353.24	20.482
Length (Å)	1.30E+03	5.70E-02
Scale B (PL)	5.64E-10	1.16E-11
Power	3.8626	3.66E-03
Range	0.00254305-0.346809	
Chi^2	1.2747	

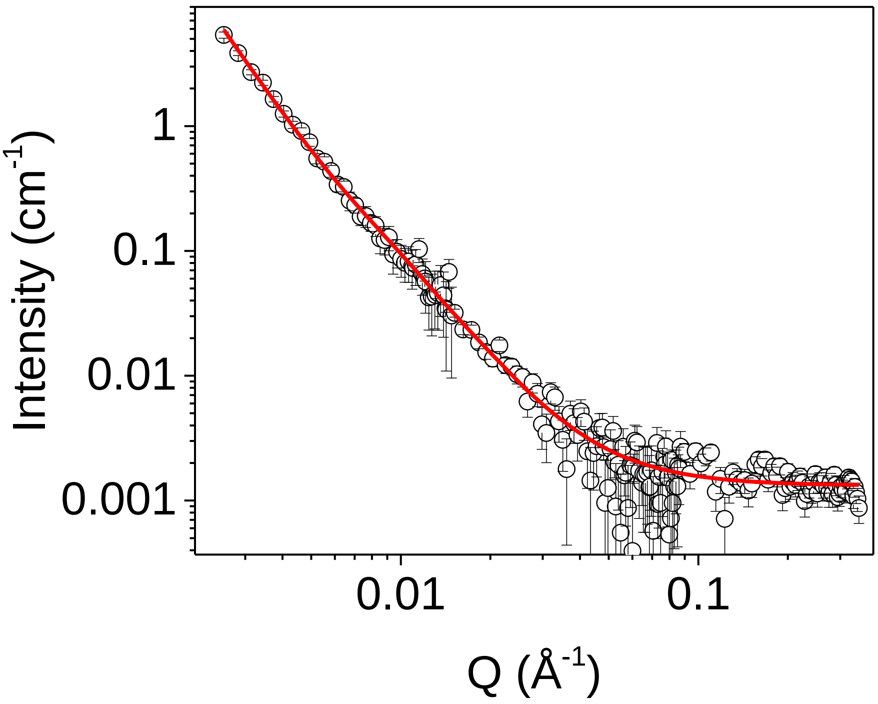

Figure S8. Small angle neutron scattering data of **NDI-F** at pD 11 (O) fitted to a flexible elliptical cylinder model combined with power law (–).

Table S6. Parameters of SANS model fit above.

**NDI-F** pD 11	Flexible elliptical cylinder (FEC) and power law (PL)	
	**Value**	**Error**
Background (cm^{-1})	0.001327	3.16E-05
Scale A (FEC)	8.63E-07	3.14E-08
Axis Ratio	50.024	1.760
Radius (Å)	5.1853	0.184
Kuhn Length (Å)	94.454	10.528
Length (Å)	738.47	78.856
Scale B (PL)	4.60E-09	8.77E-11
Power	3.51	3.081E-3
Range	0.00254305-0.346809	
Chi^2	0.95629	

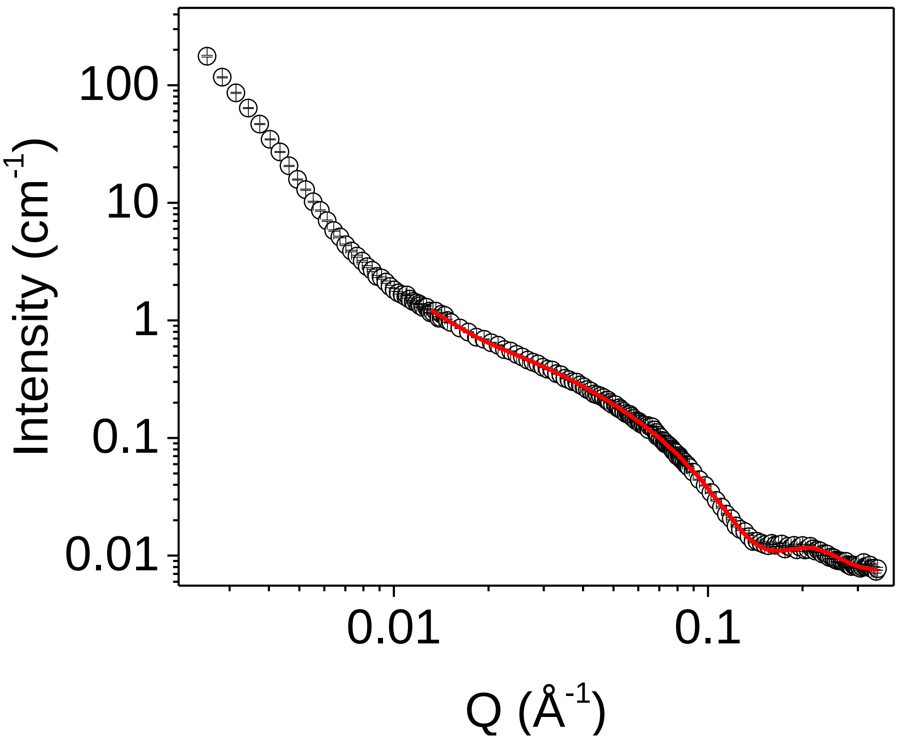

Figure S9. Small angle neutron scattering data of **S*** at pD 11 (O) fitted to a hollow cylinder model combined a flexible elliptical cylinder model (–).

Table S7. Parameters of SANS model fit above.

**S*** pD 11	Hollow cylinder (HC) and flexible elliptical cylinder (FEC)	
	**Value**	**Error**
Background (cm^{-1})	0.007226	3.34E-05
Scale A (HC)	0.001975	2.71E-06
Thickness HC (Å)	15.269	0.018793
Radius HC (Å)	7.3246	0.025427
Length HC (Å)	7468.6	47.498
Scale B (FEC)	5.64E-05	2.36E-07
Axis Ratio FEC	32.022	0.1391
Radius FEC (Å)	4.9852	0.023117
Kuhn Length FEC (Å)	174.07	1.6355
Length FEC (Å)	1.48E+77	3.27E+70
Range	0.01305-0.346809	
Chi^2	4.4443	

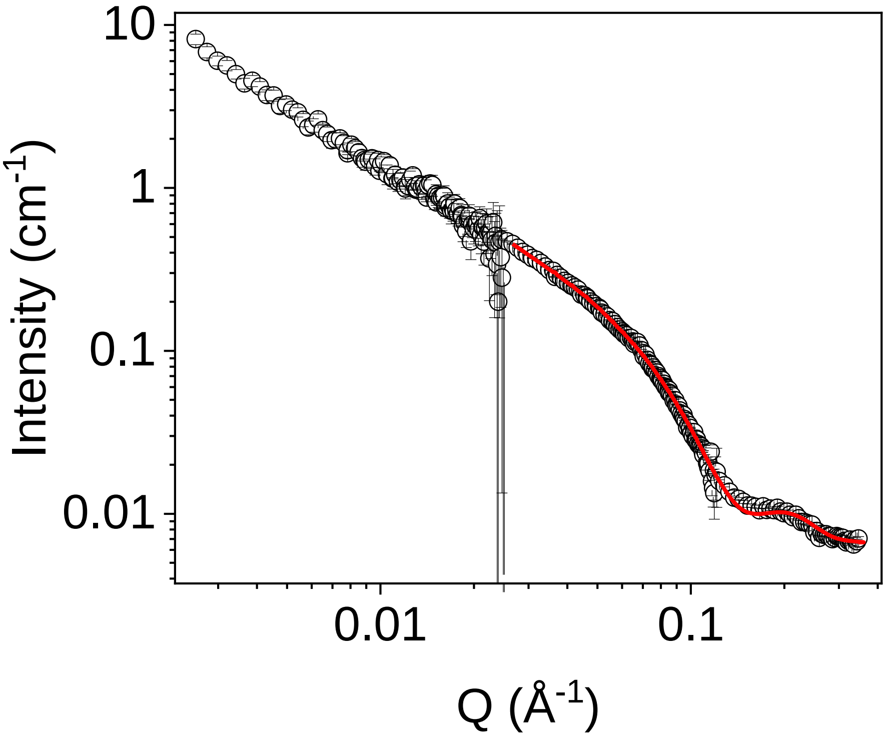

Figure S10. Small angle neutron scattering data of **P*** at pD 11 (O) fitted to a hollow cylinder model combined a flexible elliptical cylinder model (–).

Table S8. Parameters of SANS model fit above.

**P*** pD 11	Hollow cylinder (HC) and flexible elliptical cylinder (FEC)	
	**Value**	**Error**
Background (cm^{-1})	0.006338	2.23E-05
Scale A (HC)	0.001769	2.07E-06
Thickness HC (Å)	17.649	0.01731
Radius HC (Å)	6.3764	0.024968
Length HC (Å)	239.5	4.5355
Scale B (FEC)	4.03E-05	1.68E-07
Axis Ratio FEC	38.353	0.16373
Radius FEC (Å)	4.7483	0.021531
Kuhn Length FEC (Å)	170.06	3.5416
Length FEC (Å)	1.11E+35	4.56E+30
Range	0.0266373-0.34681	
Chi^2	3.5596	

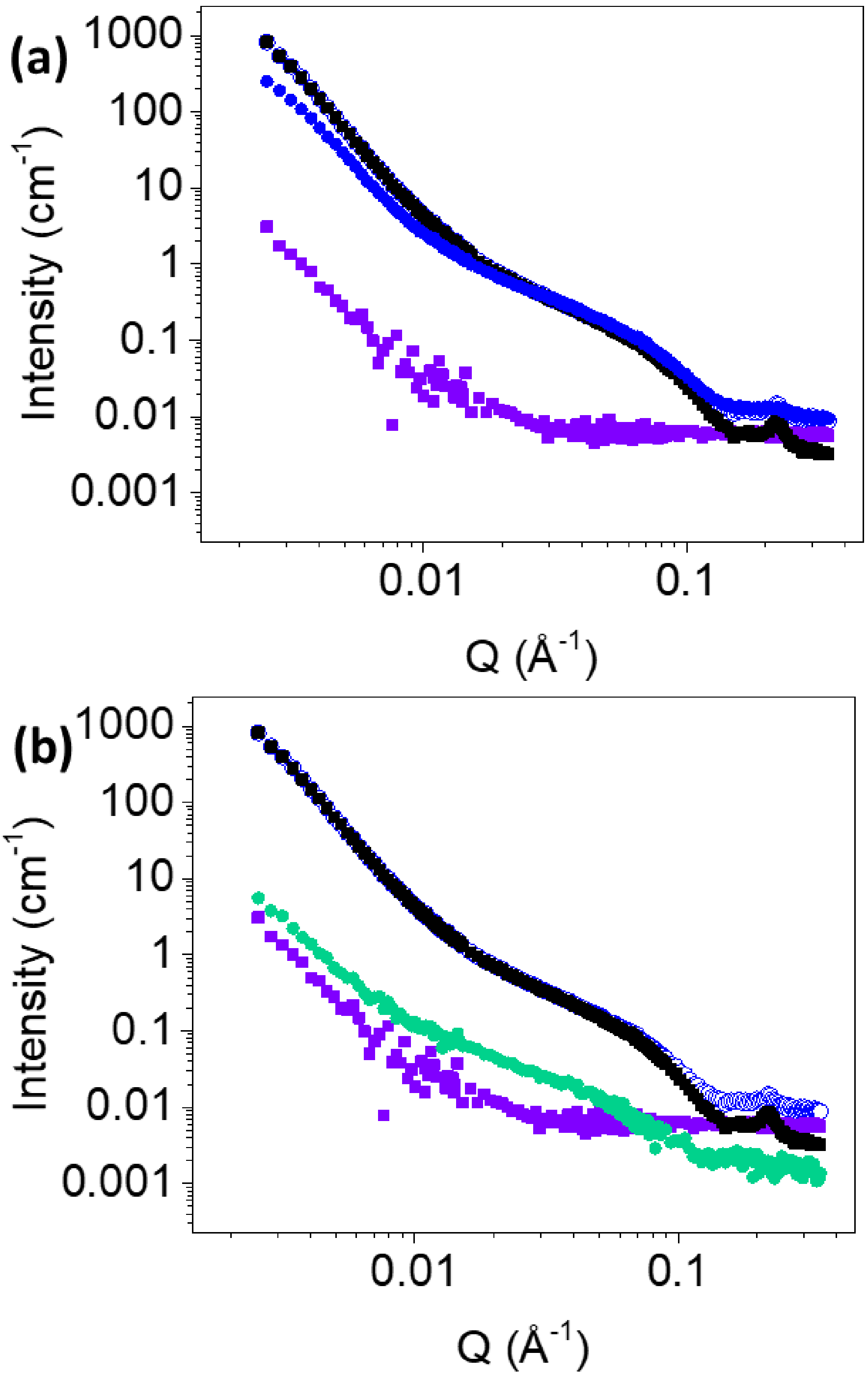

Figure S11. Small angle neutron scattering data from **1-NapFF** (■), **NDI-GF** (■) and (a) **S** (●) and (b) **P** (●) at pD 11. Simulated scattering data created by a simple addition of raw data of each component is shown for **S/P** (O).

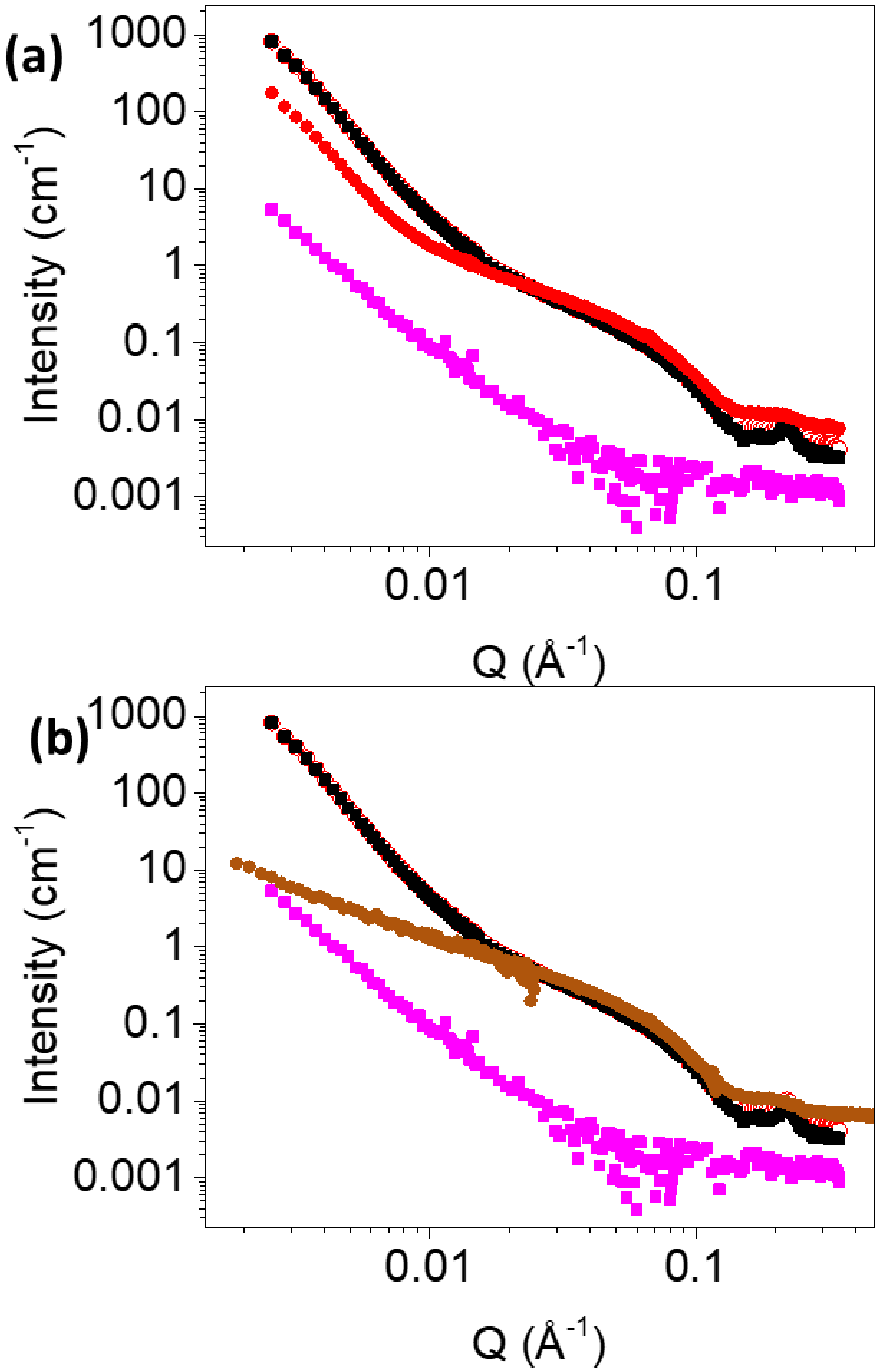

Figure S12. Small angle neutron scattering data from **1-NapFF** (■), **NDI-F** (■), (a) **S*** (●) and (b) **P*** (●) at pD 11. Simulated scattering data created by a simple addition of raw data of each component is shown for **S*/P** (O)

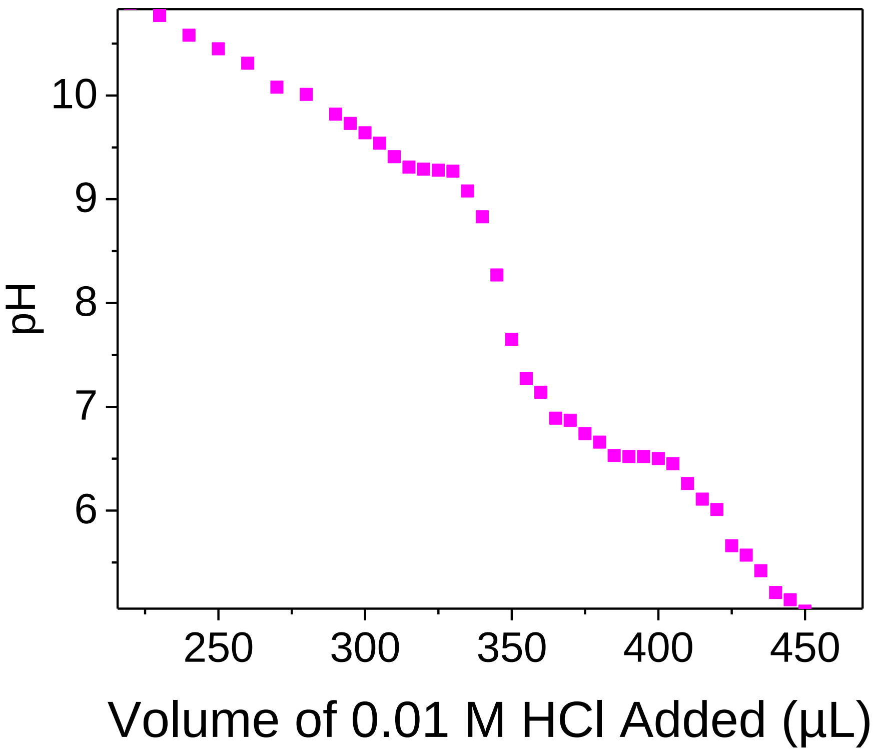

Figure S13. pH titration data taken for **NDI-F** at 5 mg/mL

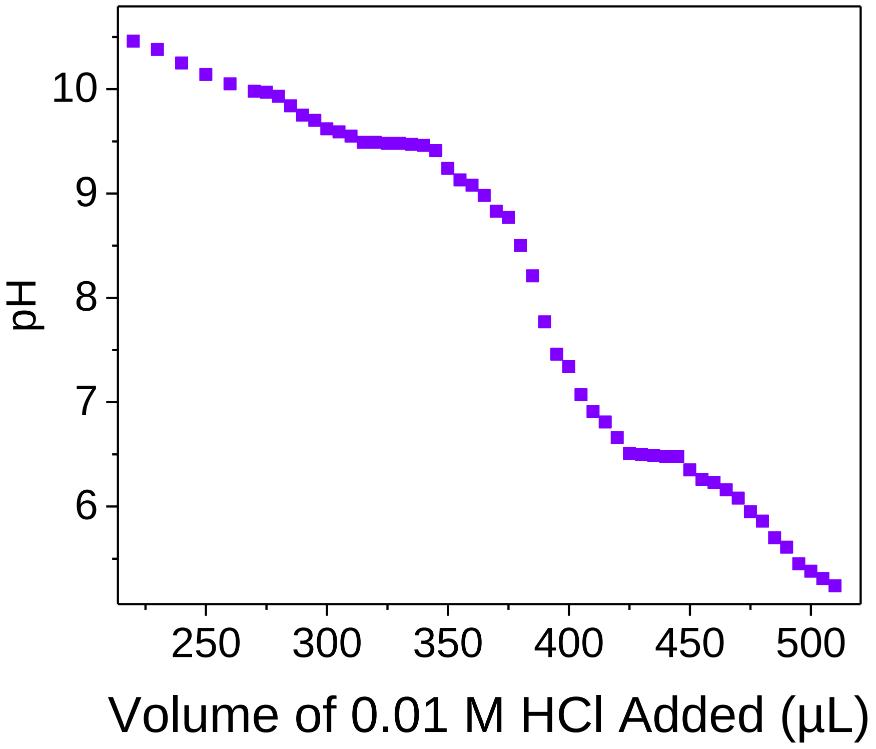

Figure S14. pH titration data taken for **NDI-GF** at 5 mg/mL

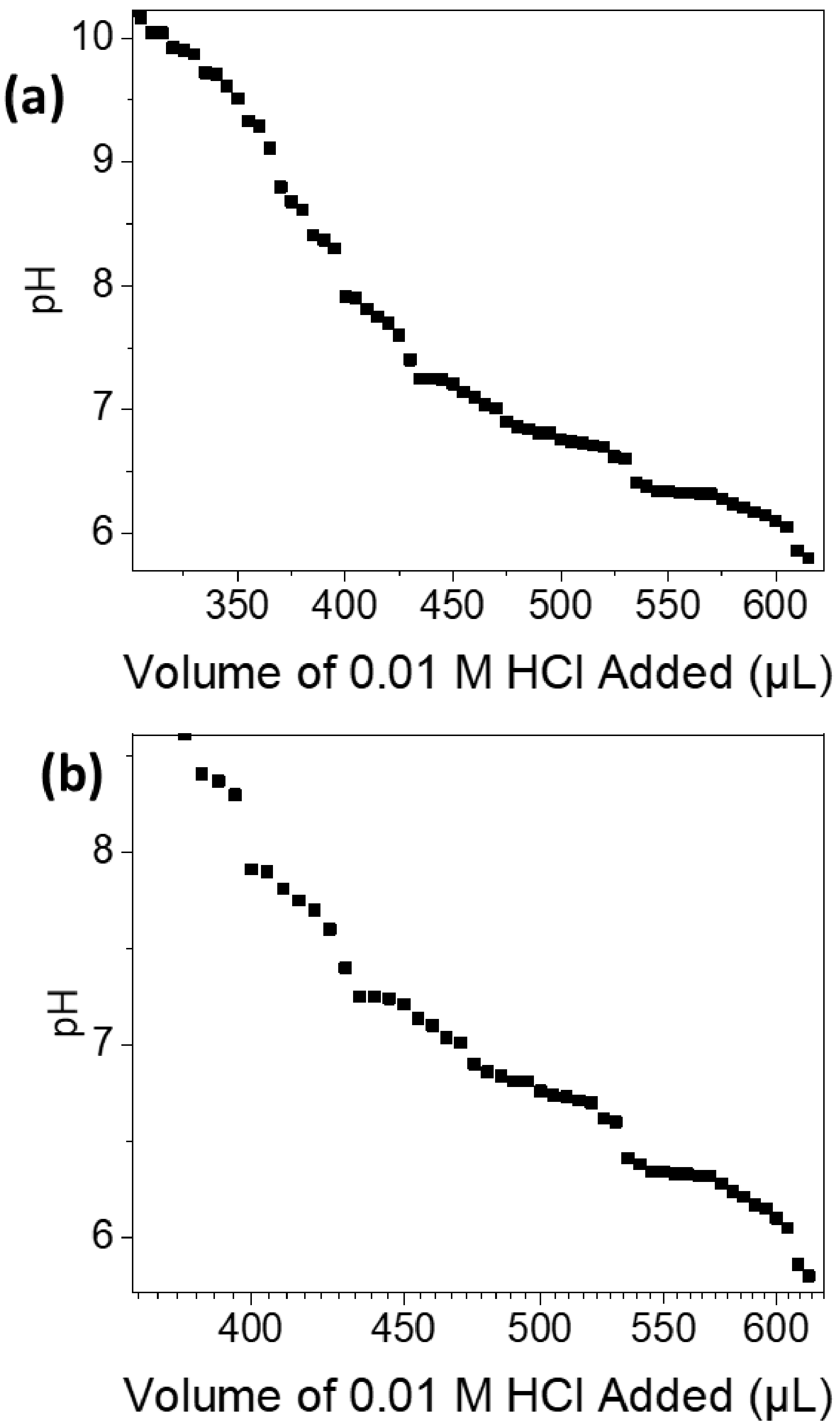

Figure S15. pH titration data taken for **1-NapFF** (a). The area of the plot with both pK_a values with x axis on a logarithmic scale is also shown (b).

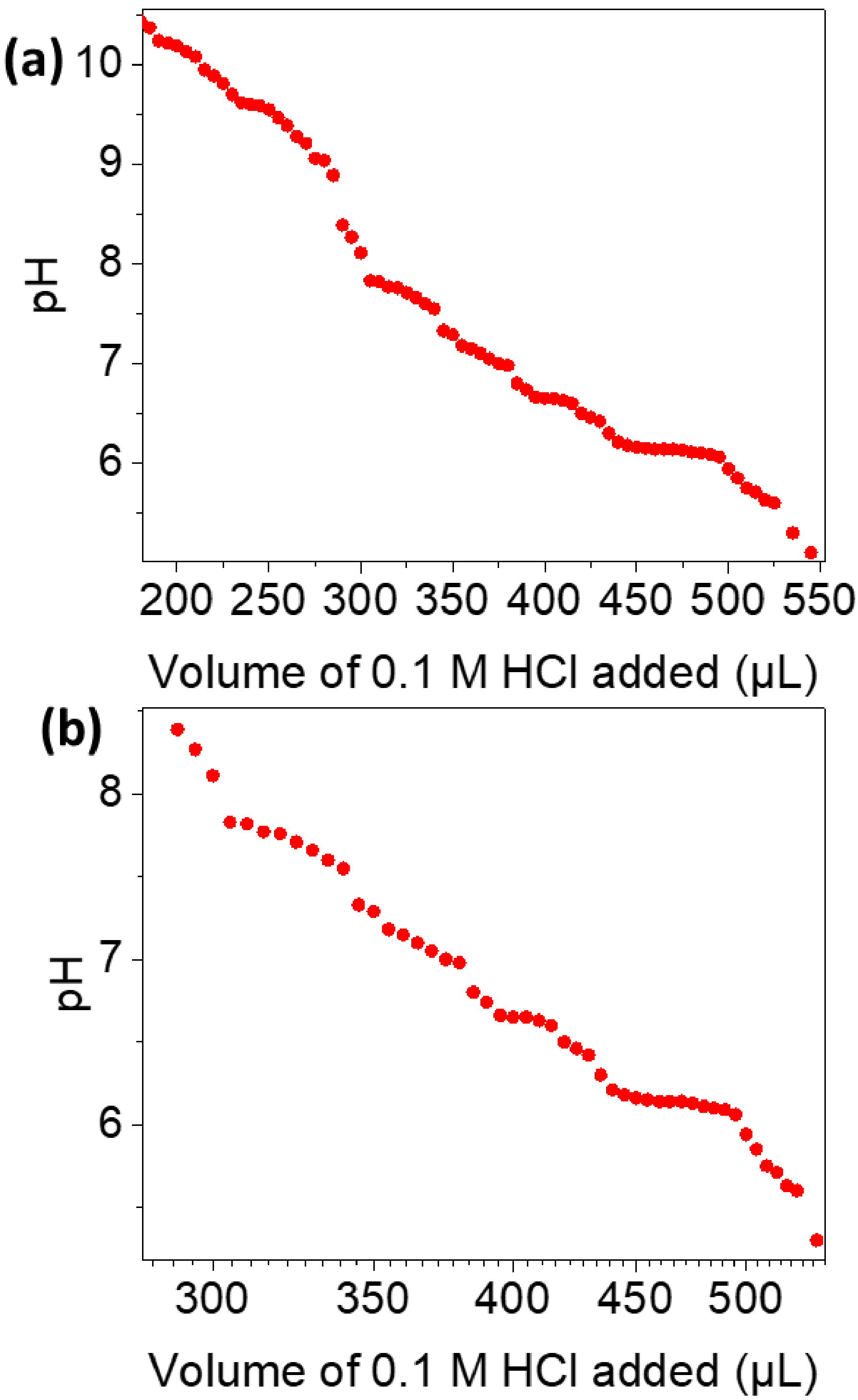

Figure S16. pH titration data taken for **S*** (a). The area of the plot with both pK_a values with x axis on a logarithmic scale is also shown (b).

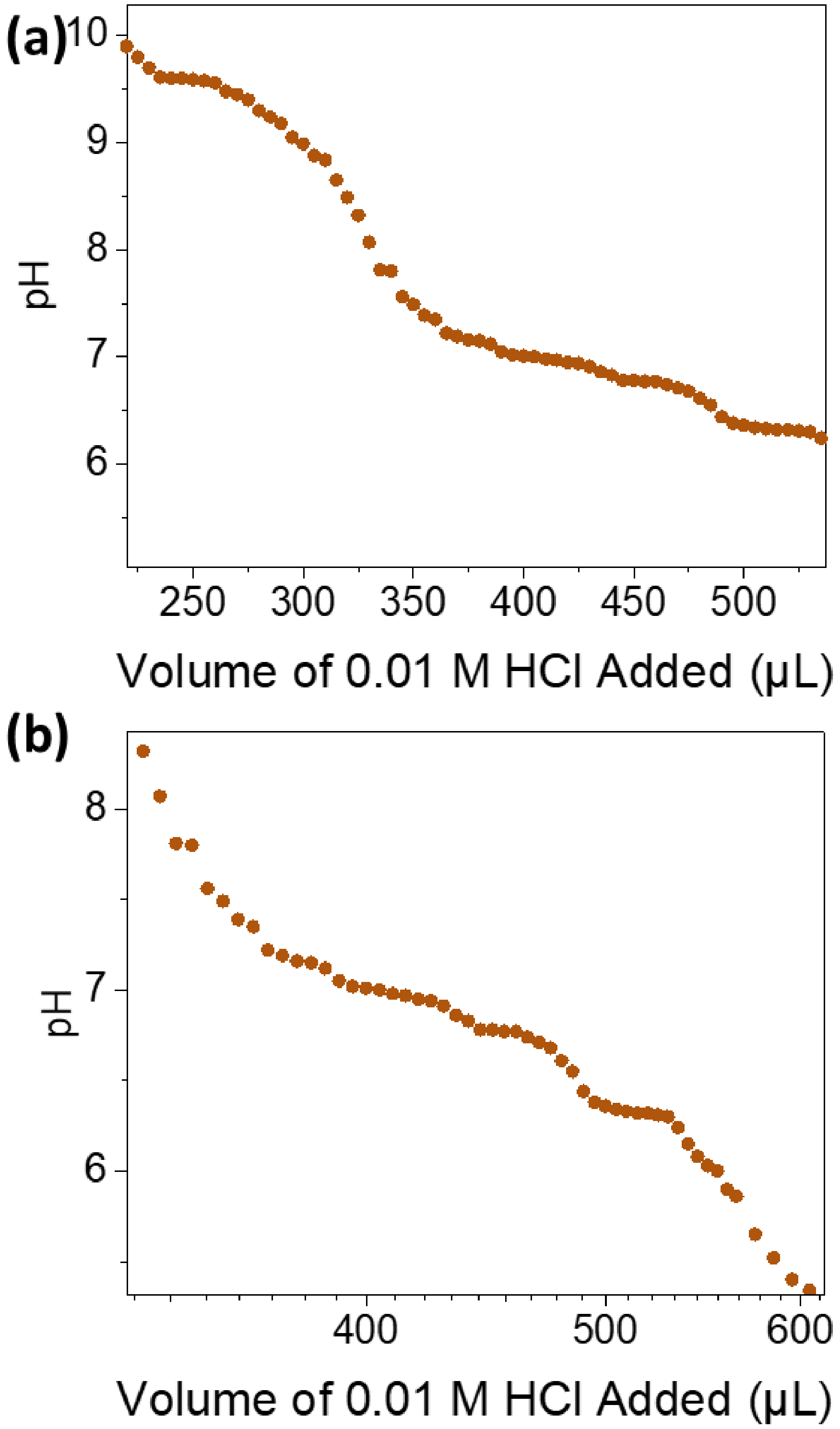

Figure S17. pH titration data taken for **P*** (a). The area of the plot with both pK_a values with x axis on a logarithmic scale is also shown (b).

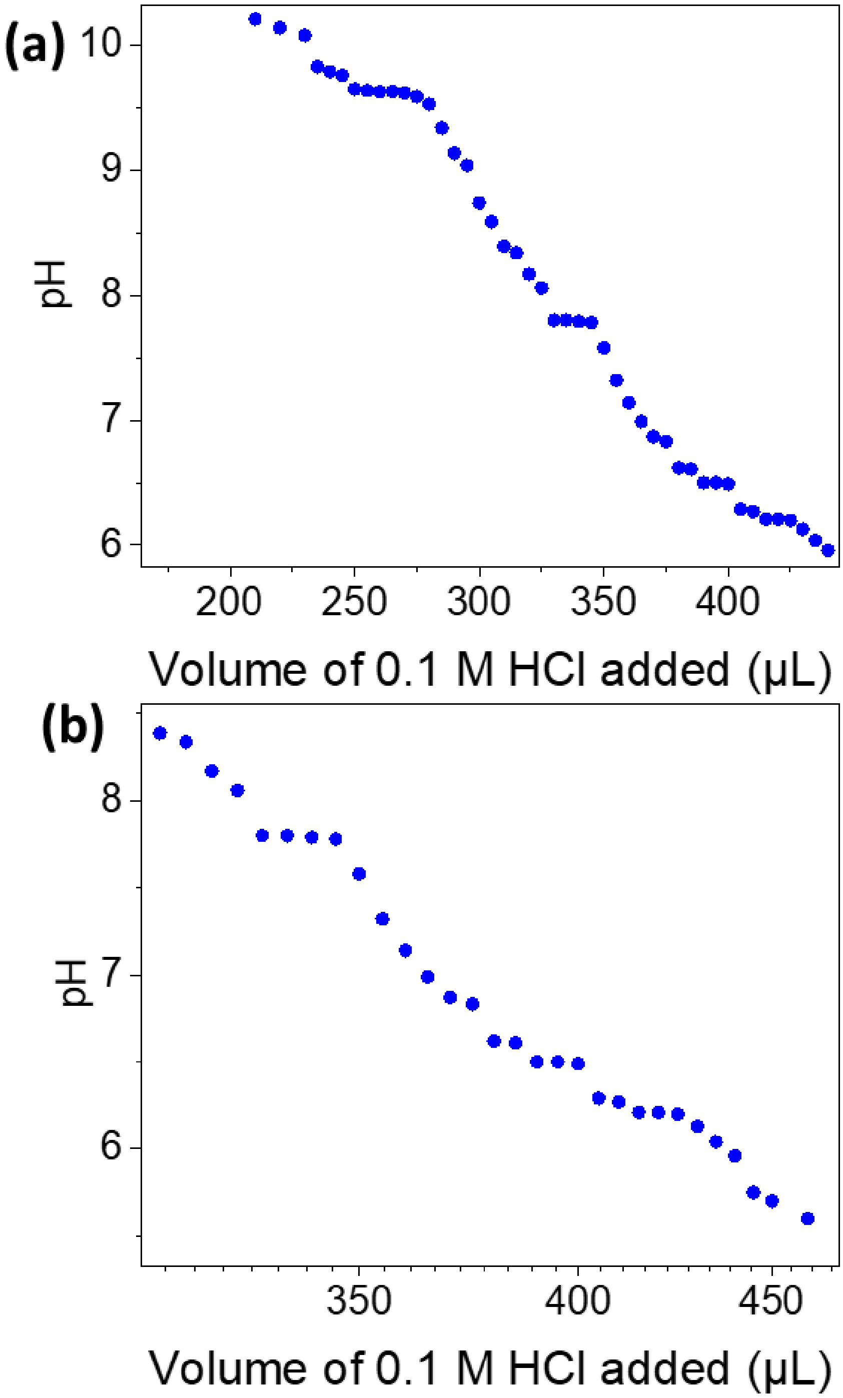

Figure S18. pH titration data taken for **S** (a). The area of the plot with both pK_a values with x axis on a logarithmic scale is also shown (b).

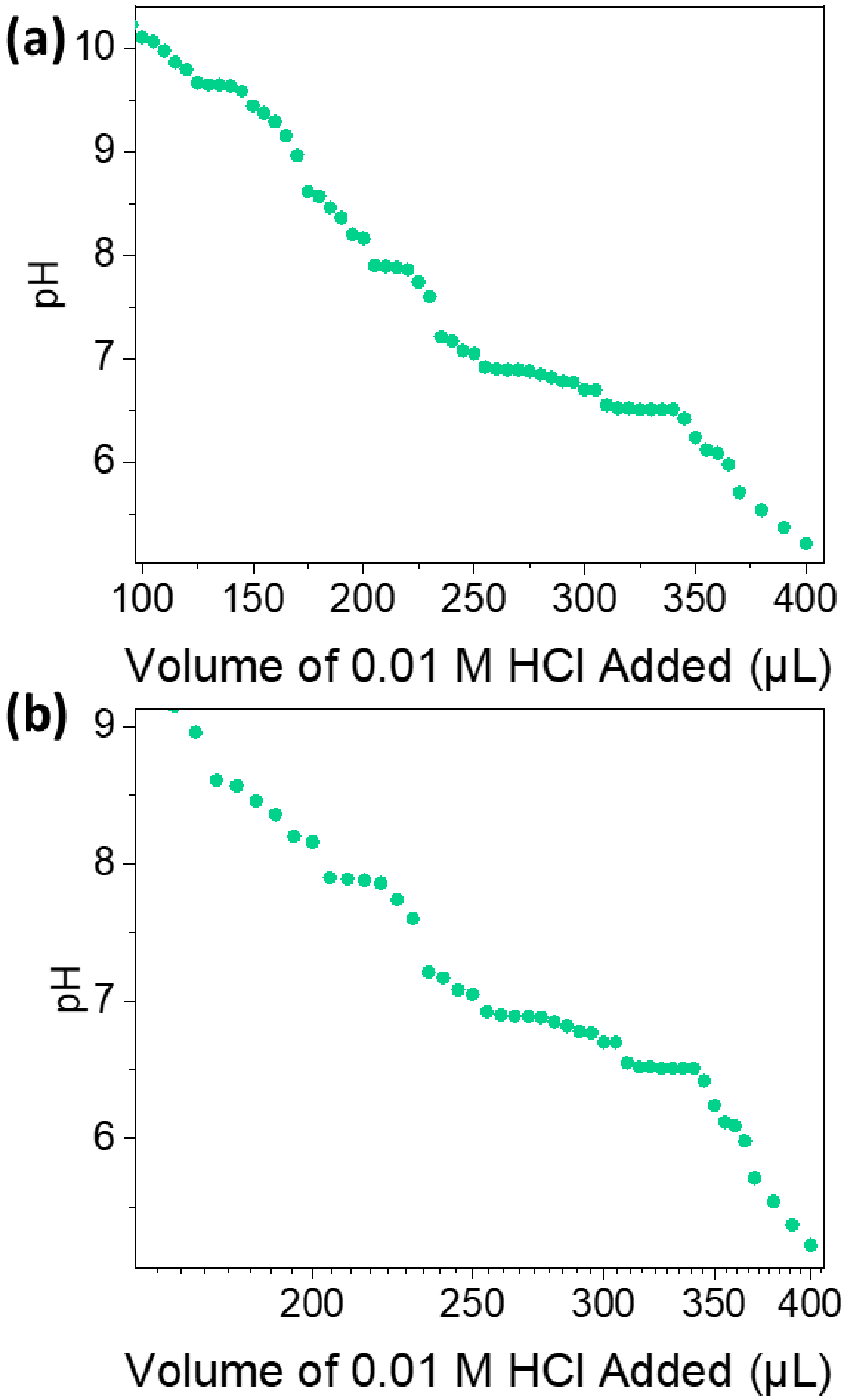

Figure S19. pH titration data taken for **P** (a). The area of the plot with both pK_a values with x axis on a logarithmic scale is also shown (b).

Table S9. Tabulated apparent pK_as of single and multicomponent solutions

NDI-F	1-NapFF	NDI-GF	S*	P*	S	P
9.3		9.5	9.6	9.6	9.6	9.6
	7.3		7.8	7.1	7.8	7.9
6.5		6.5	6.7	6.8	6.5	6.9
	6.3		6.1	6.3	6.2	6.5

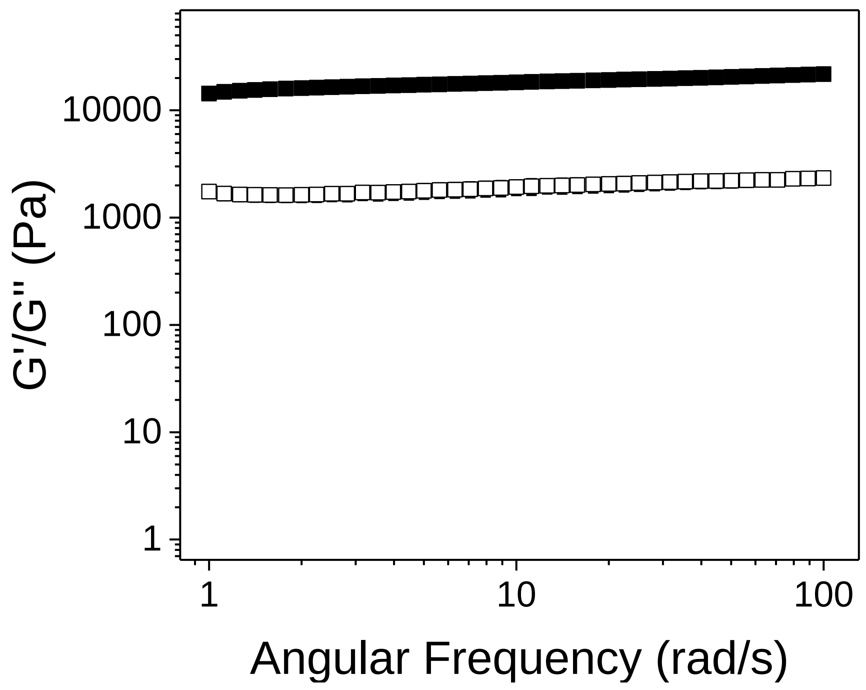

Figure S20. Frequency sweeps of **1-NapFF** gel at 5 mg/mL. Strain of 0.5 % is maintained. Error bars calculated from the standard deviation of three measurements. G’ are the solid shapes and G″ are the empty shapes.

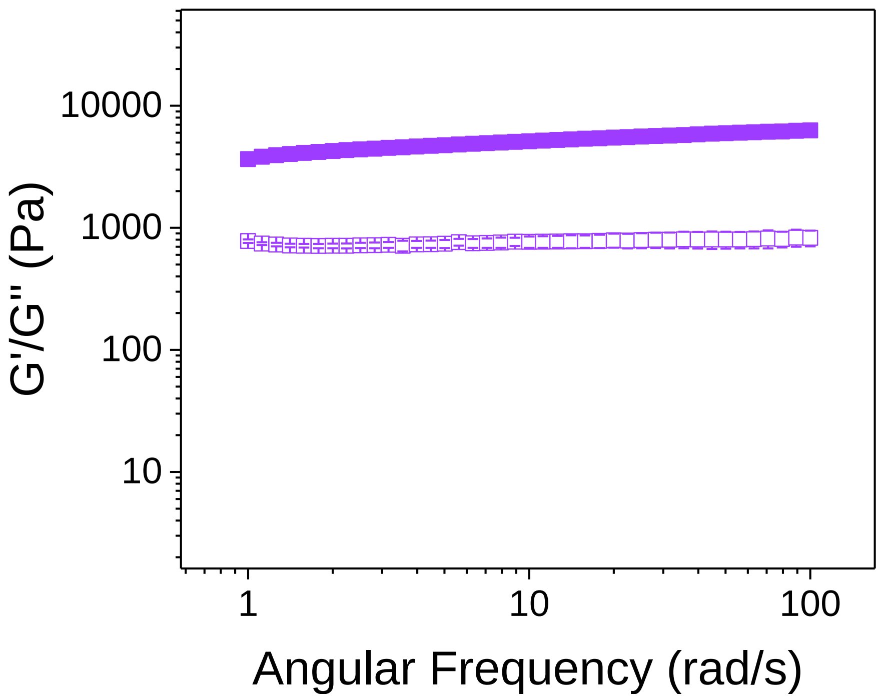

Figure S21. Frequency sweeps of **NDI-GF** gel at 5 mg/mL**.** Strain of 0.5 % is maintained. Error bars calculated from the standard deviation of three measurements. G’ are the solid shapes and G″ are the empty shapes.

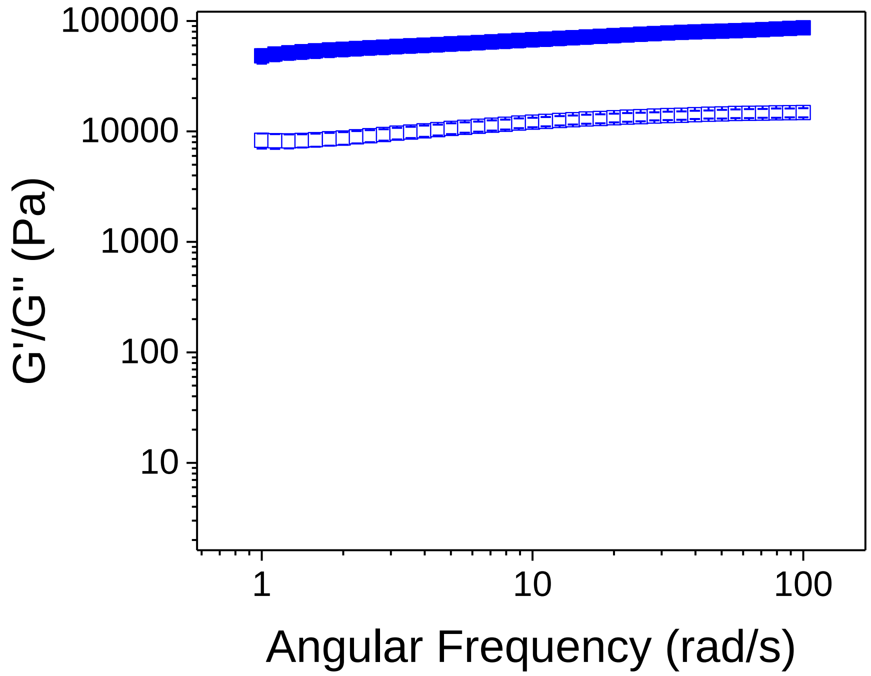

Figure S22. Frequency sweeps of **S** gel at 5:5 mg/mL. Strain of 0.5 % is maintained. Error bars calculated from the standard deviation of three measurements. G’ are the solid shapes and G″ are the empty shapes.

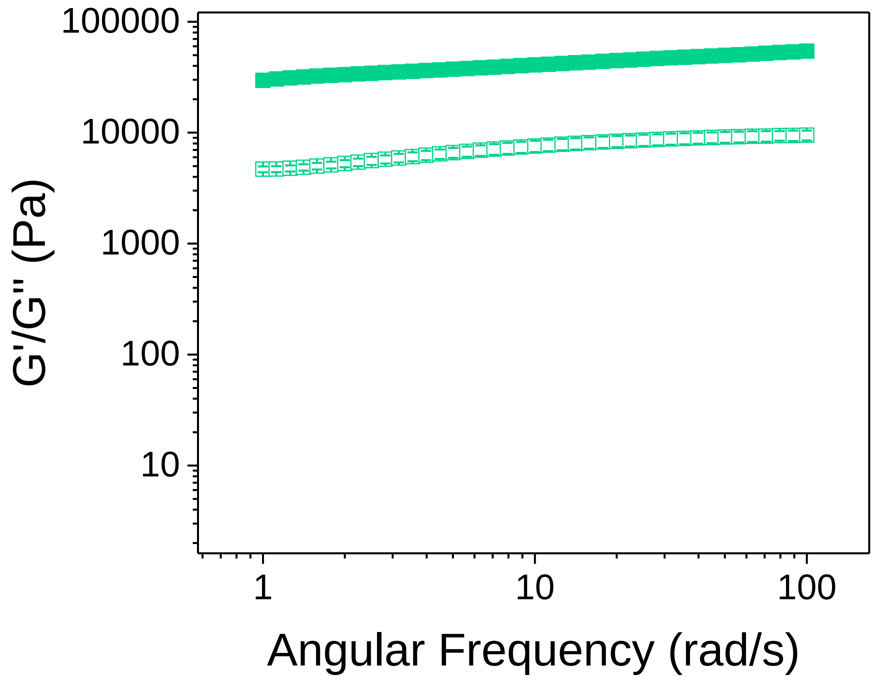

Figure S23. Frequency sweeps of **P** gel at 5:5 mg/mL. Strain of 0.5 % is maintained. Error bars calculated from the standard deviation of three measurements. G’ are the solid shapes and G″ are the empty shapes.

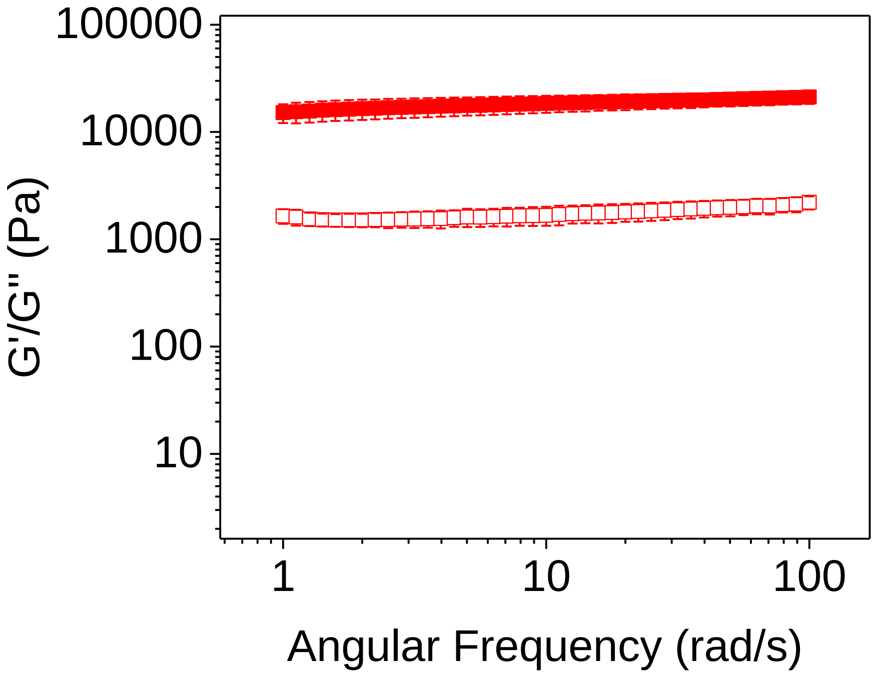

Figure S24. Frequency sweeps of **S*** gel at 5:5 mg/mL. Strain of 0.5 % is maintained. Error bars calculated from the standard deviation of three measurements. G’ are the solid shapes and G″ are the empty shapes.

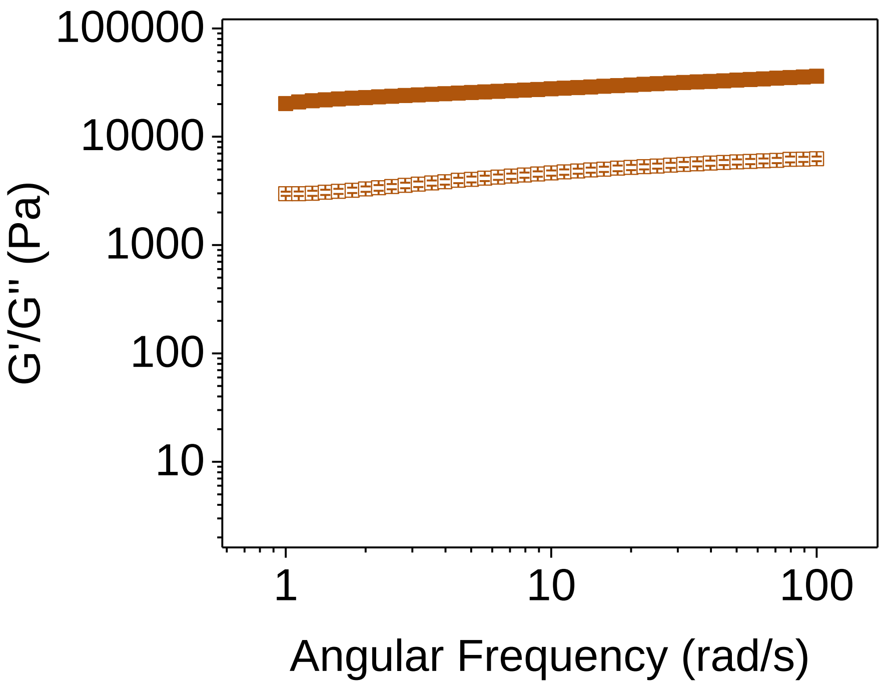

Figure S25. Frequency sweeps of **P*** gel at 5:5 mg/mL. Strain of 0.5 % is maintained. Error bars calculated from the standard deviation of three measurements. G’ are the solid shapes and G″ are the empty shapes.

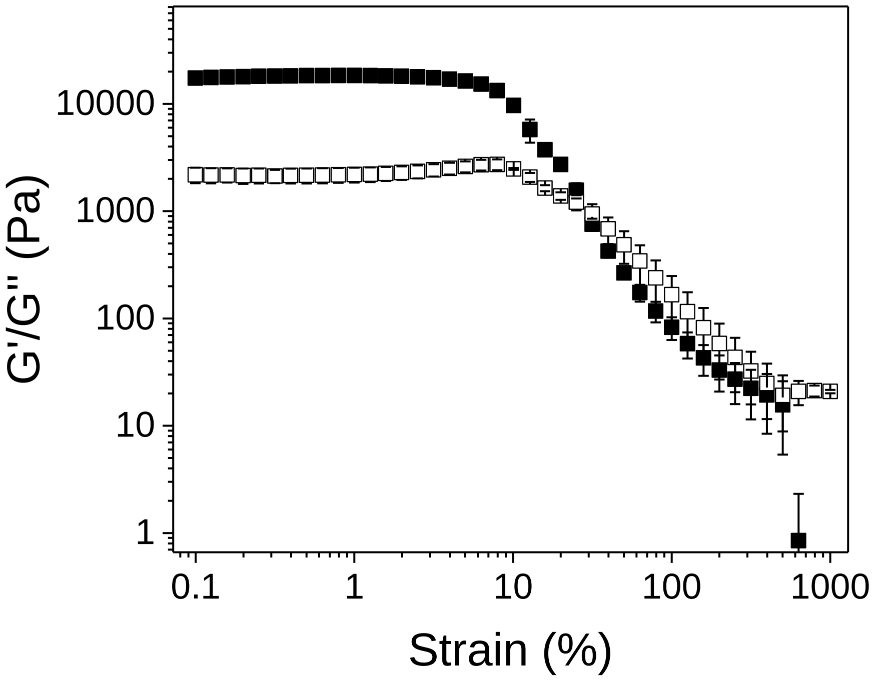

Figure S26. Rheological strain sweeps of gels formed from **1-NapFF** at 5 mg/mL. Error bars calculated from the standard deviation of three measurements. G’ are the solid shapes and G″ are the empty shapes.

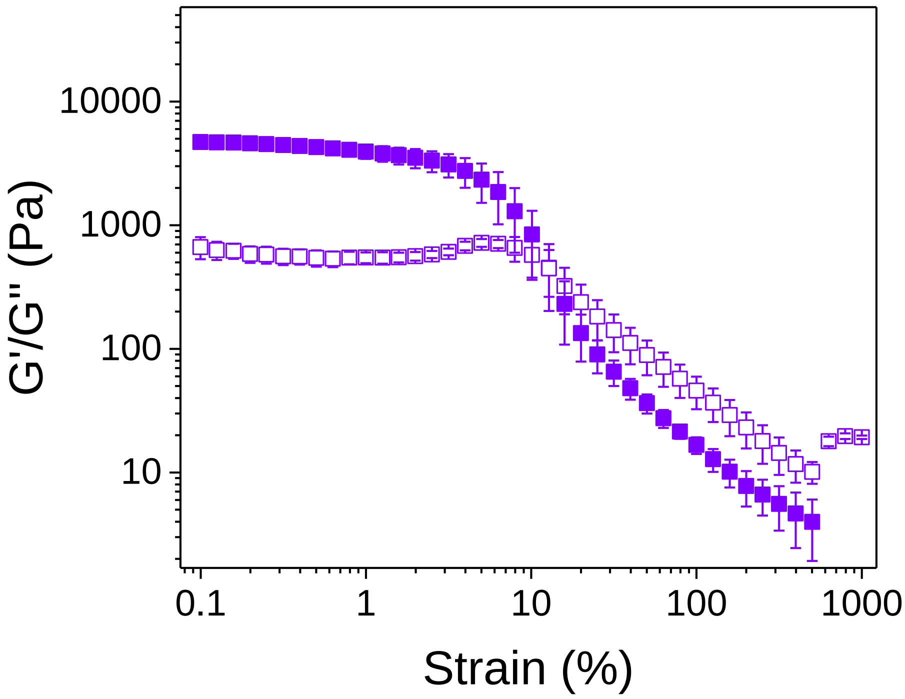

Figure S27. Rheological strain sweeps of gels formed from **NDI-GF** at 5 mg/mL. Error bars calculated from the standard deviation of three measurements. G' are the solid shapes and G″ are the empty shapes.

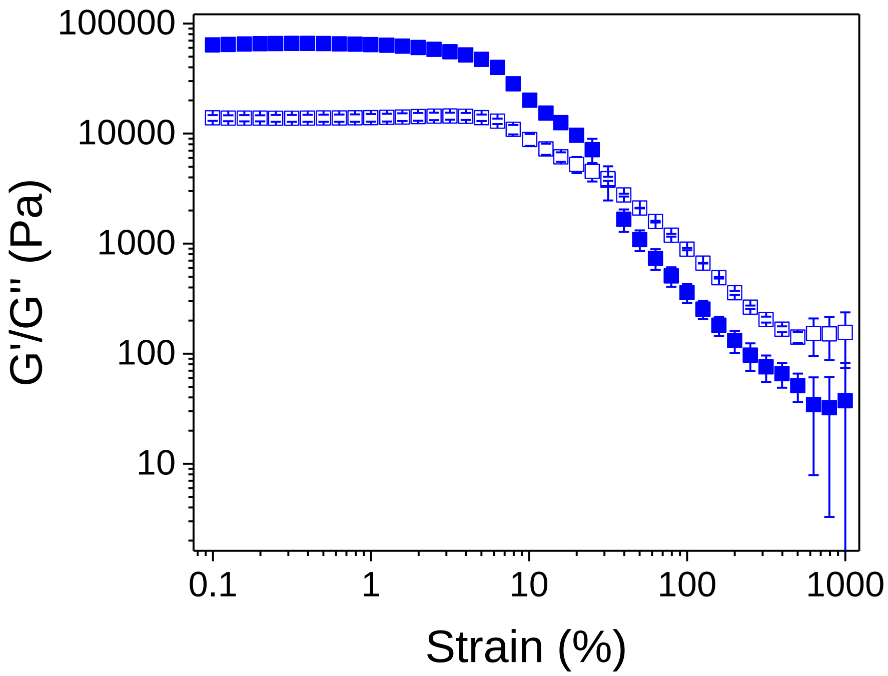

Figure S28. Rheological strain sweeps of gels formed from **S** at 5:5 mg/mL. Error bars calculated from the standard deviation of three measurements. G' are the solid shapes and G″ are the empty shapes.

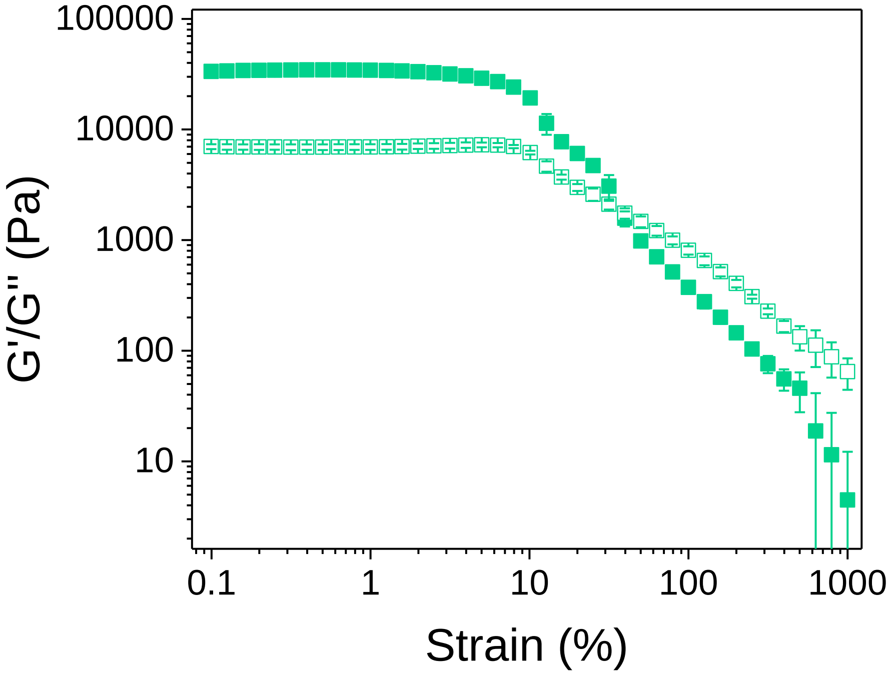

Figure S29. Rheological strain sweeps of gels formed from **P** at 5:5 mg/mL. Error bars calculated from the standard deviation of three measurements. G’ are the solid shapes and G″ are the empty shapes.

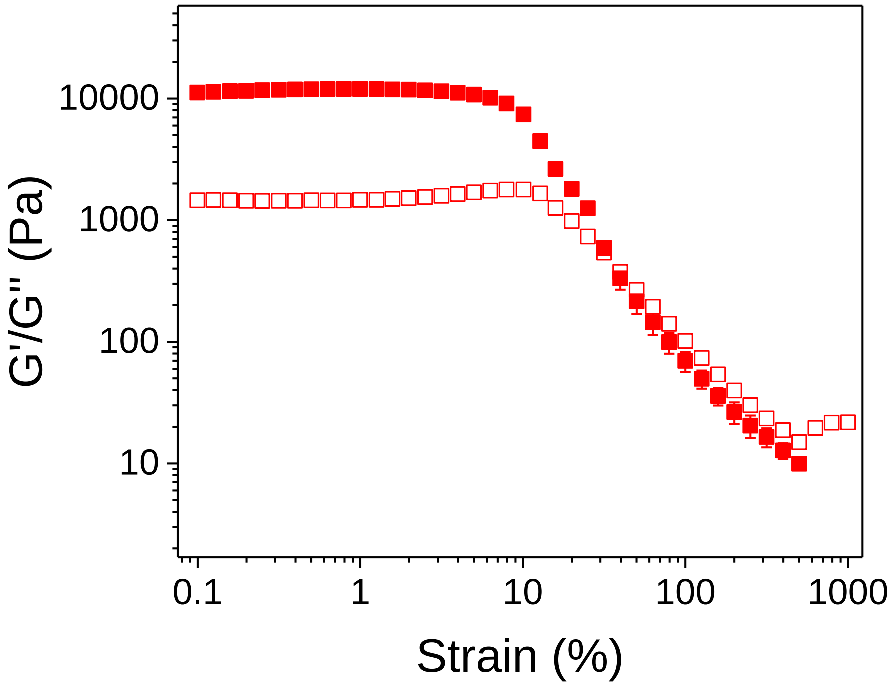

Figure S30. Rheological strain sweeps of gels formed from **S*** at 5:5 mg/mL. Error bars calculated from the standard deviation of three measurements. G’ are the solid shapes and G″ are the empty shapes.

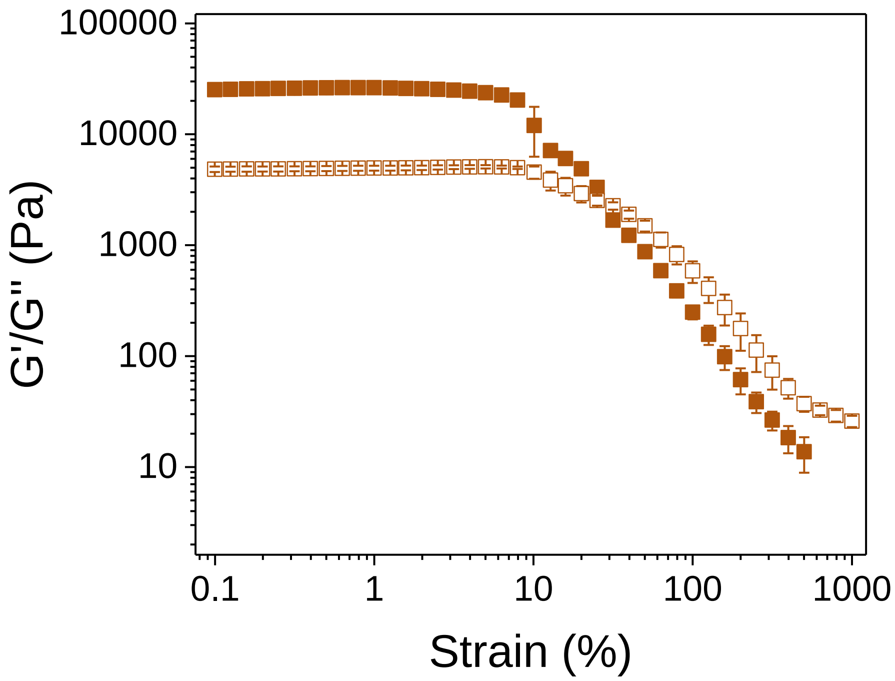

Figure S31. Rheological strain sweeps of gels formed from **P*** at 5:5 mg/mL. Error bars calculated from the standard deviation of three measurements. G’ are the solid shapes and G″ are the empty shapes.

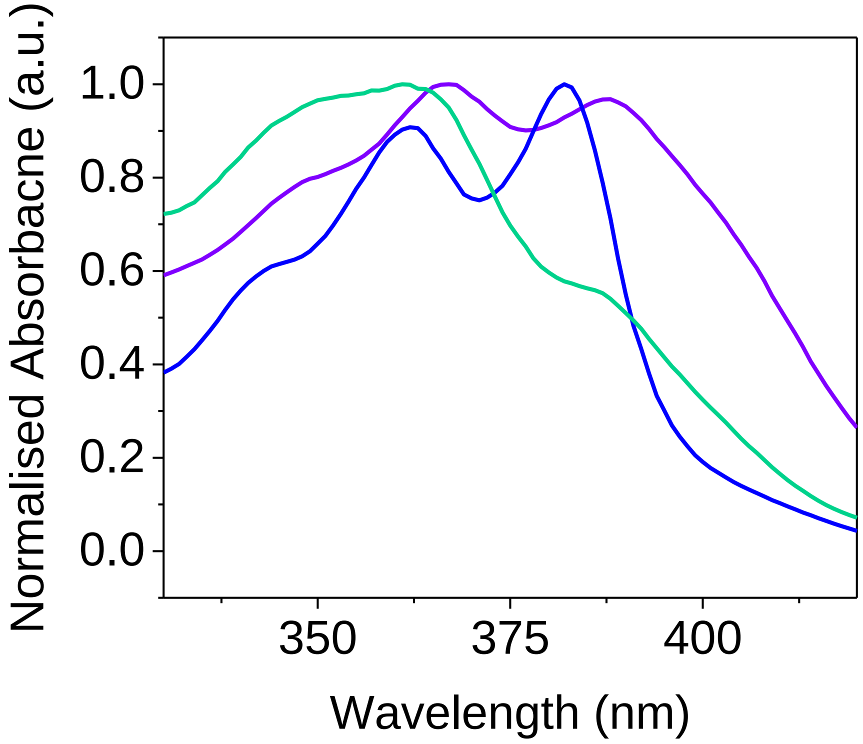

Figure S32. Normalised absorbance spectra of gels formed from **NDI-GF** (–), **S** (–) and **P** (–).

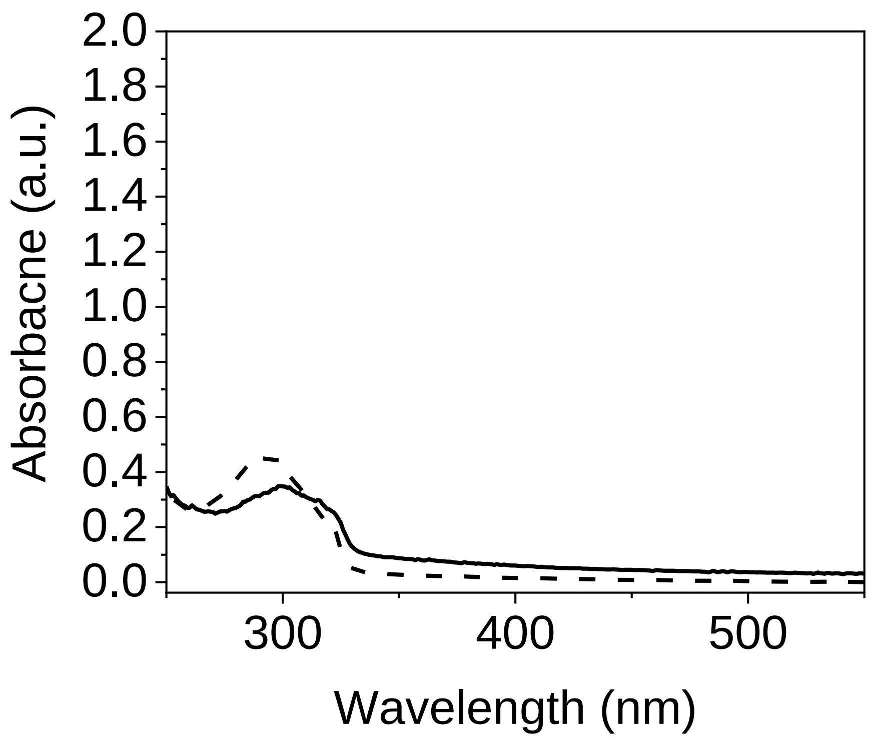

Figure S33. Absorbance spectra of **1-NapFF** at 5 mg/mL as a solution at pH 11 (- - -) and a gel at pH approximately 3.8 (–).

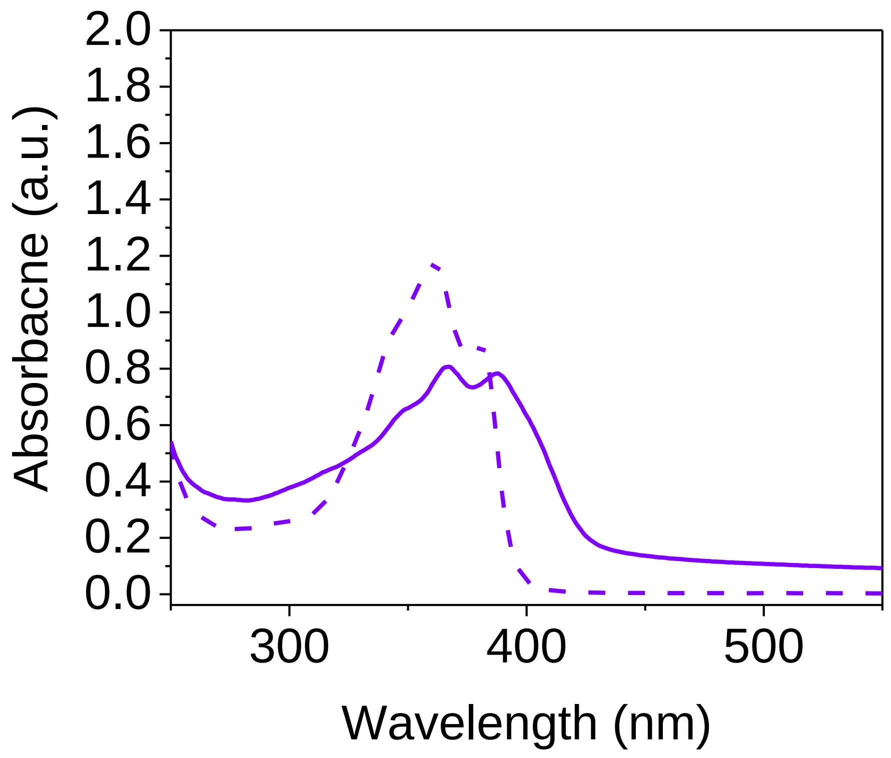

Figure S34. Absorbance spectra of **NDI-GF** at 5 mg/mL as a solution at pH 11 (- - -) and a gel at pH approximately 3.8 (–).

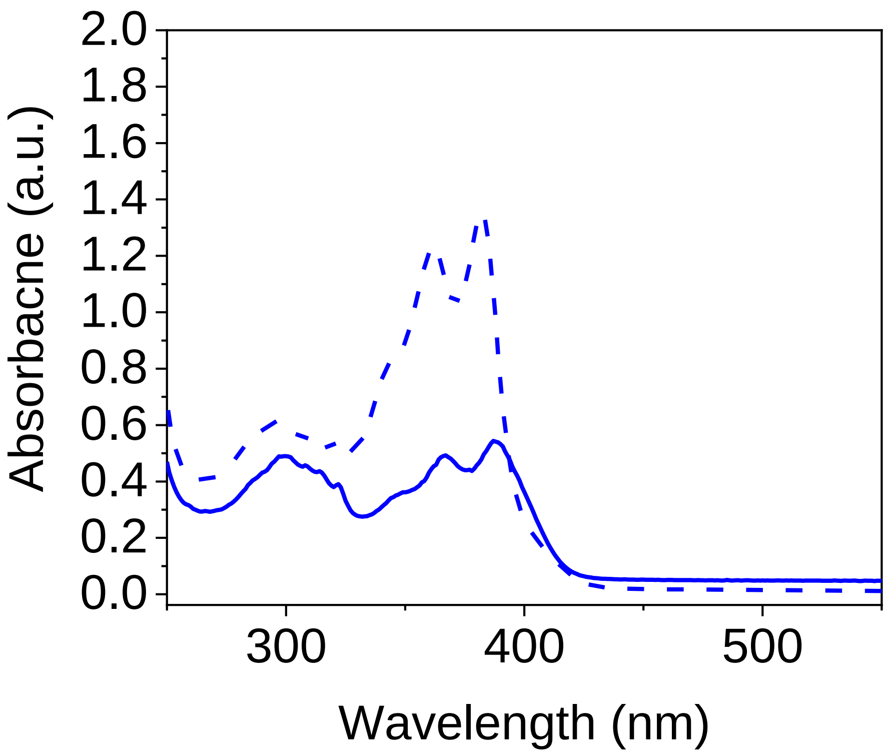

Figure S35. Absorbance spectra of **S** at 5:5 mg/mL as a solution at pH 11 (- - -) and a gel at pH approximately 3.8 (–).

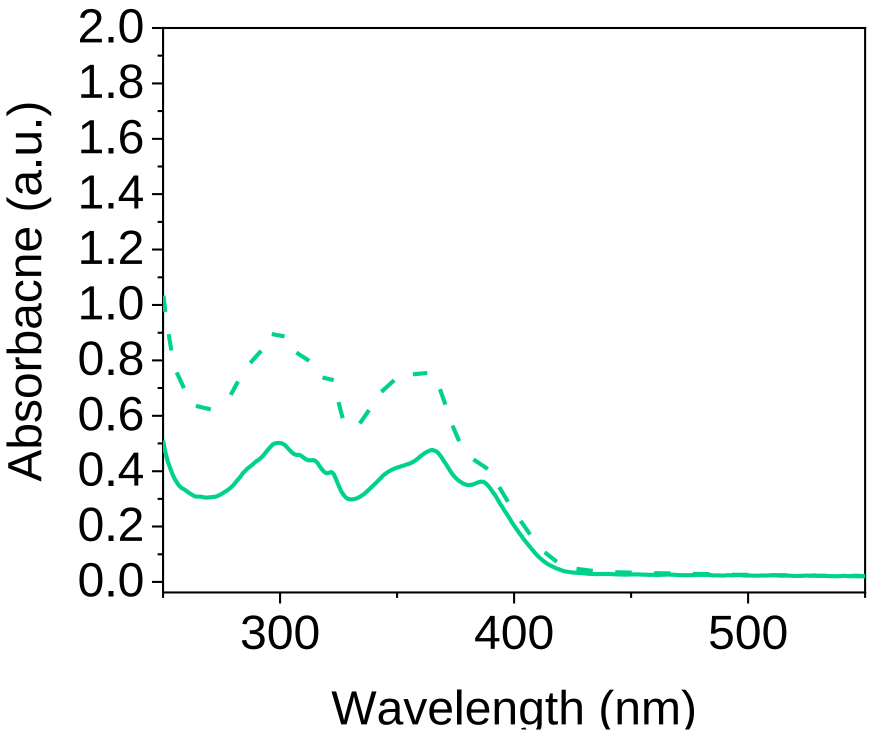

Figure S36. Absorbance spectra of **P** at 5:5 mg/mL as a solution at pH 11 (- - -) and a gel at pH approximately 3.8 (–).

The absorbance spectra of **NDI-GF** does change during gelation, Figure S. The ratio of peaks at 365 and 385 nm in the spectrum of **NDI-GF** switches during gelation, suggesting that packing is changed as the gel forms. As **NDI-GF** does not form well defined structures at high pH this change in packing occurs as larger fibres and aggregates form. This change occurs within the first hour of gelation, Figure S-Figure S.

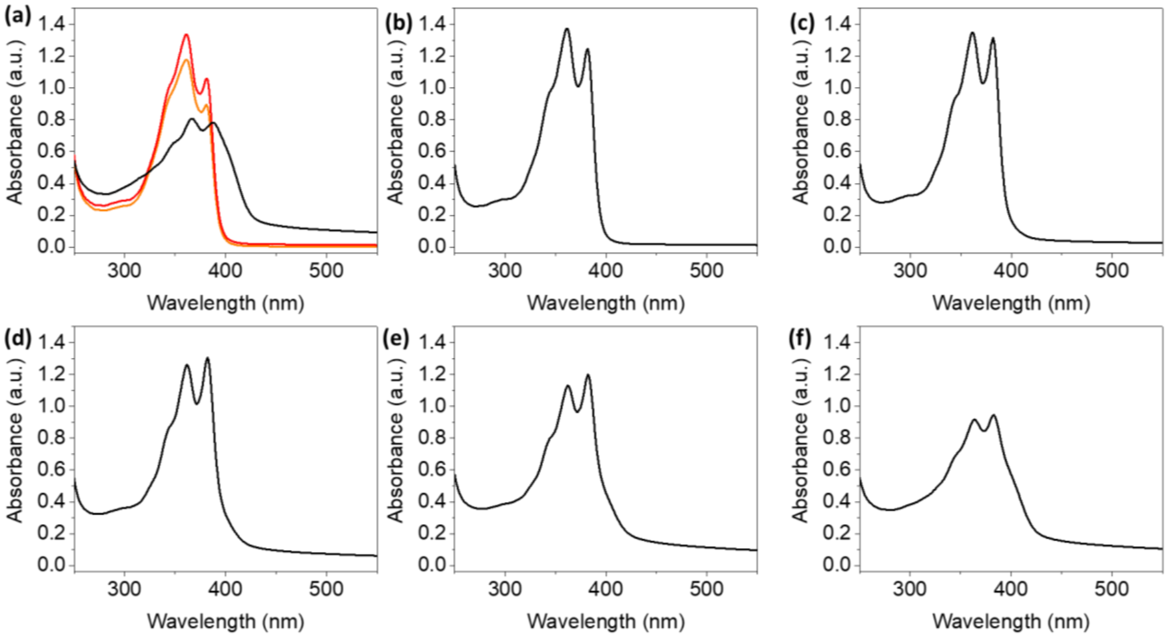

Figure S37. Absorbance spectra of **NDI-GF** at 5 mg/mL (a) as a solution at pH 11 (–), immediately after the addition of GdL (–) and a gel at pH approximately 3.8 (–). Spectra shown after the first (b) 15, (c) 30, (d) 60, (e) 100, (f) 200 minutes of gelation.

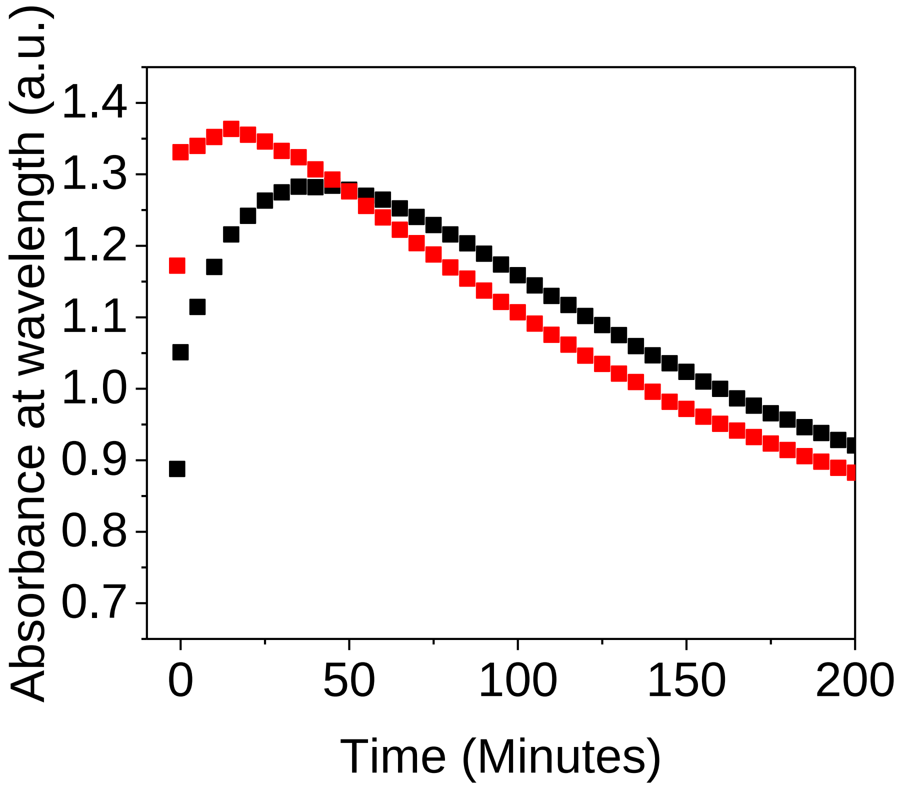

Figure S38. Absorbance of peaks at 380 (■) and 360 nm (■) in absorbance spectra through the first 200 minutes of gelation of 5 mg/mL **NDI-GF**

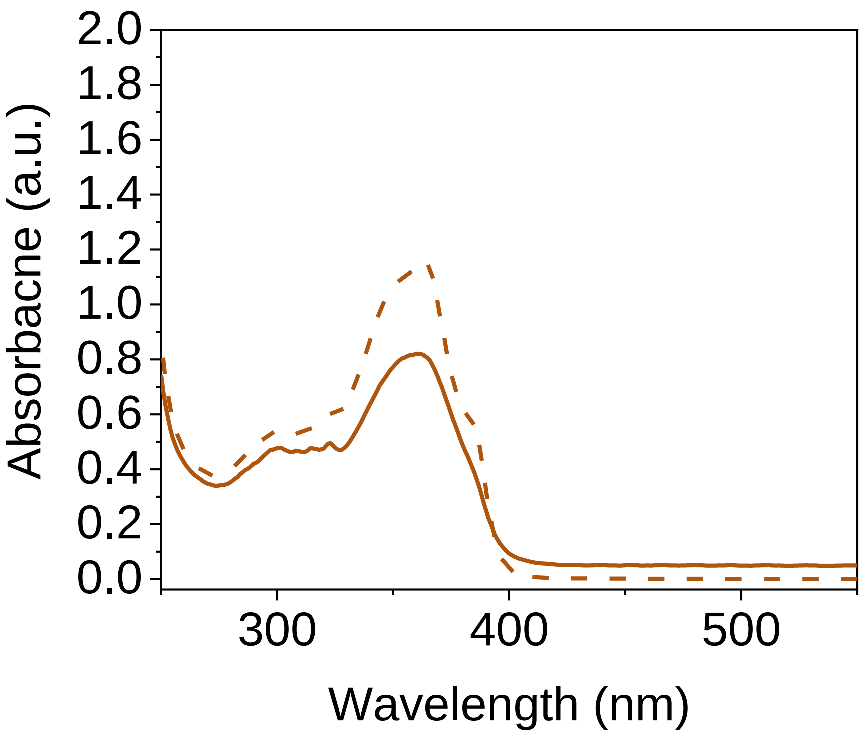

Figure S39. Absorbance spectra of **P*** at 5:5 mg/mL as a solution at pH 11 (- - -) and a gel at pH approximately 3.8 (–).

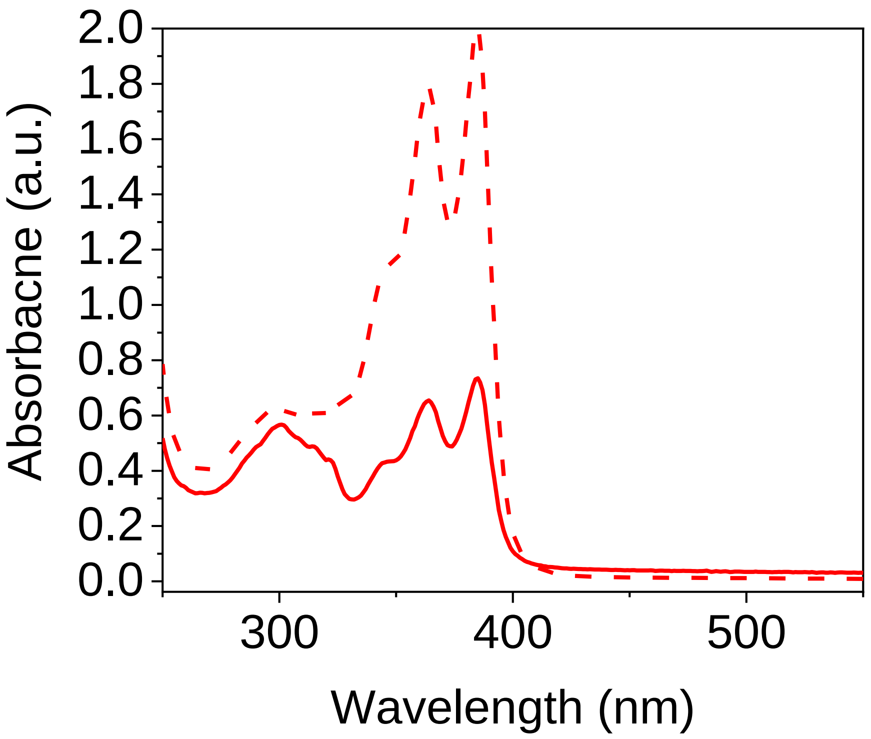

Figure S40. Absorbance spectra of **S*** at 5:5 mg/mL as a solution at pH 11 (- - -) and a gel at pH approximately 3.8 (–).

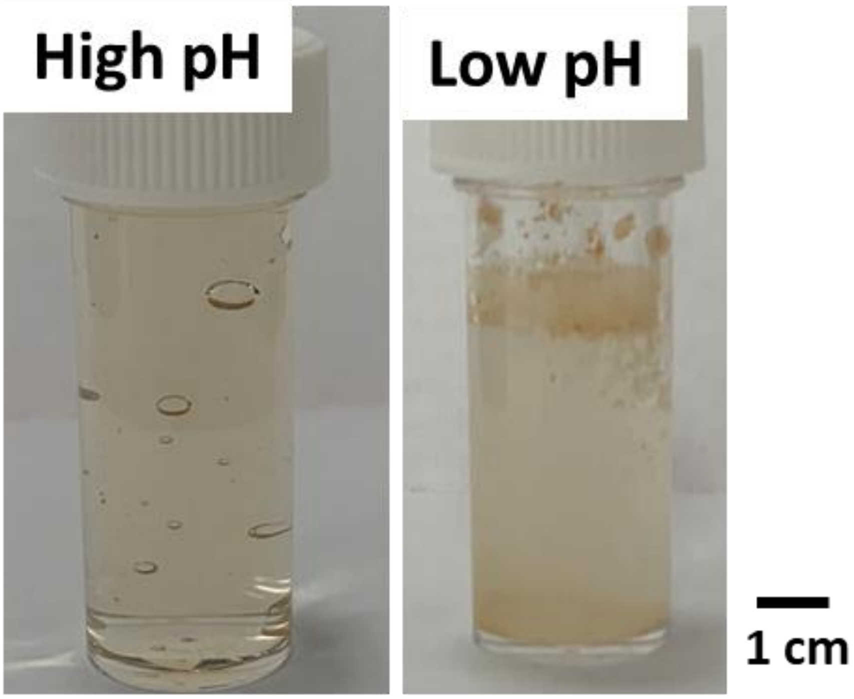

Figure S41. Photograph of precipitating 5 mg/mL **NDI-F** solution below pH 6.

Table S10. Tabulated rheological properties taken from an average of three rheological strain sweep measurements.

Gel	GdL Concentration (mg/mL)	Average pH	G''/G' at 0.5 % = tan(δ)	Yield point (%)	Flow point (%)
**1-NapFF**	4.5	3.88	2158/18323 = 0.12	6.4	31.5
**NDI-GF**	5	3.86	544/4279 = 0.13	2.51	15.9
**S**	9	3.82	13827/65670 = 0.21	4.0	40.6
**P**	9	3.83	6931/34560 = 0.20	6.3	39.8
**S***	10	3.82	1457/11900 = 0.12	6.2	40.1
**P***	10.5	3.83	4914/26180 = 0.19	6.3	31.6

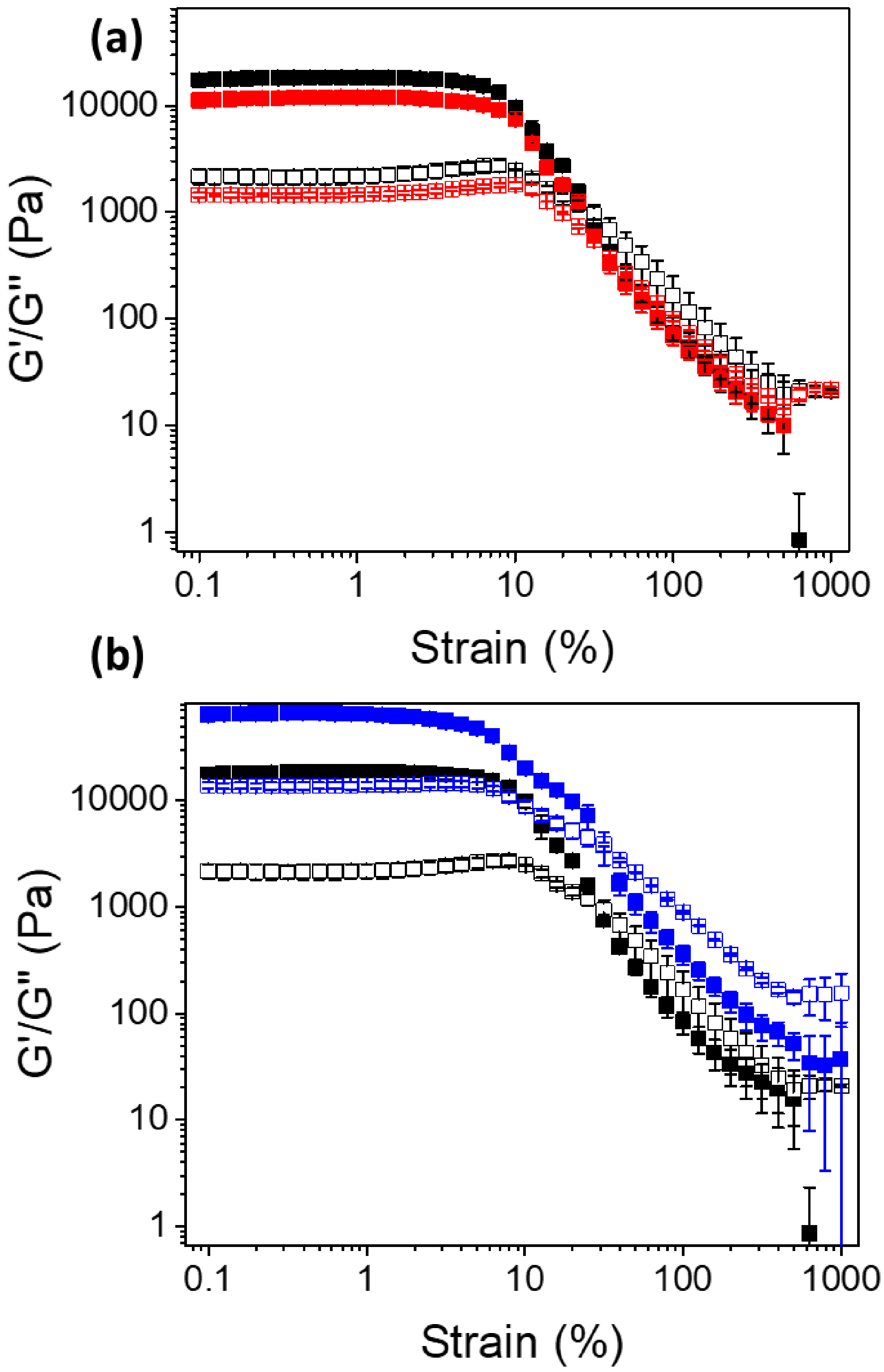

Figure S42. Rheological strain sweeps of gels formed from **1-NapFF** (■) and (a) **S*** (■) and (b) **S** (■). Measurement taken in triplicate and error bars are calculated from standard deviation. G’ are the solid shapes and G″ are the empty shapes.

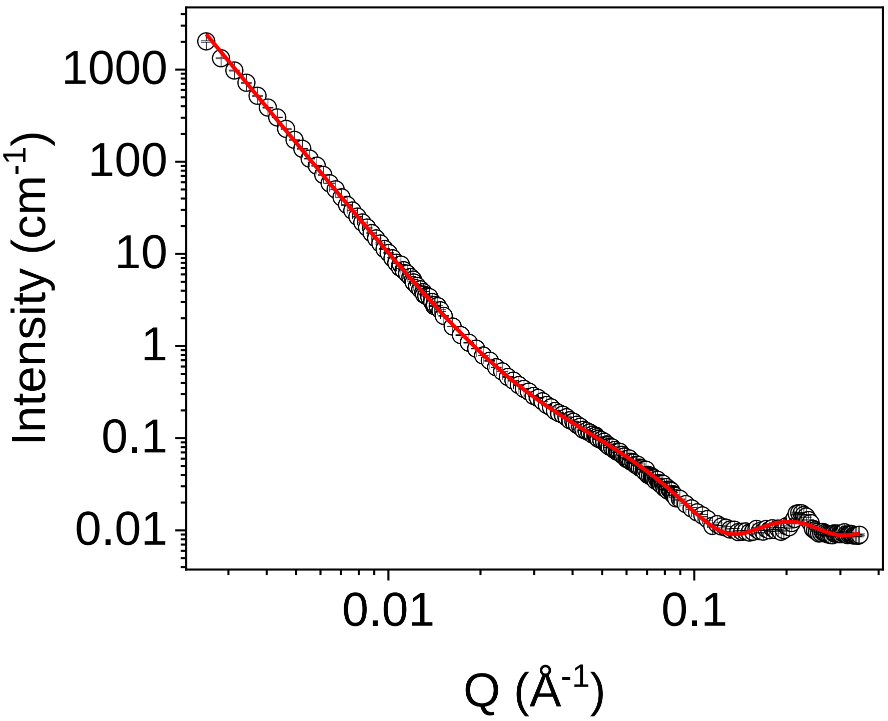

Figure S43. Small angle neutron scattering data of **1-NapFF** gel (O) fitted to a hollow cylinder model combined with power law (–).

Table S11. Parameters of SANS model fit above.

**1-NapFF** gel	Hollow cylinder (HC) and power law (PL)	
	**Value**	**Error**
Background (cm^{-1})	0.008803	3.29E-05
Scale A (HC)	0.001692	4.13E-06
Thickness HC (Å)	6.5084	0.016042
Radius HC (Å)	14.58	0.042552
Length HC (Å)	3819.1	97.455
Scale B (PL)	8.60E-08	1.35E-10
Power	4.0271	0.0003297
Range	0.00254305-0.346809	
Chi^2	11.738	

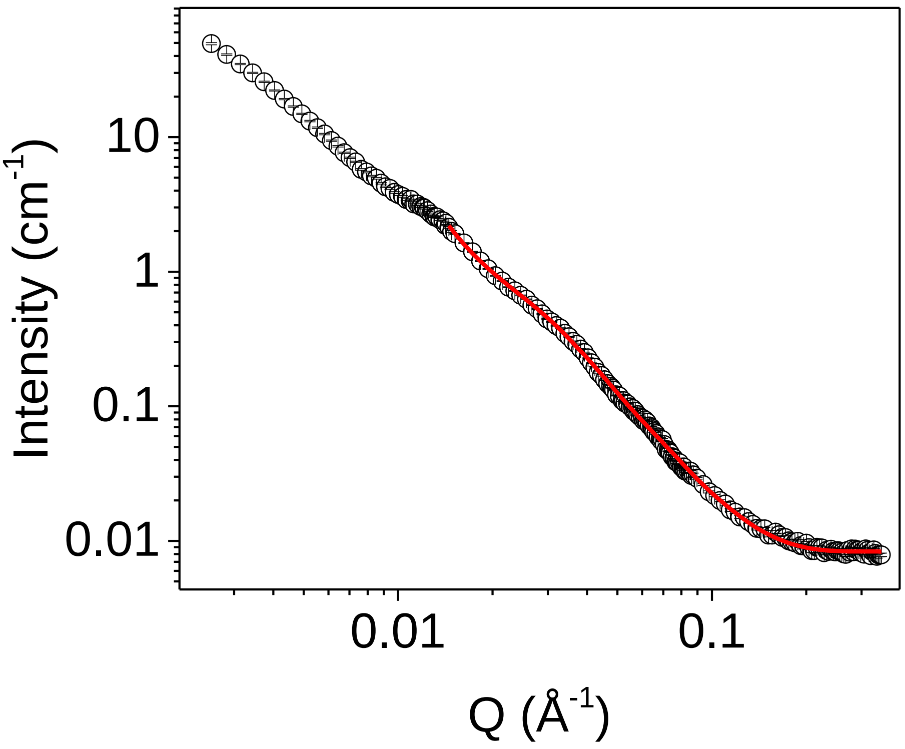

Figure S44. Small angle neutron scattering data of **NDI-GF** gel (O) fitted to a flexible elliptical cylinder model combined with a power law (–).

Table S12. Parameters of SANS model fit above.

**NDI-GF** gel	Flexible elliptical cylinder (FEC) and power law (PL)	
	**Value**	**Error**
Background (cm^{-1})	0.008282	3.42E-05
Scale A (FEC)	0.000198	2.80E-07
Axis Ratio FEC	5.966	0.008497
Radius FEC (Å)	12.767	0.020861
Kuhn Length FEC (Å)	30.323	0.10486
Length FEC (Å)	303.22	0.0042516
Scale B (PL)	2.13E-09	1.34E-11
Power	4.7819	0.0015609
Range	0.0145093-0.346809	
Chi^2	4.1465	

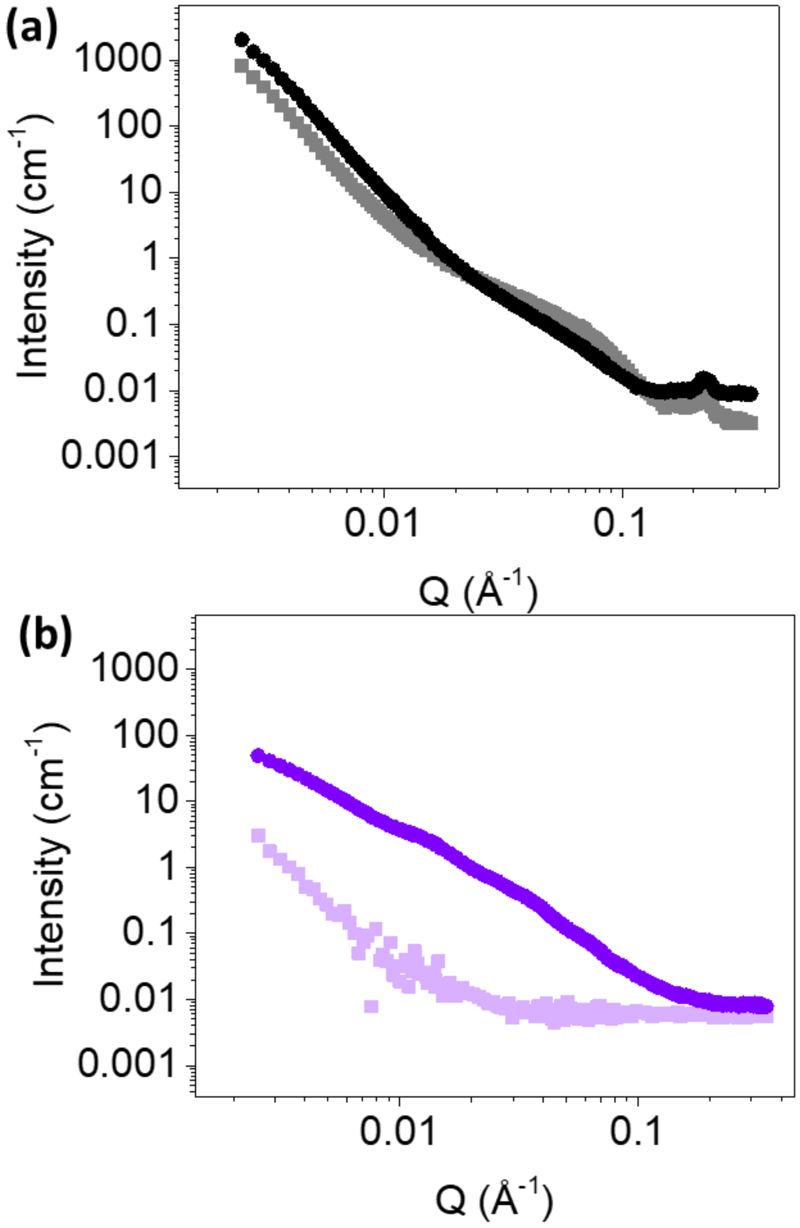

Figure S45. Small angle neutron scattering data from solutions of (a) **1-NapFF** and (b) **NDI-GF** at pD 11 and gel state. A lighter colour is also used to show data from pD 11.

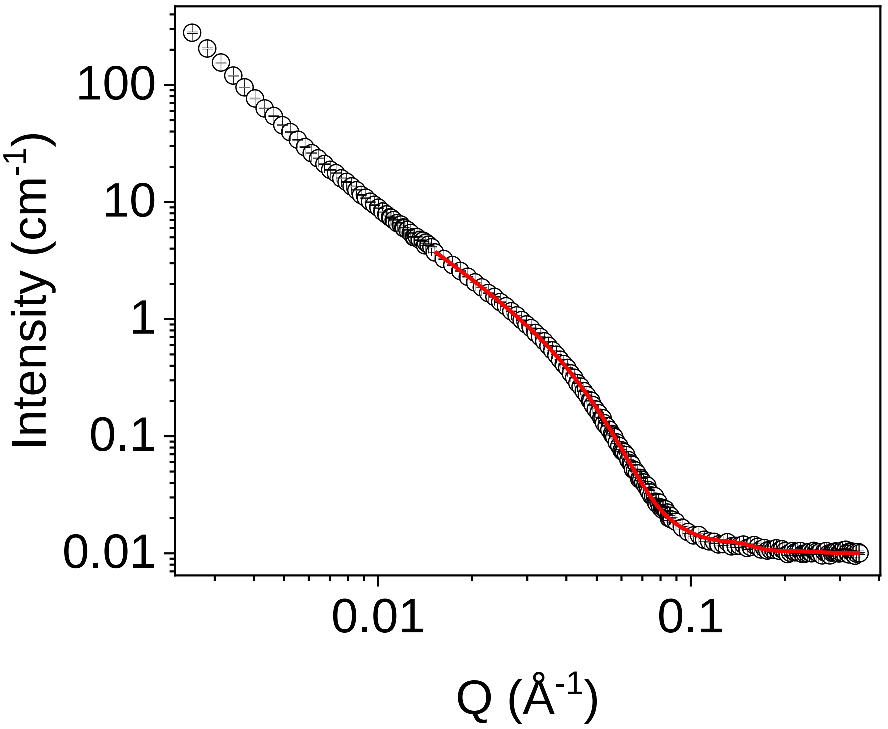

Figure S46. Small angle neutron scattering data of **P** gel (O) fitted to a flexible elliptical cylinder model combined with a power law (–).

Table S13. Parameters of SANS model fit above.

**P** gel	Flexible elliptical cylinder (FEC) and power law (PL)	
	**Value**	**Error**
Background (cm^{-1})	0.009996	3.23E-05
Scale A (FEC)	0.000732	8.55E-07
Axis Ratio FEC	1.7125	0.001487
Radius FEC (Å)	34.674	0.027923
Kuhn Length FEC (Å)	105.19	0.39547
Length FEC (Å)	1051.9	15.904
Scale B (PL)	2.02E-06	3.23E-09
Power	3.0153	0.000473
Range	0.015-0.346809	
Chi^2	4.729	

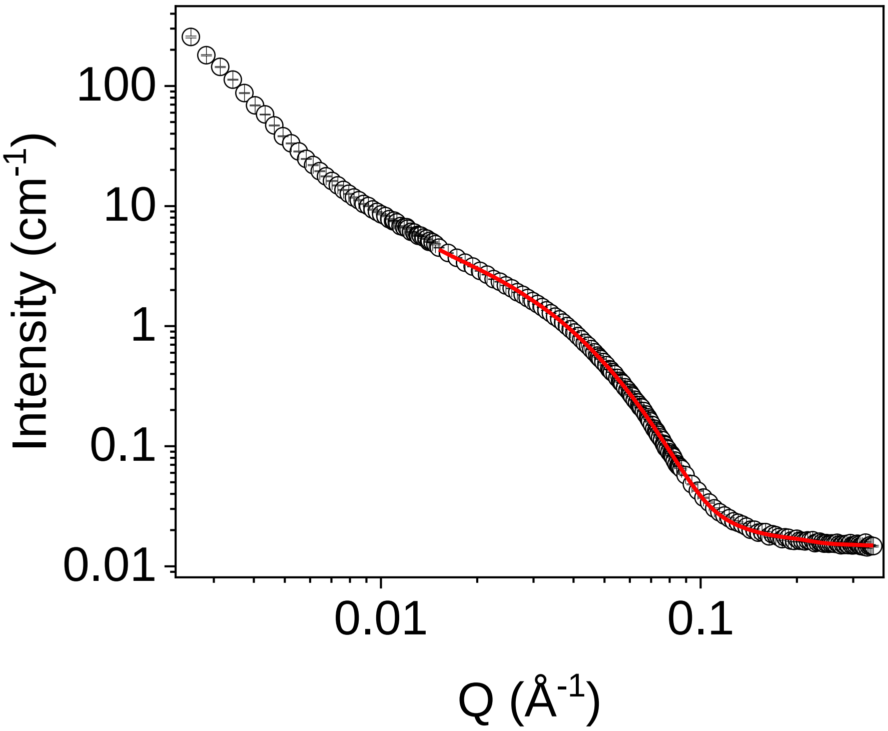

Figure S47. Small angle neutron scattering data of **S** gel (O) fitted to a flexible elliptical cylinder model combined with a power law (–).

Table S14. Parameters of SANS model fit above.

**S** gel	Flexible elliptical cylinder (FEC) and power law (PL)	
	**Value**	**Error**
Background (cm^{-1})	0.014468	3.44E-09
Scale A (FEC)	0.001206	1.02E-06
Axis Ratio FEC	1.6768	0.00119
Radius FEC (Å)	24.85	0.018026
Kuhn Length FEC (Å)	33.379	0.06219
Length FEC (Å)	430.2	1.3079
Scale B (PL)	1.88E-05	3.40E-08
Power	2.7232	0.001183
Range	0.015-0.346809	
Chi^2	5.0415	

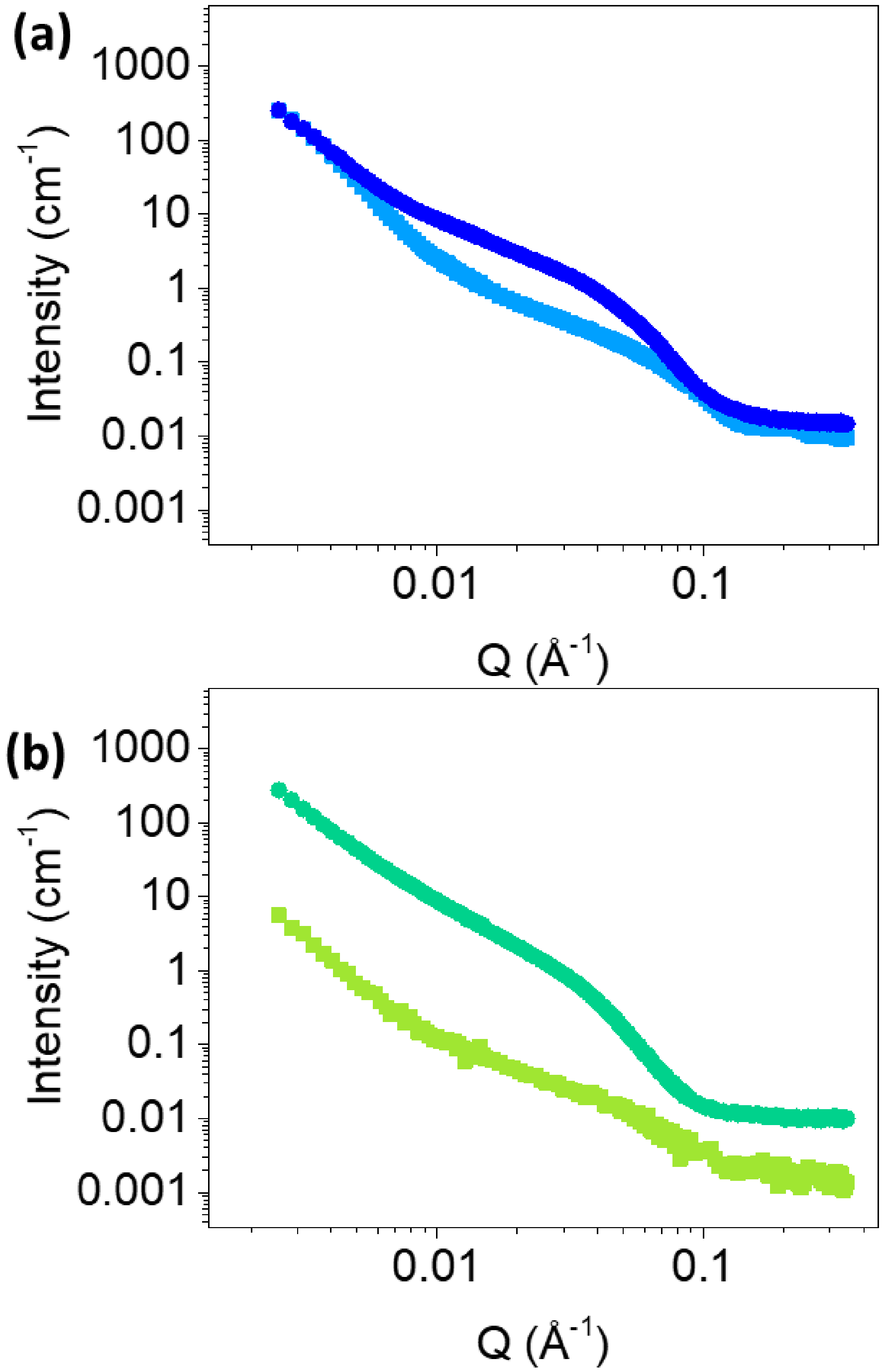

Figure S48. Small angle neutron scattering data from solutions of (a) **S** and (b) **P** at pD 11 and gel state. A lighter green and lighter blue are used to show data from pD 11.

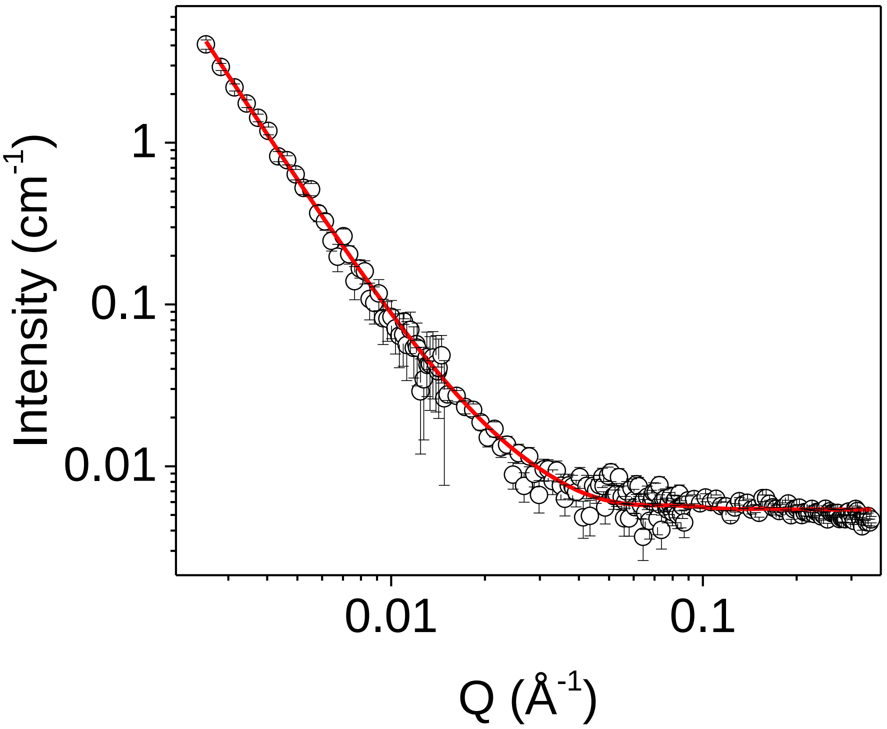

Figure S49. Small angle neutron scattering data of **NDI-F** at pD 6 (O) fitted to a hollow cylinder model combined power law (–).

Table S15. Parameters of SANS model fit above.

**NDI-F** pD 6	Hollow cylinder (HC) and power law (PL)	
	**Value**	**Error**
Background (cm^{-1})	0.005186	8.20E-12
Scale A (HC)	3.37E-05	4.35E-06
Thickness HC (Å)	3.3741	0.43449
Radius HC (Å)	42.01	4.3439
Length HC (Å)	349.96	254.61
Scale B (PL)	9.98E-08	1.46E-09
Power	2.9391	0.0028102
Range	0.00254305-0.346809	
Chi^2	1.98	

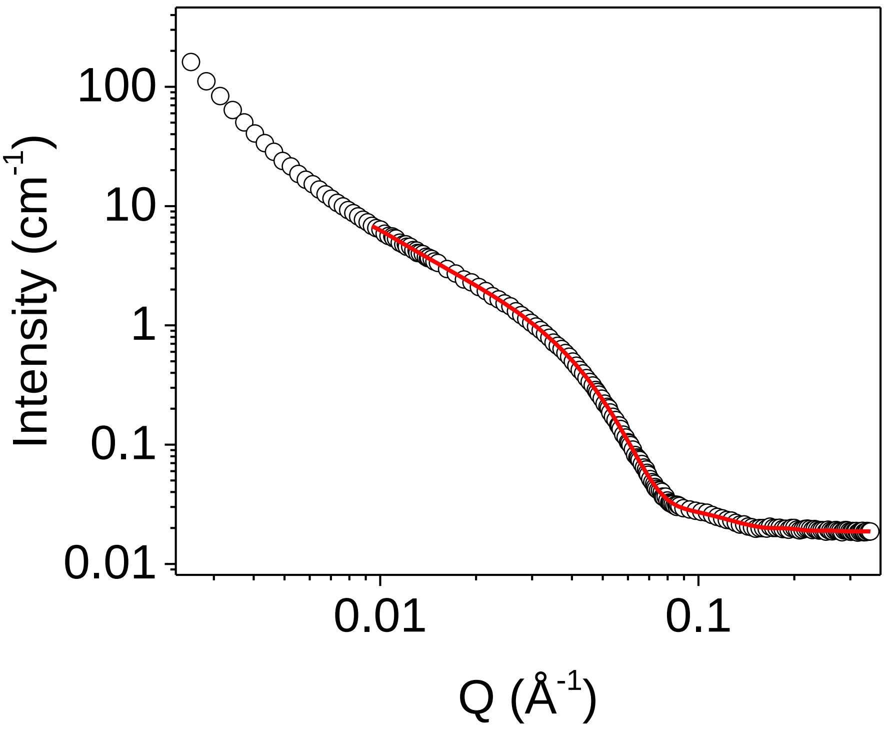

Figure S50. Small angle neutron scattering data of **S*** gel (O) fitted to a hollow cylinder model combined a flexible elliptical cylinder model (–).

Table S16. Parameters of SANS model fit above.

**S*** gel	Hollow cylinder (HC) and flexible elliptical cylinder (FEC)	
	**Value**	**Error**
Background (cm^{-1})	0.018612	3.36E-05
Scale A (HC)	0.000285	5.97E-06
Thickness HC (Å)	13.447	0.27741
Radius HC (Å)	31.618	0.28595
Length HC (Å)	51.336	1.9571
Scale B (FEC)	1.23E-03	1.23E-06
Axis Ratio FEC	1.6834	0.001399
Radius FEC (Å)	35.736	0.024218
Kuhn Length FEC (Å)	325.67	1.98572
Length FEC (Å)	947.02	61.181
Range	0.00942397-0.346809	
Chi^2	1.617	

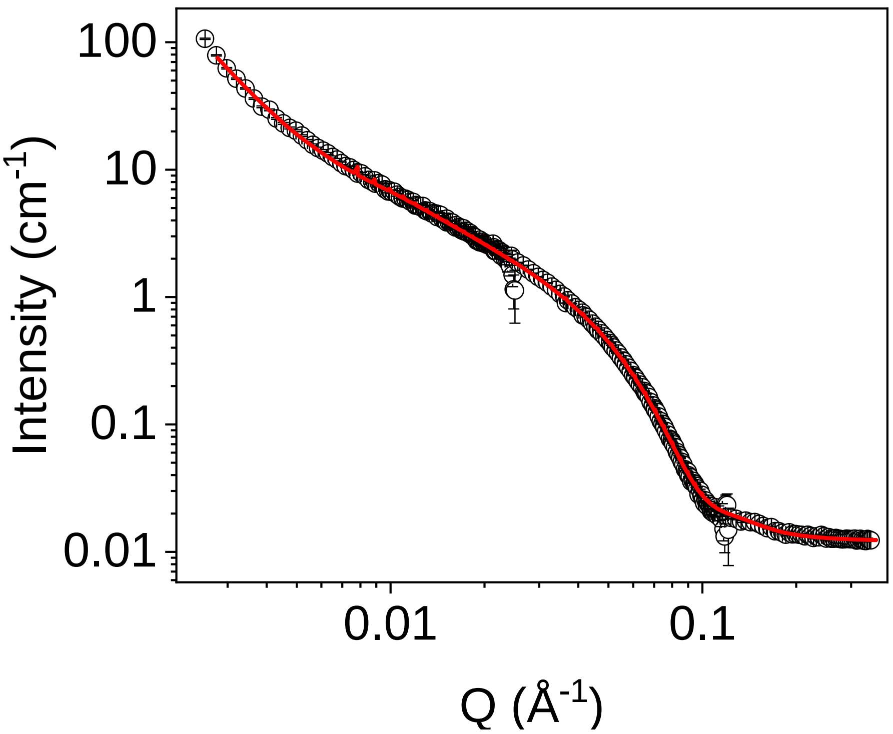

Figure S51. Small angle neutron scattering data of **P*** gel (O) fitted to a flexible cylinder model combined with a power law (–).

Table S17. Parameters of SANS model fit above.

**P*** gel	Flexible cylinder (FC) and power law (PL)	
	**Value**	**Error**
Background (cm^{-1})	0.01221	2.21E-05
Scale A (FC)	0.003521	2.14E-06
Radius FC (Å)	35.641	0.018536
Polydispersity of Radius FC	0.2	
Kuhn Length FC (Å)	311.28	3.6998
Length FC (Å)	376.27	2.18889
Scale B (PL)	4.86E-06	1.85E+08
Power	2.7888	0.000751
Range	0.00276167-0.34681	
Chi^2	8.9877	

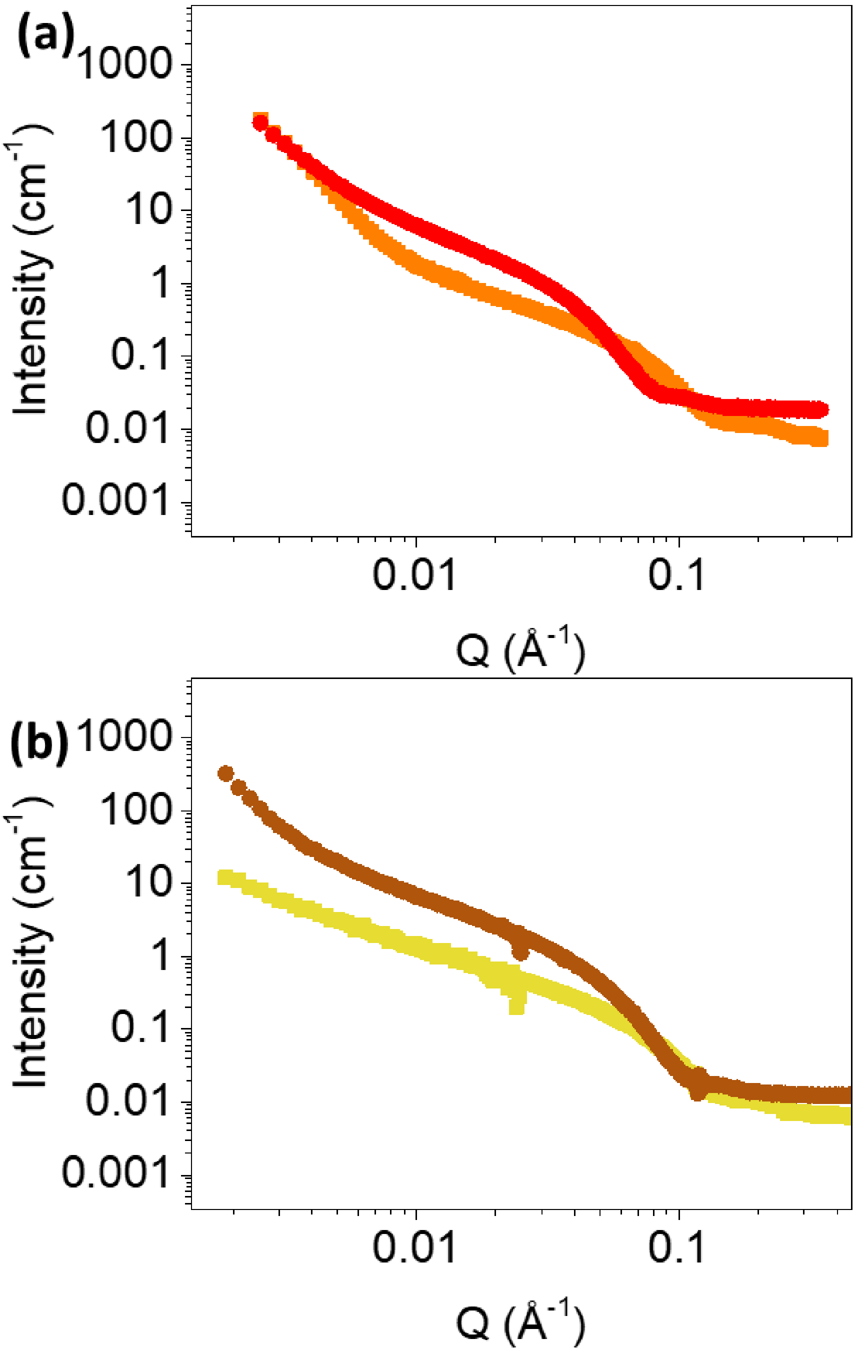

Figure S52. Small angle neutron scattering data from solutions of (a) **S*** and (b) **P*** at pD 11 and gel state. A lighter orange and yellow colour are also used to show data from pD 11.

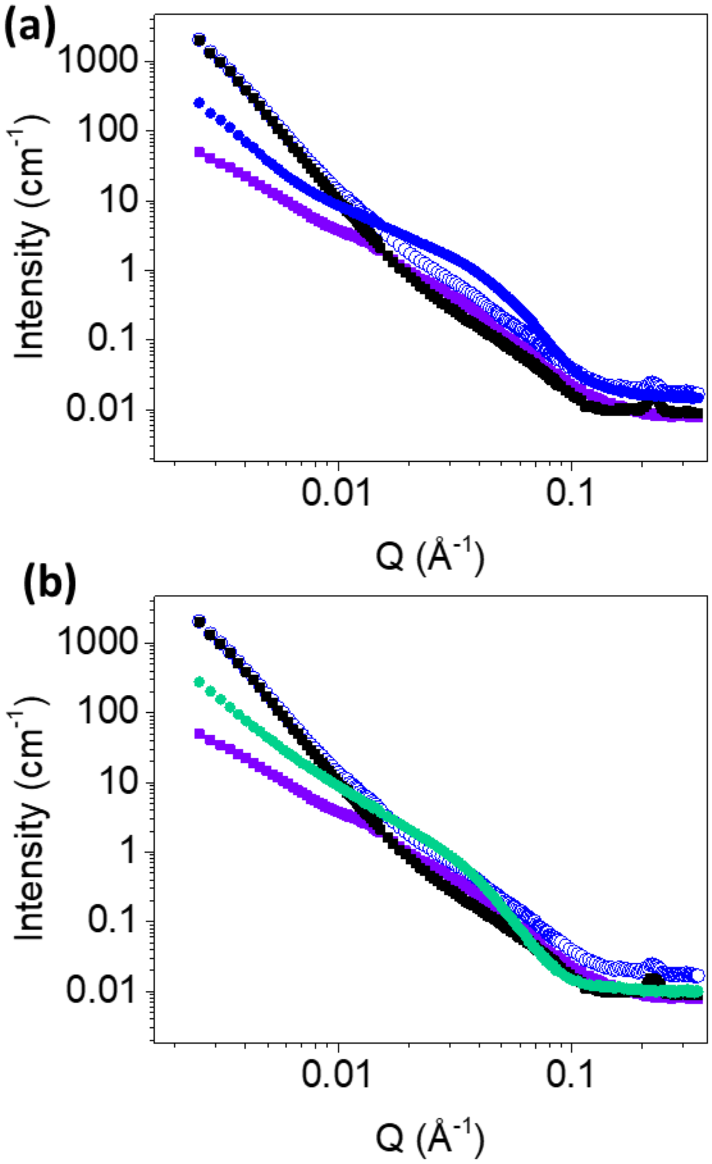

Figure S53. Small angle neutron scattering data from **1-NapFF** (■), **NDI-GF** (■) and (c) **S** (●) and (d) **P** (●) gels. Simulated scattering data created by a simple addition of raw data of each component is shown for **S/P** (O).

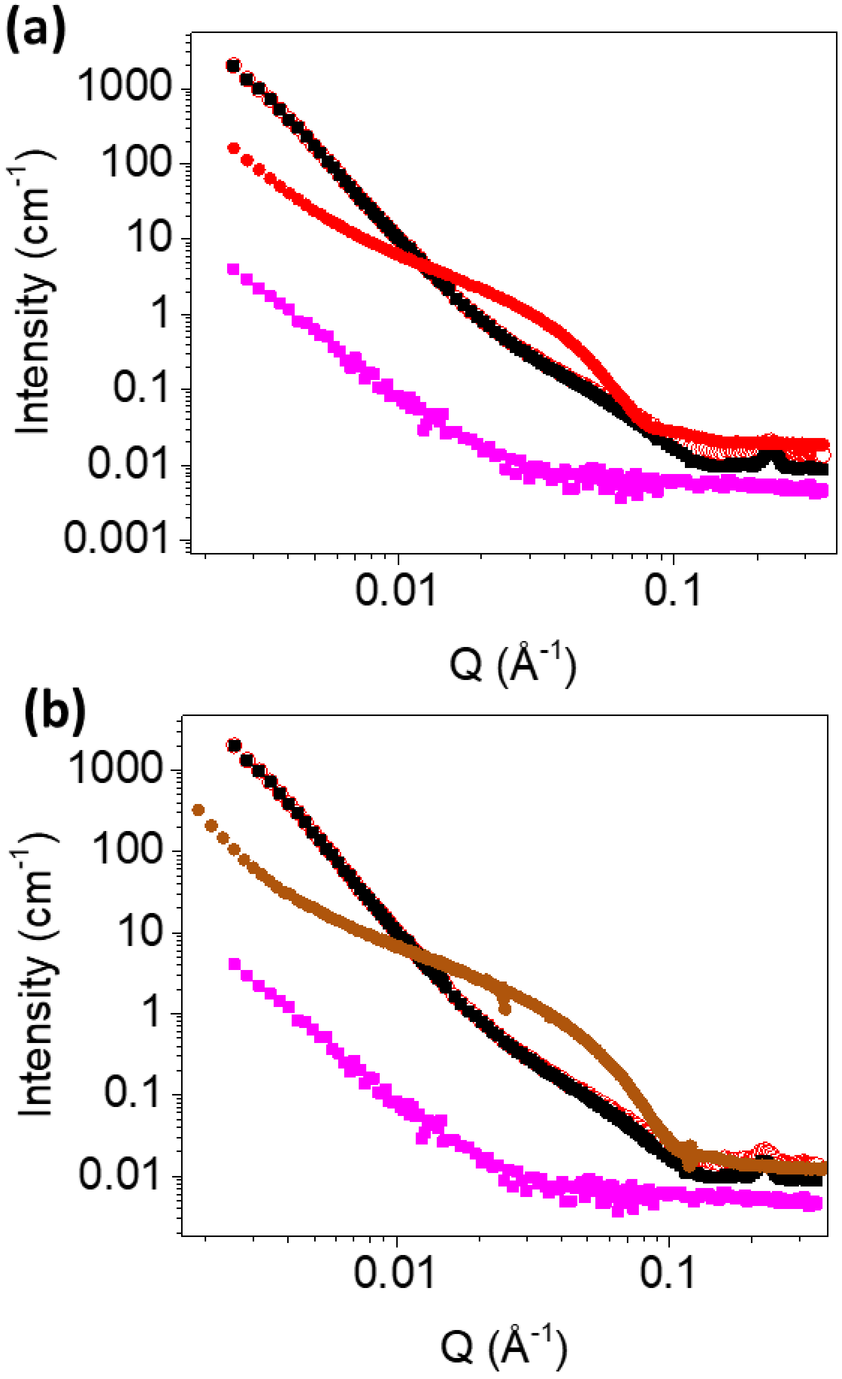

Figure S54. Small angle neutron scattering data from **1-NapFF** (■), **NDI-F** (■) and (a) **S*** (●) and (b) **P*** (●) gels (or solution at pD 6 in the case of non-gelating **NDI-F** (■)**.** Simulated scattering data created by a simple addition of raw data of each component is shown for **S*/P** (O).

DISCUSSION ABOUT SANS DATA OF S AND P

At high pD, Kuhn length is larger in mixes than individual components which suggests more rigid structures. Other parameters are relevantly comparable to their respective components. As **1-NapFF** forms defined structures at high pD (shown by the high scattering intensity) the degree of freedom for the NDI component may be reduced in mixes.

The fit of **NDI-GF** as a gel is to a flexible elliptical cylinder model combined with a power law, Figure S and Table S12. As pD is lowered, aggregates become larger as the radius increases (while axis ratio of **NDI-GF** decreases as cylinders are more defied) in both NDIs. Structures of **NDI-GF** become more flexible as Kuhn length decreases. The parameters of **1-NapFF** do not greatly change through gelation, Figure S and Table S11. The radii of all mixes generally increase as gels, Figure S-Figure STable S12 and Table S13-Table S14.

P fits to the same model as gel as at high pD. During gelation the radius of flexible cylinders are smaller, axis ratio is comparable and the Kuhn length decreases (which suggests more flexible structures), Figure S and Table S13. This trend is not comparable with the trends of **NDI-GF. S** data fits to a different model as a gel compared to at high pD, changing from a combination of flexible elliptical cylinders and hollow cylinders to flexible cylinders only, Figure S and Table S14.

The flexibility of **P** gels is less than **NDI-GF** gels whereas the flexibility of **S** gels is comparable. This suggest than addition of **1-NapFF** can influence flexibility of fibres, but preparation method also impacts this parameter. The radii of cylinders in both mixes are smaller than the NDI component and larger than **1-NapFF** as single component systems. The axis ratios of **S** and **P** gels are also smaller than **NDI-GF** gels. This data suggests that NDIs disrupt the **1-NapFF** structures within gels.

DISCUSSION ABOUT SANS DATA OF S* AND P*

As a gel, **S*** fits to the same model as at pD 11 (a combination of flexible elliptical and hollow cylinders) but the parameters are not comparable to high pD, Figure S, Table S16. As with the single NDI components, radius increases as pD is lowered and flexible cylinders become more rigid (characterised by an increase in Kuhn length). The length of hollow cylinders whereas the length of flexible cylinders decreases. This model suggests the structures are still more flexible than either component alone as a simple hollow cylinder model does not fit to this data.

P* data fits to a different model as a gel compared to at high pD. This mix changed from a combination of flexible elliptical cylinders and hollow cylinders to flexible cylinders only, Figure S and Table S17. **P*** requires a polydispersity factor upon the radius which suggests that a range of radii are in solution, Table S17. The radius increases as axis ratio significantly decreases as comparable with their single NDI analogues as pD is lowered. The trends of **P*** are also comparable to those of **S*,** and the cylinders become more rigid in gel state. The radii of cylinders in all mixes are smaller than the NDI component and larger than **1-NapFF** as single component systems. **NDI-F** containing mixes form more rigid structures than **NDI-GF** containing mixtures which may be due to the additional flexibility of **NDI-GF** structures allowed by the glycine functionality of the dipeptide and the fact that **NDI-F** alone forms more rigid structures.

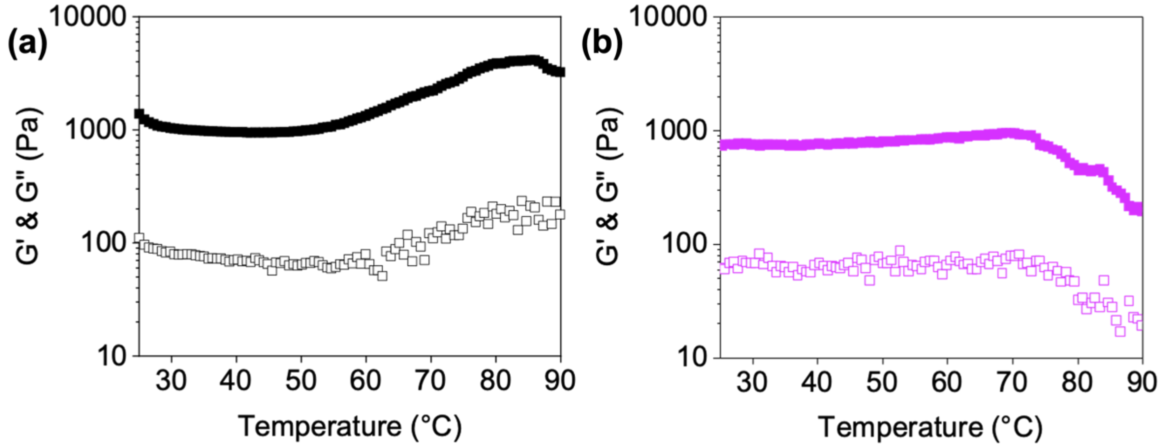

Figure S55. Hydrogel temperature sweeps for (a) **1-NapFF** and (b) **NDI-GF**. G' are the solid shapes and G″ are the empty shapes. Tests performed at 0.5% strain and 10 rads^{-1}.

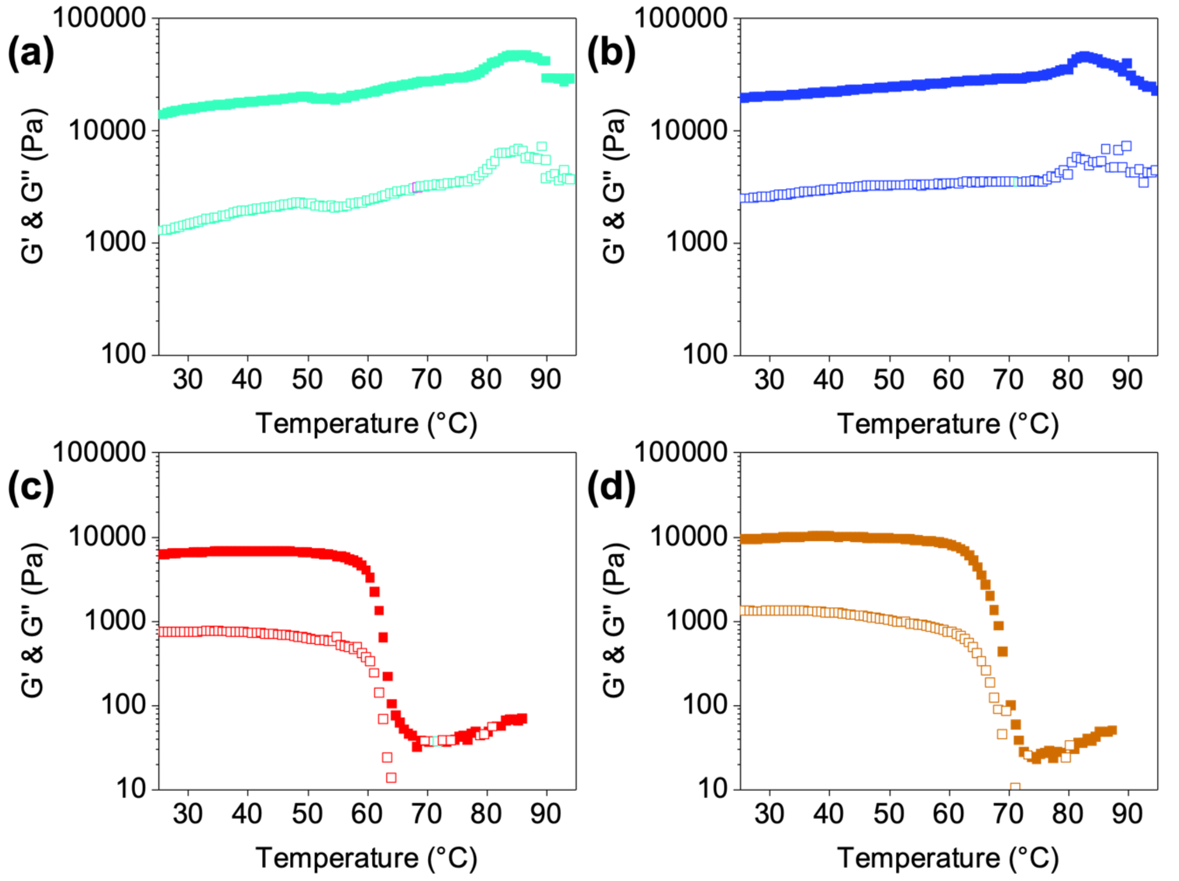

Figure S56. Hydrogel temperature sweeps for (a) **P**, (b) **S**, (c) **P*** and (d) **S***. G' are the solid shapes and G″ are the empty shapes. Tests performed at 0.5% strain and 10 rads^{-1}.

DISCUSSION ABOUT AGING OF P

The stiffness of the **P** gels steadily increases, becoming comparable to **S** gels at ten weeks, Figure S-Figure S. The yield and flow point initially decrease when the bulk solution is two weeks aged, Table S18. After six weeks, both the yield and flow point increase again to a more comparable value to **S** gels, Table S18. This observation suggests that gels become weaker and then stronger.

While bulk rheological properties are more comparable with **S** after aging, the absorbance spectrum of the gel is not, suggesting that the local packing does not change, Figure S. At six weeks of aging, the ratio of peaks at 365 and 385 nm is different to the spectra collected before and after this point, Figure S. This time period is when the strength of the gels increases and suggests a transition state of aging which is more comparable to **S.** After 8 and 10, the absorbance spectra are no longer comparable to **S**, Figure S. The colour of solution gets slightly darker after two weeks but otherwise does not visually change and does not darken to a comparable colour with **S** gels, Figure S.

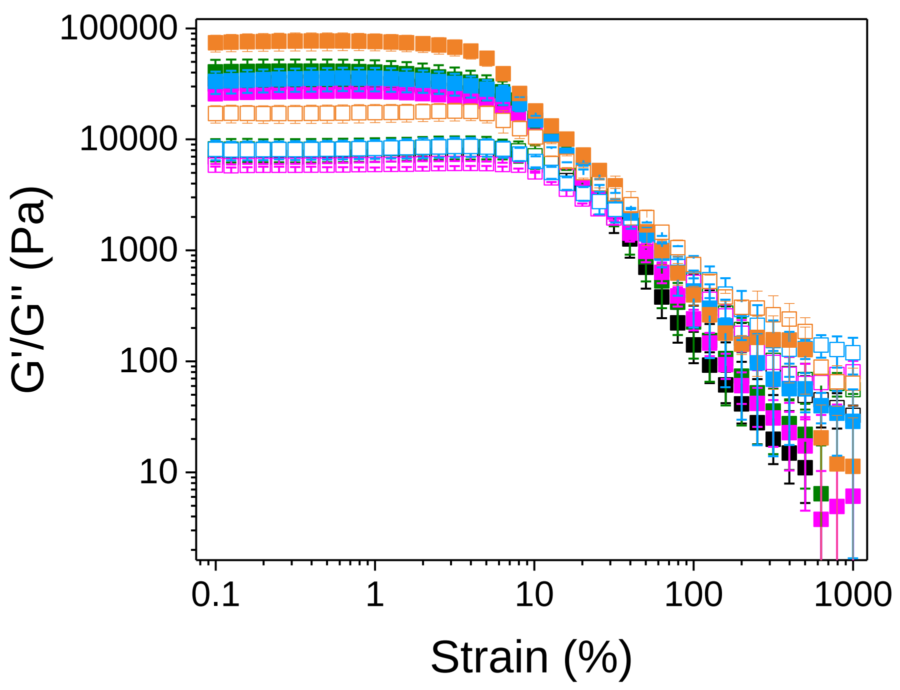

Figure S57. Rheological strain sweeps of gels formed from **P** at 5:5 mg/mL after solution had been allowed to age for 2 (■), 4 (■), 6 (■), 8 (■), 10 (■) weeks. Error bars calculated from the standard deviation of three measurements. G’ are the solid shapes and G″ are the empty shapes.

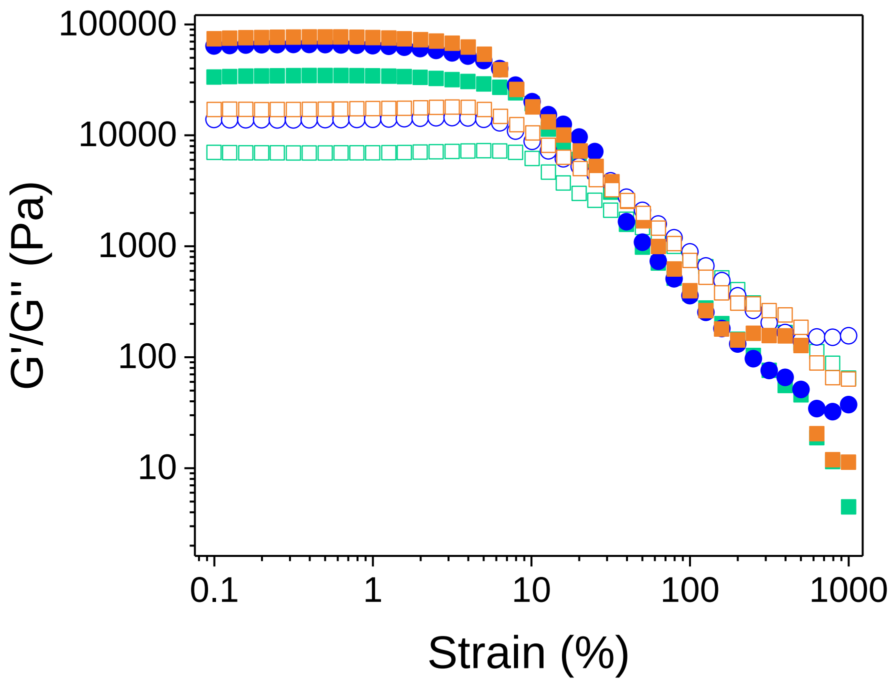

Figure S58. Rheological strain sweeps of gels formed from **S** (●) and **P** at 5:5 mg/mL after solution had been allowed to age for 0 (■) and 10 (■) weeks. Error bars calculated from the standard deviation of three measurements. G' are the solid shapes and G″ are the empty shapes.

Table S18. Tabulated rheological properties taken from an average of three rheological strain sweep measurements of gels formed from **S** and **P** through a kinetics study.

Mix	G"/G' at 0.5% = Tan∂	Yield point (%)	Flow point (%)
**S**	13827/65670 = 0.21	4.0	40.6
**P**	6931/34560 = 0.20	6.3	39.8
**P** 2 weeks	6933/32933 = 0.21	4.0	31.6
**P** 4 weeks	8162/41157 = 0.20	3.2	25.1
**P** 6 weeks	5857/27167 = 0.22	5.0	39.8
**P** 8 weeks	8147/35467 = 0.23	5.1	50.8
**P** 10 weeks	17173/76980 = 0.22	4.0	50.8

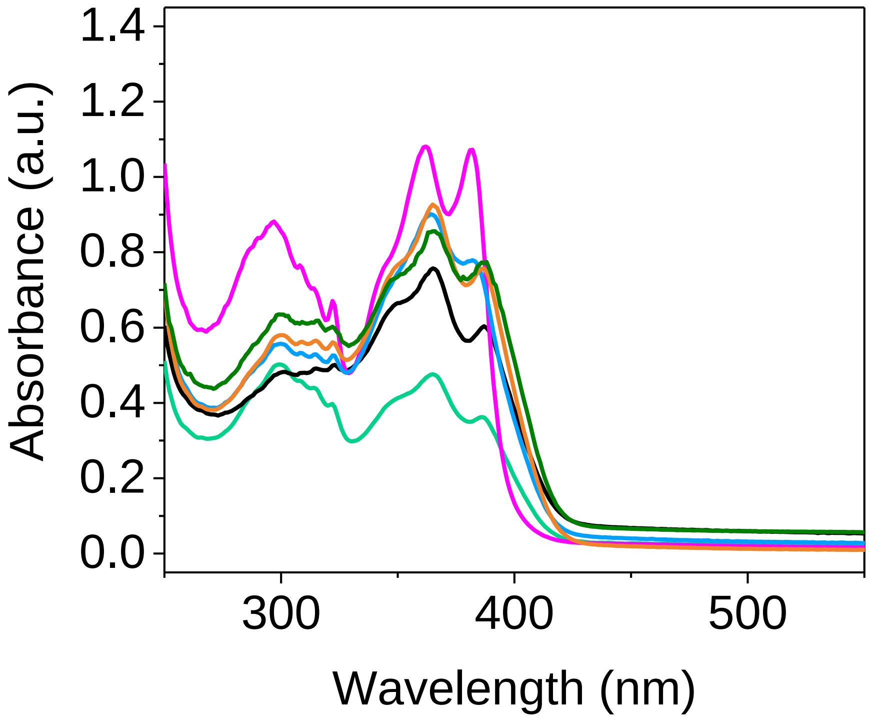

Figure S59. Absorbance spectra of gels formed from **P** at 5:5 mg/mL after solution had been allowed to age for 2 (–), 4 (–), 6 (–), 8 (–), 10 (–) weeks.

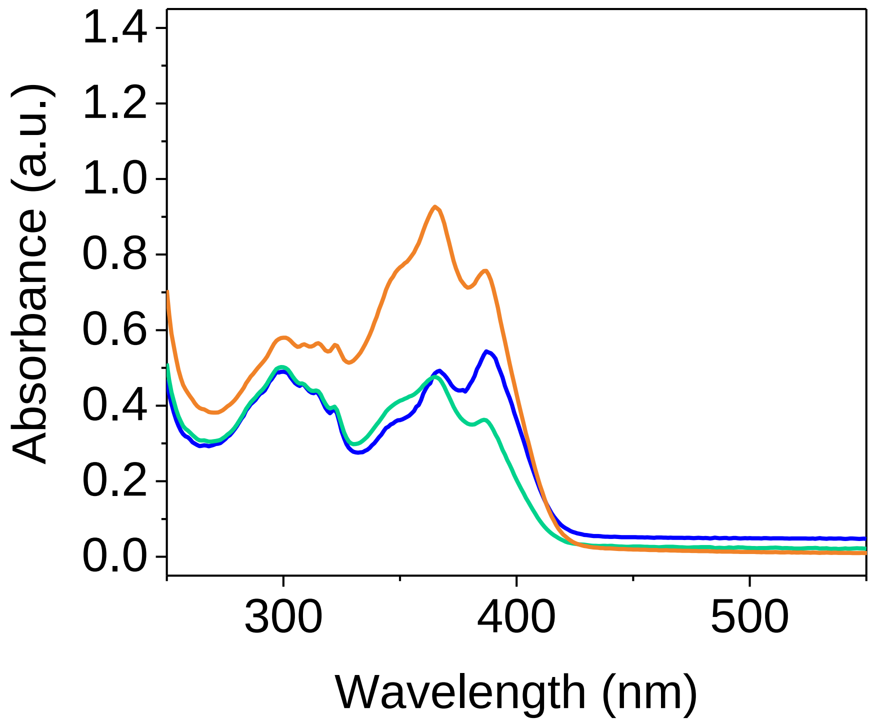

Figure S60. Absorbance spectra of gels formed from **S** (–) and **P** at 5:5 mg/mL after solution had been allowed to age for 0 (–) and 10 (–) weeks.

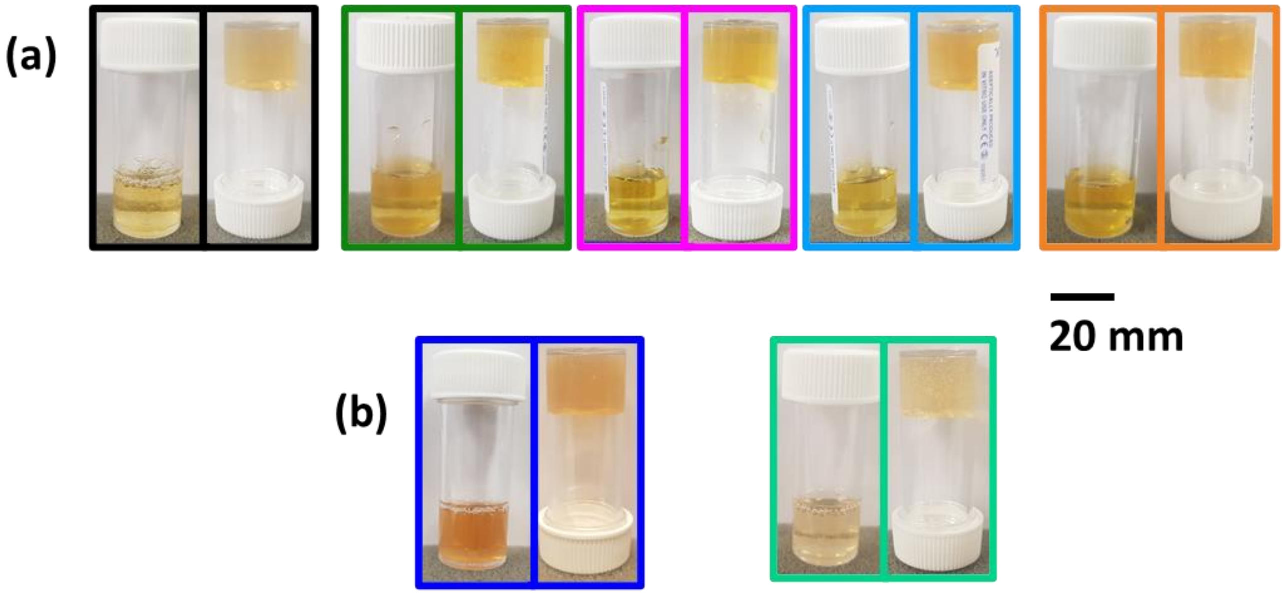

Figure S61. Photographs of solution at pH 11 (left) and gel (right) of (a) **S** (–) and **P** (–) at 5:5 mg/mL and (b) after stock solution had been allowed to age for 2 (–), 4 (–), 6 (–), 8 (–), 10 (–) weeks.

DISCUSSION ABOUT AGING OF SINGLE COMPONENTS

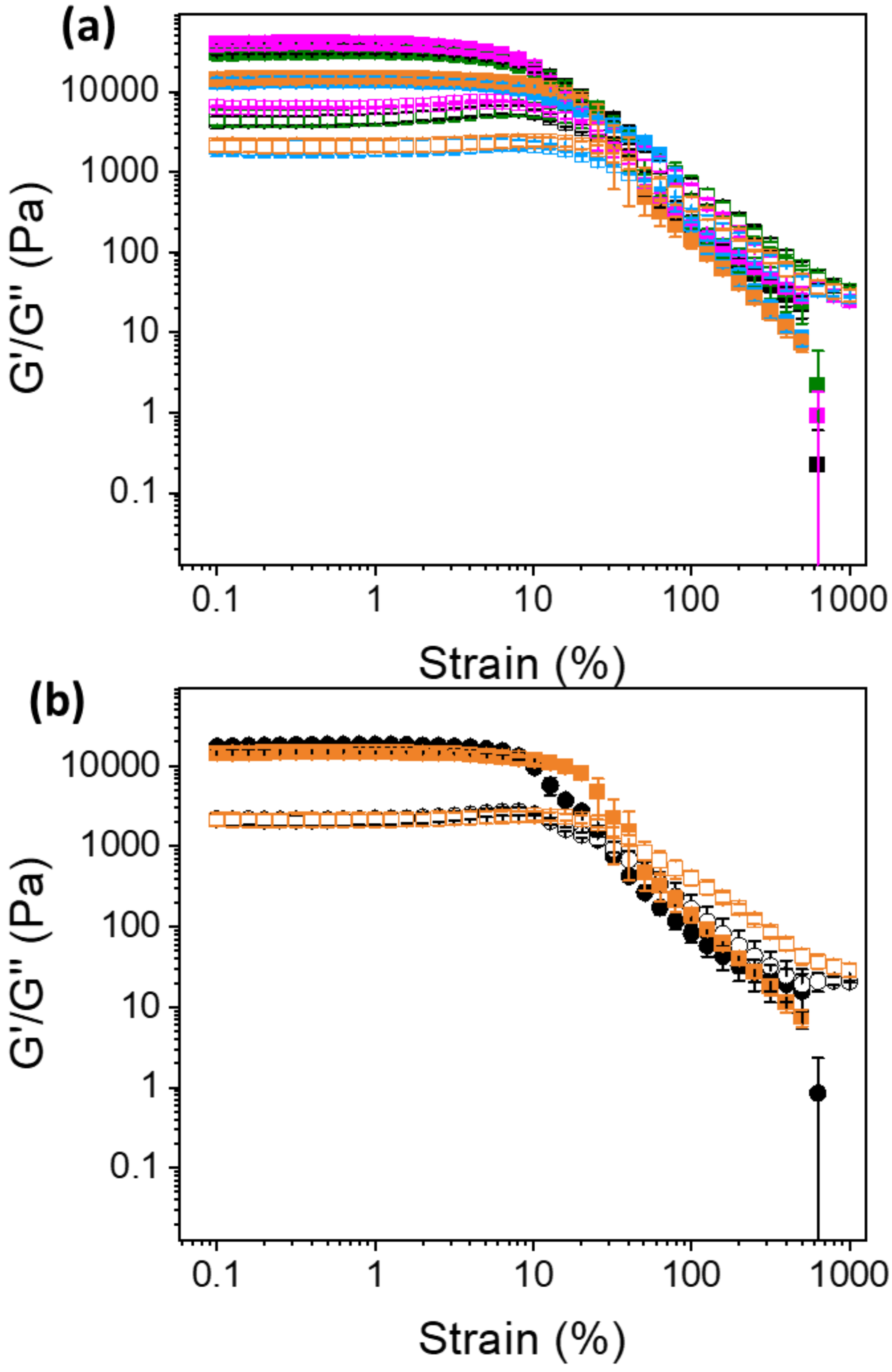

Figure S62. Rheological strain sweeps of gels formed from **1-NapFF** at 5 mg/mL after solution had been allowed to age for (a) 2 (■), 4 (■), 6 (■), 8 (■), 10 (■) weeks and (b) 0 (●) and 10 (■) weeks. Error bars calculated from the standard deviation of three measurements. G' are the solid shapes and G″ are the empty shapes.

Table S19. Tabulated rheological properties taken from an average of three rheological strain sweep measurements of gels formed from **1-NapFF** through a kinetics study.

Gelator (5 mg/mL)	G"/G' at 0.5% = Tan∂	Yield point (%)	Flow point (%)
**1-NapFF**	2158/18323 = 0.12	6.4	31.5
**1-NapFF** 2 weeks	4429/34017 = 0.13	6.4	50.8
**1-NapFF** 4 weeks	4849/31567 =0.15	6.4	32.2
**1-NapFF** 6 weeks	6276/40383 = 0.16	4.0	25.6
**1-NapFF** 8 weeks	2015/13670 = 0.15	6.4	100.00
**1-NapFF** 10 weeks	2127/14713 = 0.14	8.1	50.8

In single component **1-NapFF**, we observe that gels are generally comparable through aging, Figure S. Gels become softer and become stiffer after two-six weeks (but are comparably stiff to initial gels after eight-ten weeks), Table S19. The yield point is comparable through ageing, but the flow point is found at a higher strain at two, eight and ten weeks, suggesting that there are subtle differences in how to gel network breaks down. This trend of an increasing flow point is observed in **P** gels, Table S19. Because the absorbance spectra is poorly defined we cannot observe significant changes with aging in the gels, Figure S. By eye, solutions appear more opaque after approximately eight weeks (when a large flow point is measured), Figure S. This observation suggests that some of the trends observed in **P** kinetics are simply a product of aging.

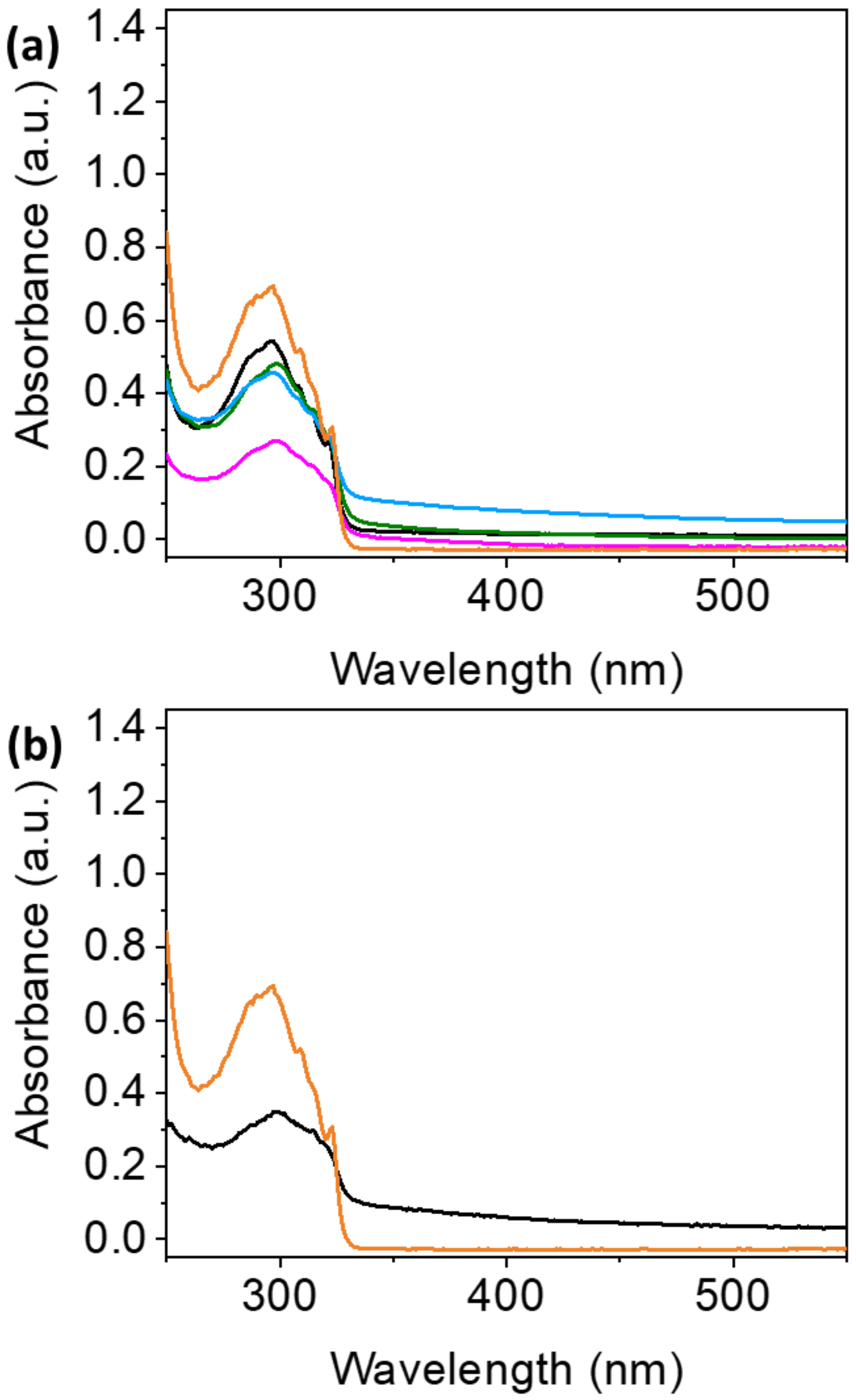

Figure S63. Absorbance spectra of gels formed from **1-NapFF** at 5 mg/mL after solution had been allowed to age for (a) 2 (–), 4 (–), 6 (–), 8 (–), 10 (–) weeks and (b) 0 (–) and 10 (–) weeks.

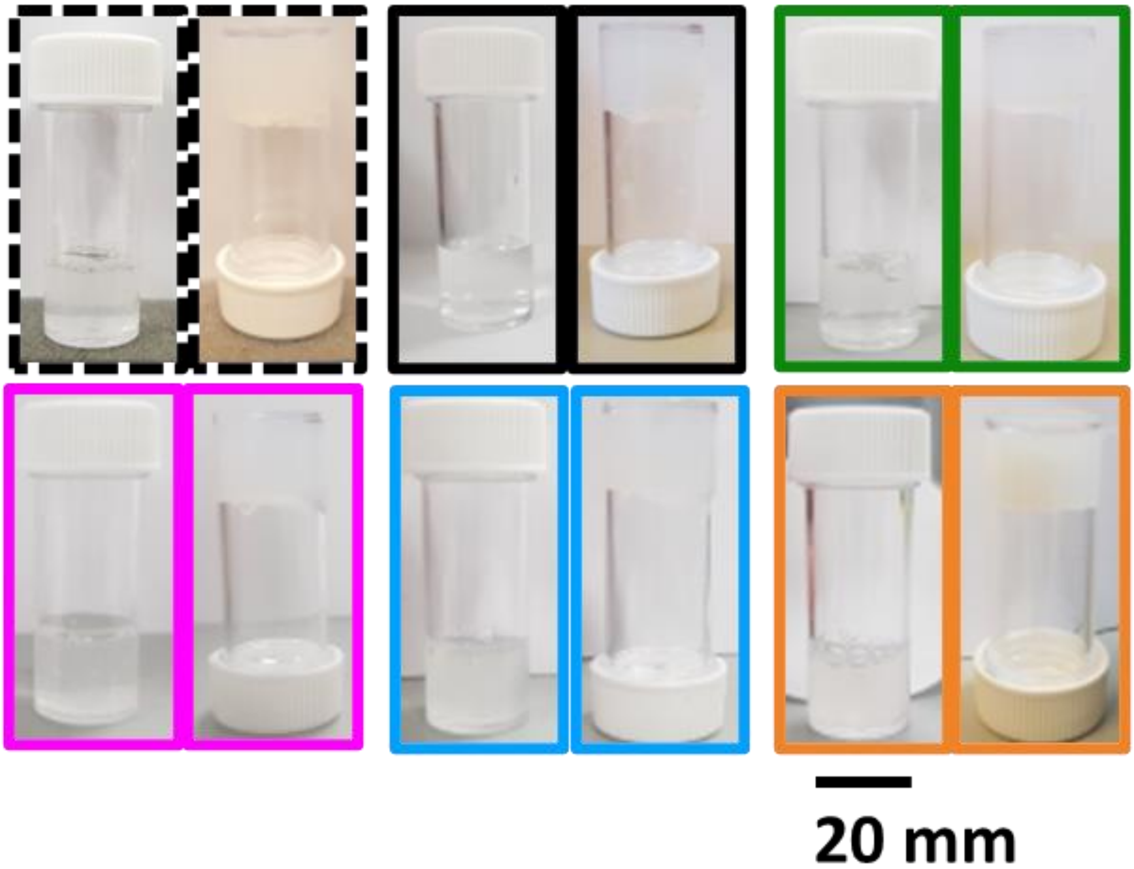

Figure S64. Photographs of solution at pH 11 (left) and gel (right) of **1-NapFF** at 5 mg/mL after stock solution had been allowed to age for 0 (- - -), 2 (–), 4 (–), 6 (–), 8 (–), 10 (–) weeks.

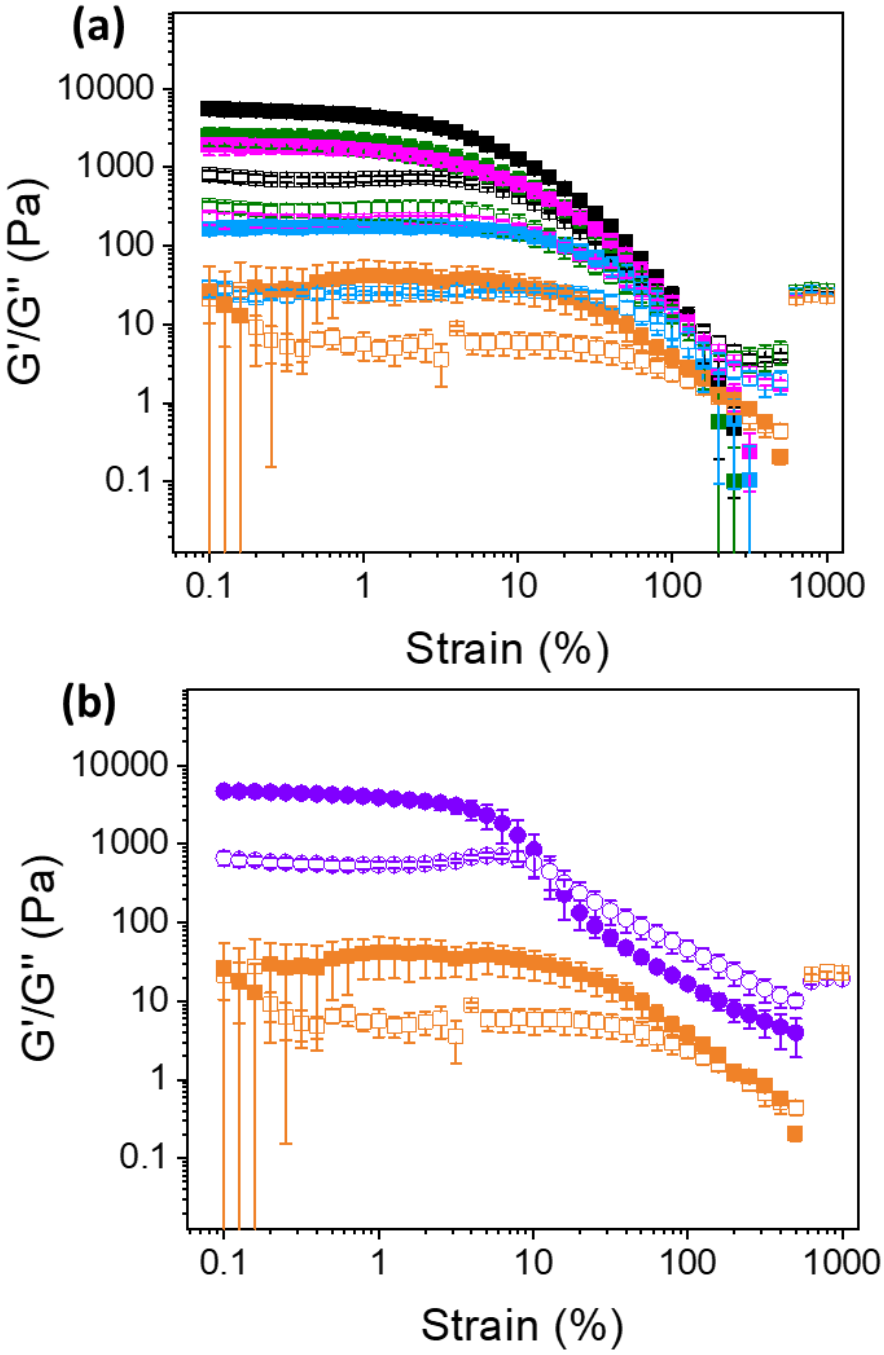

Figure S65. Rheological strain sweeps of gels formed from **NDI-GF** at 5 mg/mL after solution had been allowed to age for (a) 2 (■), 4 (■), 6 (■), 8 (■), 10 (■) weeks and (b) 0 (●) and 10 (■) weeks. Error bars calculated from the standard deviation of three measurements. G’ are the solid shapes and G″ are the empty shapes.

Table S20. Tabulated rheological properties taken from an average of three rheological strain sweep measurements of gels formed from **NDI-GF** through a kinetics study.

Gelator (5 mg/mL)	G"/G' at 0.5% = Tan∂	Yield point (%)	Flow point (%)
**NDI-GF**	544/4279 = 0.13	2.5	15.9
**NDI-GF** 2 weeks	688/4907 = 0.14	1.2	159
**NDI-GF** 4 weeks	285/2268 = 0.13	1.6	159
**NDI-GF** 6 weeks	204/1771 = 0.12	2.5	159
**NDI-GF** 8 weeks	25/173 = 0.14	8.1	158
**NDI-GF** 10 weeks	6.57/34.1 = 2.60	N/A	501

In single component **NDI-GF** gels, a complete breakdown of the gel network is observed. After four weeks, the gels were not stable to inversion and stiffness decreases significantly between weeks two and four, weeks six and eight and weeks eight and ten, Figure S and Figure S. By week ten, there is no linear region in the strain sweep, Figure S and Tan Delta is very large (meaning **NDI-GF** is incapable of forming gels when solution is left to age), Table S20. Between two and four weeks, yield point is lower than initially measured and higher at eight weeks, Table S20 However, the 'gels' are not self-supporting after four weeks, so these measurements may just be of solid-like structures rather than true gels. The flow point is very high even after two weeks which suggests there is a change in the gel forming ability of **NDI-GF** very soon into ageing. The high flow point suggests that gels do not break fully but "cream", Figure S.

After two and eight weeks, the ratio of ratio of peaks at 365 and 385 nm is very subtly different in gels but not significantly as observed in **P**, Figure S. This observation suggests that packing is changing slightly with ageing which could be why gels are struggling to form. At eight and ten weeks, the absorbance intensity is much lower because the 'gel' does not fully form and precipitation occurs, causing issues with the measurement, Figure S. The colour of solution gets slightly darker after two weeks as observed in **P**, Figure S.

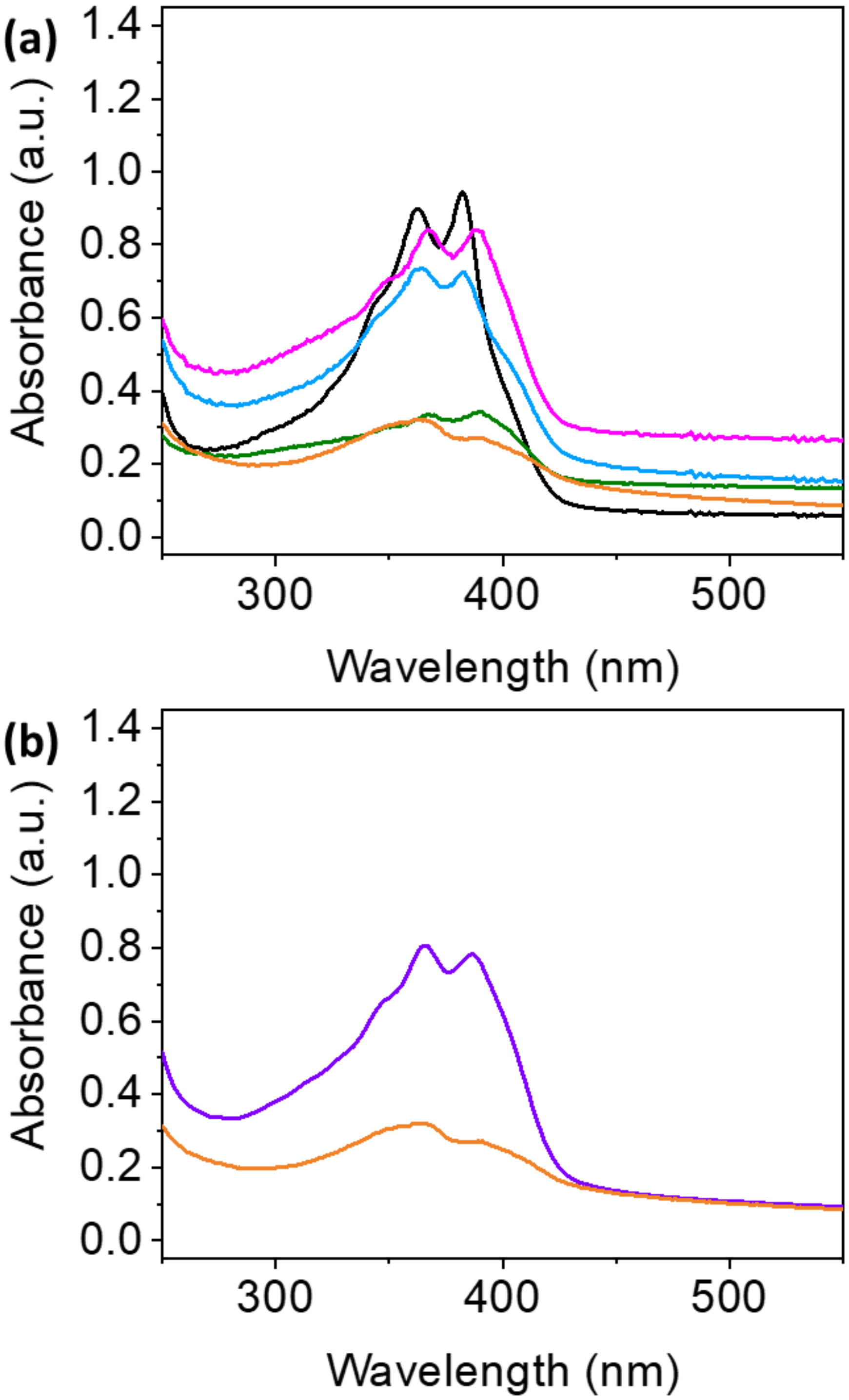

Figure S66. Absorbance spectra of gels formed from **NDI-GF** at 5 mg/mL after solution had been allowed to age for (a) 2 (–), 4 (–), 6 (–), 8 (–), 10 (–) weeks and (b) 0 (–) and 10 (–) weeks.

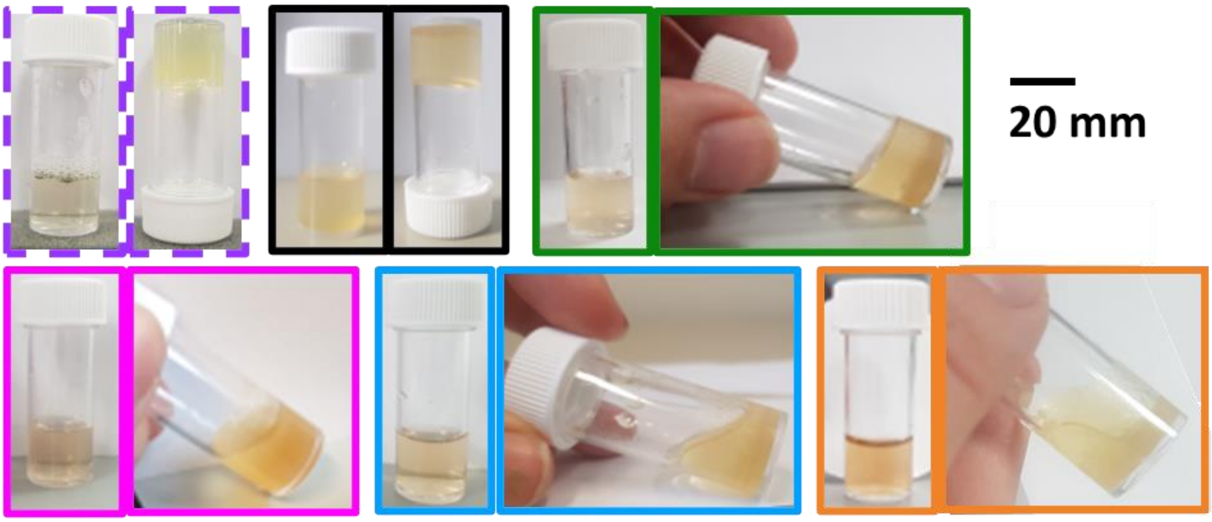

Figure S67. Photographs of solution at pH 11 (left) and gel (right) of **NDI-GF** at 5 mg/mL after stock solution had been allowed to age for 0 (- - -), 2 (–), 4 (–), 6 (–), 8 (–), 10 (–) weeks.

DISCUSSION ABOUT AGING OF P*

The yield and flow point of **P*** gels increased as the solution aged, Figure S, Table S21. This observation is more likely a result of the solution aging as similar trends are observed in **1-NapFF** single component aging studies, Table S19. Both **P** and **P*** have ageing of rheological properties similar to **1-NapFF** as a single component which suggests the observations are a result of aging rather than a change in component interaction. The absorbance spectrum after ten weeks of the gel was roughly comparable to that of the gel made when solution was freshly prepared, Figure S-Figure S. Two poorly defined peaks are observed in the broad absorbance at 365 nm but are not comparable to the spectrum of the **S*** gel. The rheological measurements are comparable after 10 weeks when accounting for aging solution, Figure S. The colour of the solutions and gels do not change over the course of ten weeks, Figure S.

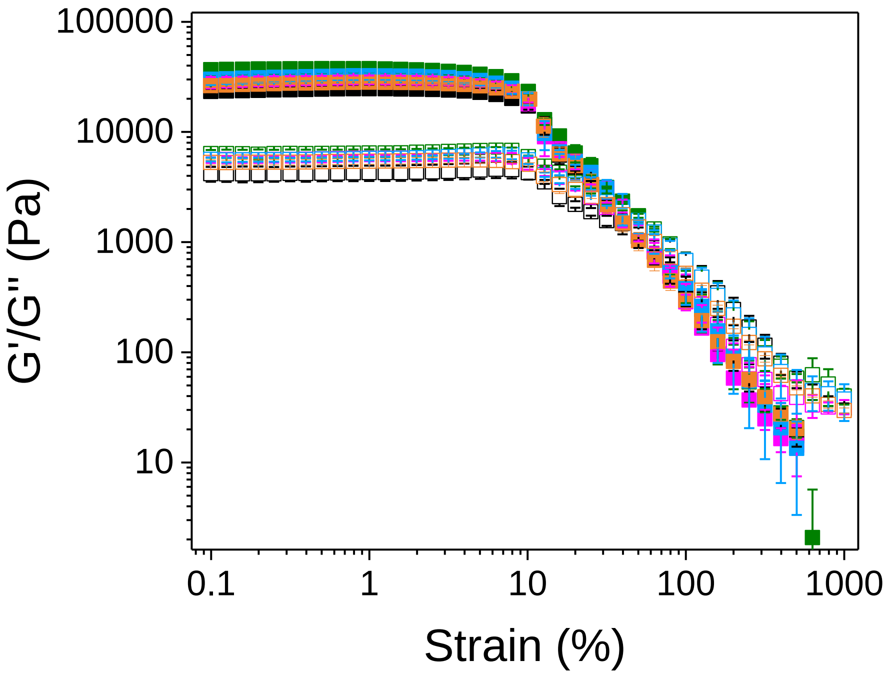

Figure S68. Rheological strain sweeps of gels formed from **P*** at 5:5 mg/mL after solution had been allowed to age for 2 (■), 4 (■), 6 (■), 8 (■), 10 (■) weeks. Error bars calculated from the standard deviation of three measurements. G’ are the solid shapes and G″ are the empty shapes.

Table S21. Tabulated rheological properties taken from an average of three rheological strain sweep measurements of gels formed from **S*** and **P*** through a kinetics study.

Mix	G"/G' at 0.5% = Tan∂	Yield point (%)	Flow point (%)
**S***	1457/11900 = 0.12	6.2	40.1
**P***	4914/26180 = 0.19	6.3	31.6
**P*** 2 weeks	4219/24420 = 0.17	8.0	50.1
**P*** 4 weeks	6370/37500 = 0.17	8.0	63.1
**P*** 6 weeks	5680/29500 = 0.19	8.0	50.1
**P*** 8 weeks	5662/31917 = 0.18	8.0	50.1
**P*** 10 weeks	5363/27967 = 0.19	8.0	40.0

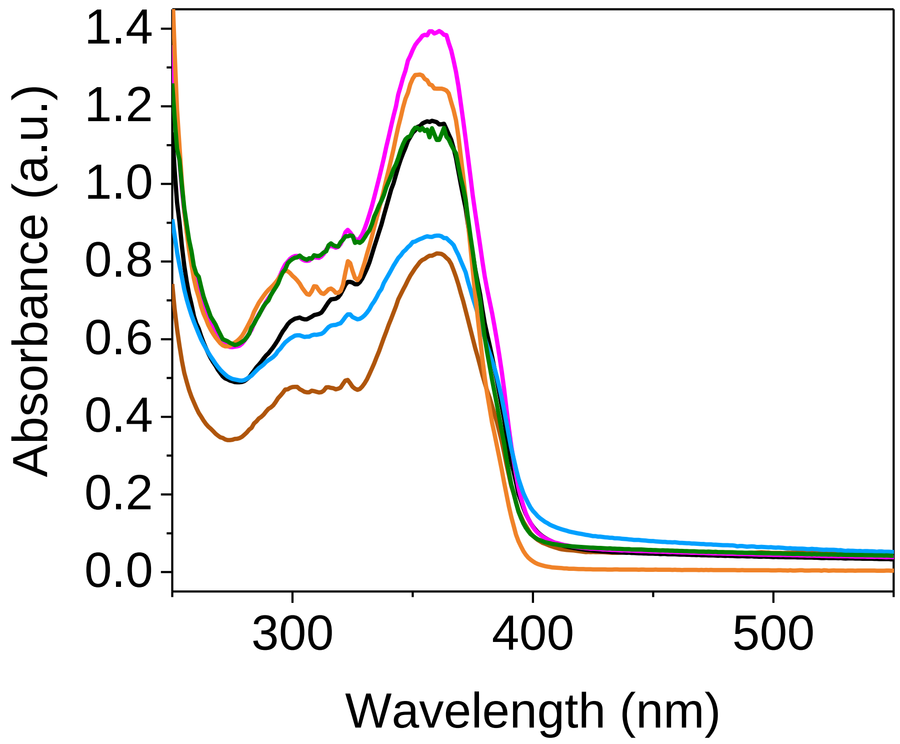

Figure S69. Absorbance spectra of gels formed from **P*** at 5:5 mg/mL after solution had been allowed to age for 2 (–), 4 (–), 6 (–), 8 (–), 10 (–) weeks.

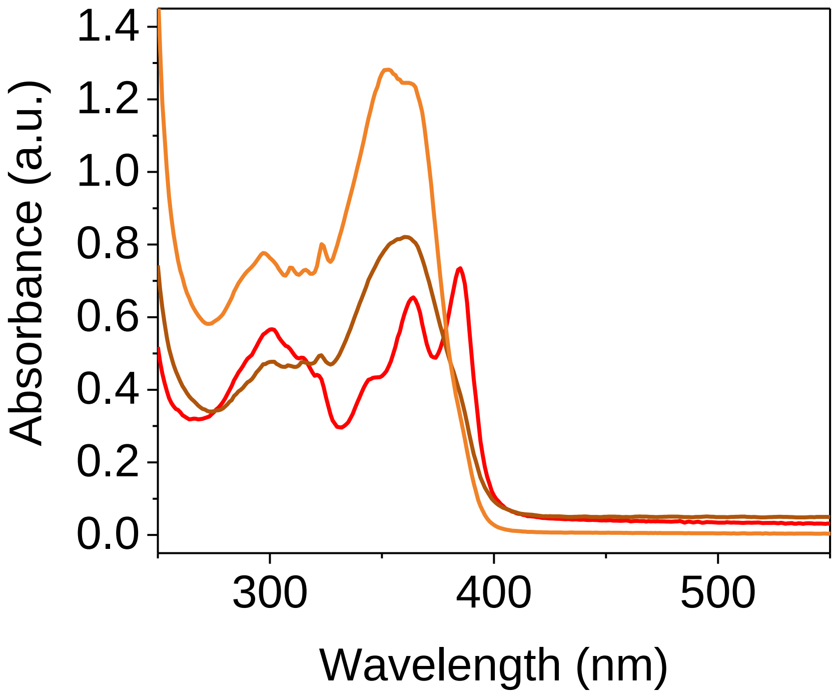

Figure S70. Absorbance spectra of gels formed from **S*** (–) and **P*** at 5:5 mg/mL after solution had been allowed to age for 0 (–) and 10 (–) weeks.

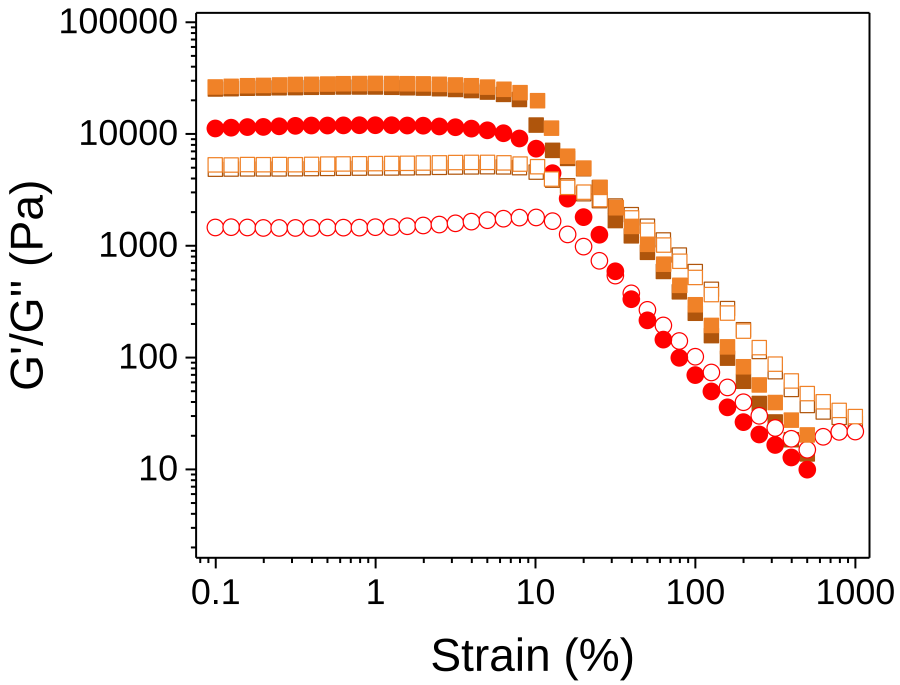

Figure S71. Rheological strain sweeps of gels formed from **S*** (•) and **P*** at 5:5 mg/mL after solution had been allowed to age for 0 (■) and 10 (■) weeks. Error bars calculated from the standard deviation of three measurements. G' are the solid shapes and G″ are the empty shapes.

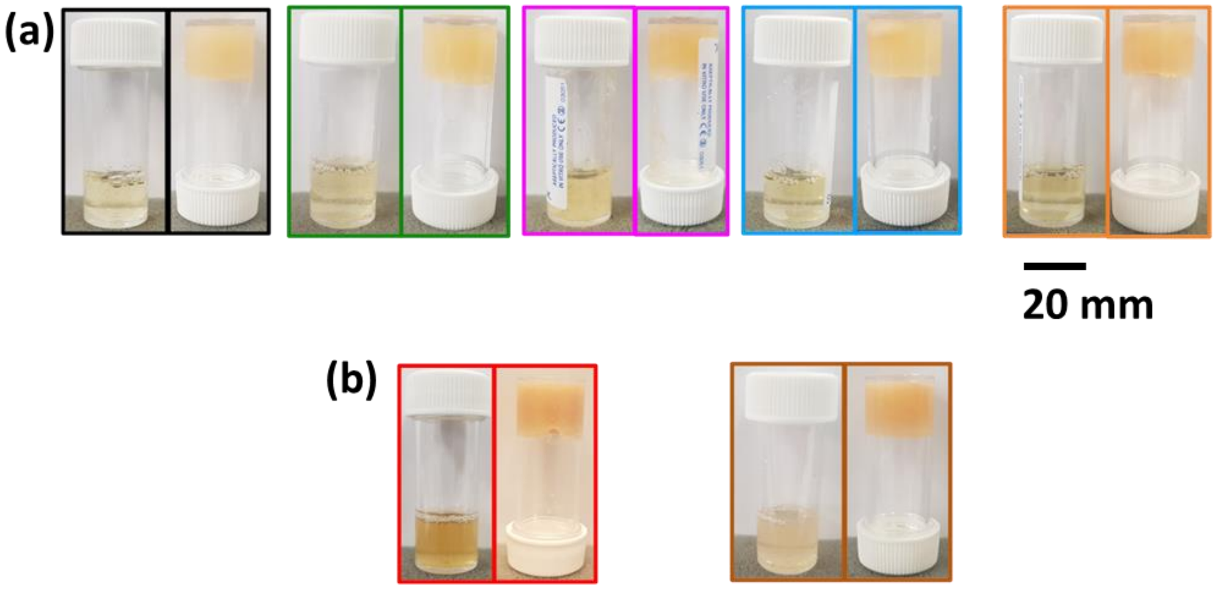

Figure S72. Photographs of solution at pH 11 (left) and gel (right) of (a) **S*** (–) and **P*** (–) at 5:5 mg/mL and (b) after stock solution had been allowed to age for 2 (–), 4 (–), 6 (–), 8 (–), 10 (–)

4. References

1 L. Gonzalez, C. Liu, B. Dietrich, H. Su, S. Sproules, H. Cui, D. Honecker, D. J. Adams and E. R. Draper, *Communications Chemistry*, 2018, **1**, 77.

2 R. I. Randle, L. Cavalcanti, S. Sproules and E. R. Draper, *Materials Advances*, 2022, **3**, 3326–3331.

3 S. Mollick, S. Mukherjee, D. Kim, Z. Qiao, A. v. Desai, R. Saha, Y. D. More, J. Jiang, M. S. Lah and S. K. Ghosh, *Angew. Chem., Int. Ed.*, 2019, **58**, 1041–1045.

4 M. B. Avinash, K. V. Sandeepa and T. Govindaraju, *ACS Omega*, 2016, **1**, 378–387.

5 Z. Li, L. Rösler, K. Herr, M. Brodrecht, H. Breitzke, K. Hofmann, H. Limbach, T. Gutmann and G. Buntkowsky, *Chempluschem*, 2020, **85**, 1737–1746.

6 S. A. Boer and D. R. Turner, *Cryst. Growth Des.*, 2016, **16**, 6294–6303.

7 S. P. Goskulwad, D. D. La, R. S. Bhosale, M. al Kobaisi, S. v. Bhosale and S. v. Bhosale, *RSC Adv.*, 2016, **6**, 39392–39395.

8 M. Pandeeswar, M. B. Avinash and T. Govindaraju, *Chem. - Eur. J.*, 2012, **18**, 4818–4822.

9 R. I. Randle, L. Cavalcanti, S. Sproules and E. R. Draper, *Materials Advances*, , DOI:10.1039/D2MA00207H.

10 G. Andric, A. F. John Boas, B. M. Alan Bond, D. D. Gary Fallon, A. P. Kenneth Ghiggino, C. F. Conor Hogan, A. A. James Hutchison, C. Marcia A-P Lee, A. J. Steven Langford, D. R. John Pilbrow, B. J. Gordon Troup and C. P. Woodward A, *Aust. J. Chem*, 2004, **57**, 1011–1019.

11 B. S. Jursic and P. K. Patel, *Carbohydr. Res.*, 2005, **340**, 1413–1418.

12 Y. Fan, L. Cheng, C. Liu, Y. Xie, W. Liu, Y. Li, X. Li, Y. Li and X. Fan, *RSC Adv.*, 2014, **4**, 52245–52249.

13 L. Chen, S. Revel, K. Morris, L. C. Serpell and D. J. Adams, *Langmuir*, 2010, **26**, 13466–13471.

14 L. Chen, K. Morris, A. Laybourn, D. Elias, M. R. Hicks, A. Rodger, L. Serpell and D. J. Adams, *Langmuir*, 2010, **26**, 5232–5242.

15 G. Pont, L. Chen, D. G. Spiller and D. J. Adams, *Soft Matter*, 2012, **8**, 7797–7802.

16 L. J. Marshall, O. Matsarskaia, R. Schweins and D. J. Adams, *Soft Matter*, 2021, **17**, 2022.

17 Automation Software | Automated Acquisition | Bruker, https://www.bruker.com/en/products-and-solutions/mr/nmr-software/icon-nmr.html, (accessed January 10, 2022).

18 Sasview, Version 4.2.2, www.sasview.org, (accessed January 2, 2020).
